# HDAC5 deficiency induces intrinsic resistance to KRAS inhibition by disrupting c-Myc acetylation-ubiquitination homeostasis

**DOI:** 10.1172/JCI195814

**Published:** 2025-12-11

**Authors:** Taoyu Chen, Haixin Yu, Keshan Wang, Gengdu Qin, Yuhan Zhao, Xueyi Liang, Yuxuan Li, Tianhao Zou, Jiaying Liu, Jingyuan Zhao, Zhiqiang Liu, Ruozheng Wei, Bo Wang, Shanmiao Gou, Tao Yin, Heshui Wu, Xin Jin, Yingke Zhou

**Affiliations:** 1 Department of Pancreatic Surgery,; 2 Sino-German Laboratory of Personalized Medicine for Pancreatic Cancer,; 3 Cancer Center,; 4 Department of Urology, and; 5 Department of Pathology, Union Hospital, Tongji Medical College, Huazhong University of Science and Technology, Wuhan, Hubei, China.

**Keywords:** Cell biology, Oncology, Cancer, Drug therapy, Epigenetics

## Abstract

*KRAS* mutations serve as key oncogenic drivers in the initiation and progression of pancreatic ductal adenocarcinoma (PDAC). Despite the advancement of KRAS inhibitors, such as MRTX1133, for PDAC treatment, intrinsic and acquired resistance remain major barriers to their clinical efficacy. This study underscored the role of histone deacetylase 5 (HDAC5) loss in mediating intrinsic resistance to KRAS^G12D^ inhibitors. Mechanistically, HDAC5 promoted c-Myc degradation by deacetylating K148, thereby facilitating NEDD4-mediated ubiquitination at this site. The loss of HDAC5 resulted in hyperacetylation of c-Myc at K148, impeding the ubiquitination and subsequent degradation process of c-Myc following deacetylation. Consequently, c-Myc stability and transcriptional activity were sustained even under KRAS/MEK/ERK pathway inhibition, reinforcing MAPK signaling and promoting cell survival despite KRAS suppression. Our data further demonstrated that pharmacological or genetic inhibition of c-Myc effectively reversed the resistance phenotype mediated by HDAC5 loss, suggesting a therapeutic strategy centered on KRAS-MYC dual-node blockade. Furthermore, the expression levels of HDAC5 and the acetylation status of c-Myc may serve as biomarkers for predicting the therapeutic response to MRTX1133. These findings provide insights into overcoming resistance to KRAS^G12D^ inhibitors and offer potential biomarkers and combinatorial therapeutic strategies for precision treatment of PDAC.

## Abstract 2

HDAC5 loss stabilizes c-Myc by disrupting acetylation-ubiquitination homeostasis, thereby driving resistance to KRAS inhibitors in PDAC and suggesting dual KRAS-MYC targeting as a potential therapeutic strategy.

## Graphical abstract

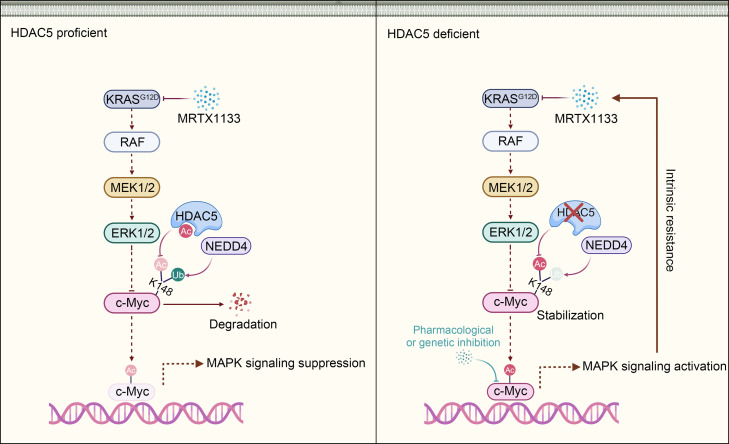

## Introduction

*KRAS* mutations serve as a major genetic driver of pancreatic ductal adenocarcinoma (PDAC) growth and represent one of the earliest key events in tumorigenesis, with approximately 93% of patients with PDAC harboring *KRAS* mutations (1). Among these mutations, the G12D variant is the most prevalent, accounting for approximately 40% (2). KRAS primarily drives PDAC growth through sustained hyperactivation of the RAF/MEK/ERK MAPK signaling pathway (3). Abnormal activation of the MAPK pathway mediated by *KRAS* mutations not only promotes tumor cell proliferation and survival but also leads to systemic dysregulation of transcriptional programs, further enhancing the invasiveness and resistance of PDAC (4). Consequently, precise targeting of KRAS and its downstream MAPK pathway has emerged as a critical therapeutic strategy for PDAC, offering new prospects for improving patient outcomes.

MRTX1133 is a highly efficient and selective noncovalent KRAS^G12D^ inhibitor developed in 2021 (5). Preclinical studies have demonstrated that MRTX1133 exhibits robust antitumor activity in PDAC cell lines, xenograft models, and genetically engineered mouse models (5–7). The strong preclinical performance of MRTX1133 has facilitated its clinical translation, and it is currently undergoing a multicenter phase I/II clinical trial (NCT05737706). However, a key challenge in MRTX1133 treatment is overcoming the intrinsic or acquired resistance. Studies have demonstrated that not all PDAC cells harboring *KRAS^G12D^* mutations are sensitive to MRTX1133, and resistance often develops over time (5, 8, 9). Therefore, a thorough analysis of the resistance mechanisms and the exploration of effective strategies to overcome them are crucial for enhancing the clinical efficacy of MRTX1133 and achieving durable therapeutic responses (10).

c-Myc is one of the most frequently activated oncogenes in human cancers, functioning as a critical transcription factor that regulates the expression of numerous genes, influencing intrinsic cellular processes and the tumor microenvironment (11). Furthermore, studies have demonstrated that c-Myc is also a critical downstream effector transcription factor of the KRAS signaling pathway (12). Additionally, posttranslational modifications such as phosphorylation and acetylation play a crucial role in regulating the protein stability of c-Myc (13–15). Given its short half-life, the protein stability of c-Myc is essential for maintaining its transcriptional activity. KRAS signaling enhances c-Myc stability through ERK1/2-mediated phosphorylation (16). Consequently, under conditions of KRAS signaling inhibition, bypass mechanisms that activate c-Myc are likely to contribute to cellular resistance to KRAS inhibitors.

Histone deacetylase 5 (HDAC5) is a member of the class IIa Zn^2+^-dependent histone deacetylase family. As an important scaffold protein, HDAC5 functions as a “reader” of acetylated lysines and serves as a scaffold for other HDACs to facilitate the deacetylation process (17). It directly or indirectly mediates the deacetylation of various histones and nonhistone substrates, regulating oncogenic signaling pathways and playing a crucial role in tumor cell proliferation, metastasis, metabolism, immune response, and drug sensitivity (18–21). Abnormal expression of HDAC5 has been observed in various cancers, including breast, lung, and colorectal cancers (18). However, in tumors such as PDAC, HDAC5 is often downregulated or deleted, with its expression level closely associated with patient prognosis (19, 20). Nevertheless, the exact function of HDAC5 remains controversial and is not yet fully understood. It may function as either a tumor suppressor or an oncogene, depending on the tissue or specific tumor subtype (19, 20). Given HDAC5’s complex role in cancer, a deeper understanding of its mechanisms may aid in elucidating tumorigenesis and provide a foundation for developing diagnostic and therapeutic strategies.

In this study, we found that HDAC5 deletion promoted intrinsic resistance to MRTX1133 in *KRAS^G12D^*-mutant PDAC. Mechanistically, HDAC5 suppressed c-Myc transcriptional activity by deacetylating c-Myc and promoting its degradation via proteasomal pathway. Deletion of HDAC5 induced hyperacetylation of c-Myc at lysine 148 (K148), which interfered with NEDD4-mediated ubiquitination at the same site. This disruption prevented c-Myc degradation, leading to its stabilization through a KRAS-independent mechanism. Consequently, sustained c-Myc activity persistently activated MAPK signaling, driving resistance to KRAS^G12D^ inhibitors. Furthermore, our study demonstrated that pharmacological or genetic inhibition of c-Myc overcame the intrinsic resistance to KRAS^G12D^ inhibitors in multiple HDAC5-deficient PDAC models, suggesting a promising therapeutic strategy.

## Results

### Loss of HDAC5 induces intrinsic resistance to KRAS inhibitor.

**Figure 1:**
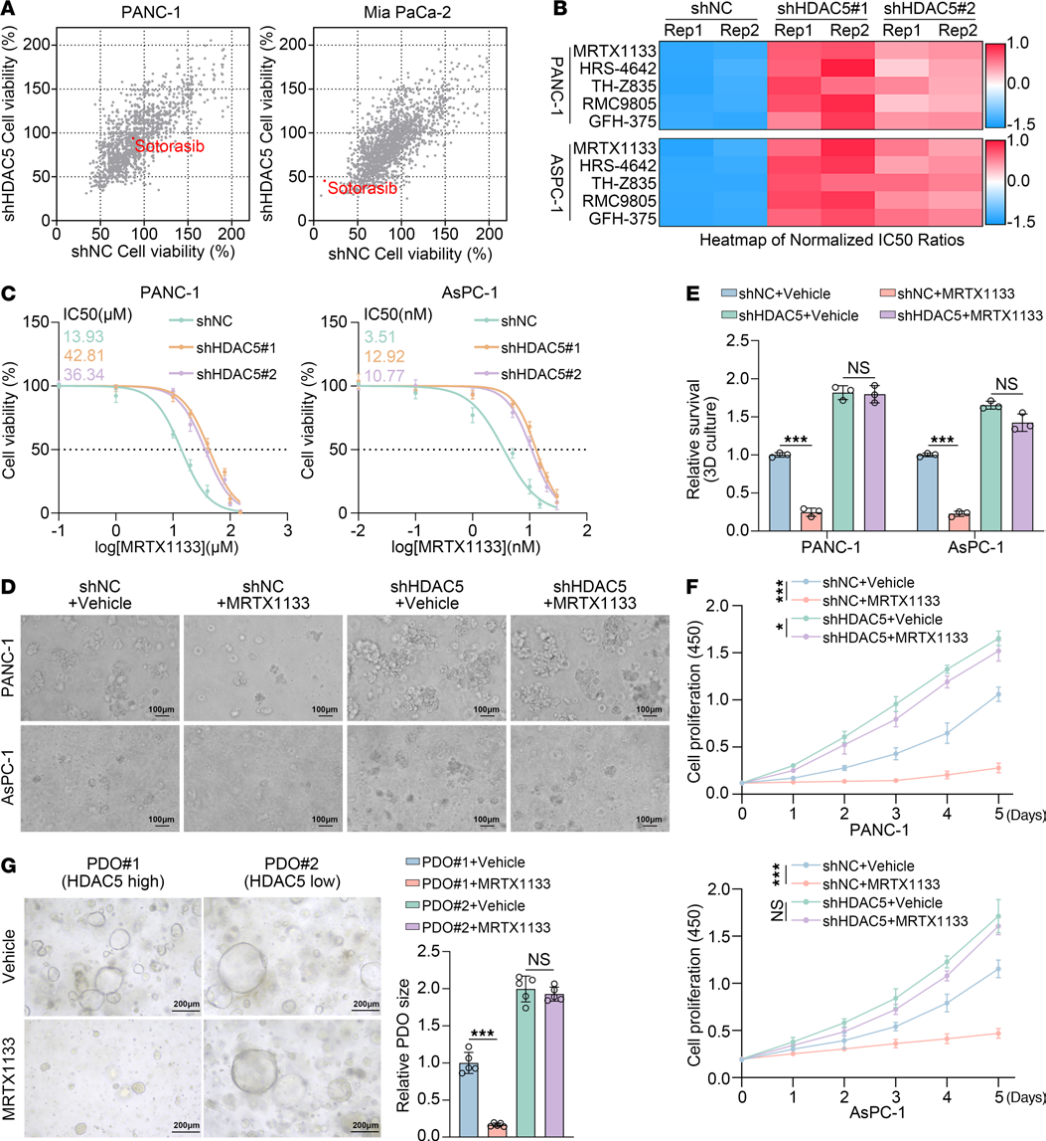
Loss of HDAC5 induces intrinsic resistance to KRAS inhibitor in vitro. (**A**) Scatterplot depicting the antiproliferative effects of a single dose (10 μM) of 1,737 FDA-approved anticancer compounds on PANC-1 and Mia PaCa-2 cells, treated with either short hairpin RNA negative control (shNC) or shHDAC5. (**B**) Heatmap shows the normalized IC_50_ ratio of different KRAS^G12D^ inhibitors determined by cell counting kit 8 (CCK8) assay in PANC-1 and AsPC-1 cells (*n* = 2). The normalized IC_50_ ratio was calculated as the fold-change in IC_50_ of shHDAC5 relative to shNC. (**C**) The IC_50_ of MRTX1133 was assessed by CCK8 assay in PANC-1 and AsPC-1 cells with HDAC5 knockdown. (**D** and **E**) Representative images of 3D-cultured HDAC5-depleted cells treated with DMSO or MRTX1133 (PANC-1: 10 μM; AsPC-1: 5 nM; 48 hours). Scale bars = 100 μm. Relative survival of cells (**E**) (*n* = 3). (**F**) Cell viability of PANC-1 and AsPC-1 cells expressing shNC or shHDAC5 and treated with DMSO or MRTX1133 (10 μM for PANC-1; 5 nM for AsPC-1), measured by CCK8 assay (*n* = 3). (**G**) Representative images and size quantification of PDOs treated with DMSO or MRTX1133 (1 μM) (*n* = 5). All data are presented as the mean ± SD. Statistical significance was determined by 2-way ANOVA followed by Tukey’s multiple comparisons test (**E**–**G**). *P < 0.05, ***P < 0.001.

Our previous studies demonstrated that loss or downregulation of HDAC5 is closely associated with poor prognosis in PDAC and regulates diverse biological processes, including immune responses and fatty acid metabolism (19–21). To further elucidate the function of HDAC5, we conducted a compound library screen of 1,737 FDA-approved drugs across several pancreatic cancer cell lines (22). Among the identified compounds, the KRAS^G12C^ inhibitor sotorasib emerged as a notable hit. As anticipated, sotorasib exerted potent antiproliferative effects in Mia PaCa-2 cells harboring *KRAS^G12C^* mutations; however, these effects were diminished upon HDAC5 knockdown. By contrast, sotorasib had minimal effect on PANC-1 cells harboring *KRAS^G12D^* mutations, irrespective of HDAC5 expression (Figure 1A). Considering that *KRAS^G12D^* mutations occur in approximately 40% of pancreatic cancers — compared with approximately 1% for *KRAS^G12C^* — and there were no KRAS^G12D^-specific inhibitors in the current library, we further investigated whether HDAC5 modulates the sensitivity to KRAS^G12D^ inhibitors in *KRAS^G12D^*-mutant pancreatic cancer cells. We constructed HDAC5-deficient *KRAS^G12D^*-mutant pancreatic cancer cell lines (PANC-1 and AsPC-1) by employing 2 independent HDAC5-specific shRNAs (Supplemental Figure 1A; supplemental material available online with this article; https://doi.org/10.1172/JCI195814DS1). Upon testing several previously reported KRAS^G12D^ inhibitors, including MRTX1133 (5), HRS-4642 (23), TH-Z835 (24), RMC-9805 (25), and GFH-375, we observed that HDAC5 knockdown notably increased the IC_50_ values of PANC-1 and AsPC-1 cells in response to these inhibitors (Figure 1B). MRTX1133, the first highly selective noncovalent inhibitor targeting KRAS^G12D^, shows promising potential for pancreatic cancer treatment and is currently in clinical trials (5, 6). It was thus prioritized for our subsequent investigations. Drug sensitivity analysis showed that HDAC5 knockdown markedly increased MRTX1133 resistance in both PANC-1 and AsPC-1 cells, with IC_50_ values rising from 13.93 μM to 42.81 and 36.34 μM in PANC-1 cells and from 3.51 nM to 12.92 and 10.77 nM in AsPC-1 cells (Figure 1C). 3D cell culture, colony formation, and cell growth curve identified by cell counting kit-8 (CCK8) assay further verified this resistance in both the cell lines examined (Figure 1, D–F, and Supplemental Figure 1, B and C). Additionally, in patient-derived organoids (PDOs), organoids with relatively low HDAC5 expression exhibited increased resistance to MRTX1133, while those with detectable HDAC5 expression were more sensitive to the treatment (Figure 1G and Supplemental Figure 1D). Immunohistochemical (IHC) analysis revealed that MRTX1133 treatment significantly reduced Ki67 levels in organoids with high HDAC5 expression while having minimal effect on Ki67 in organoids with low HDAC5 expression (Supplemental Figure 1, E and F).

**Figure 2:**
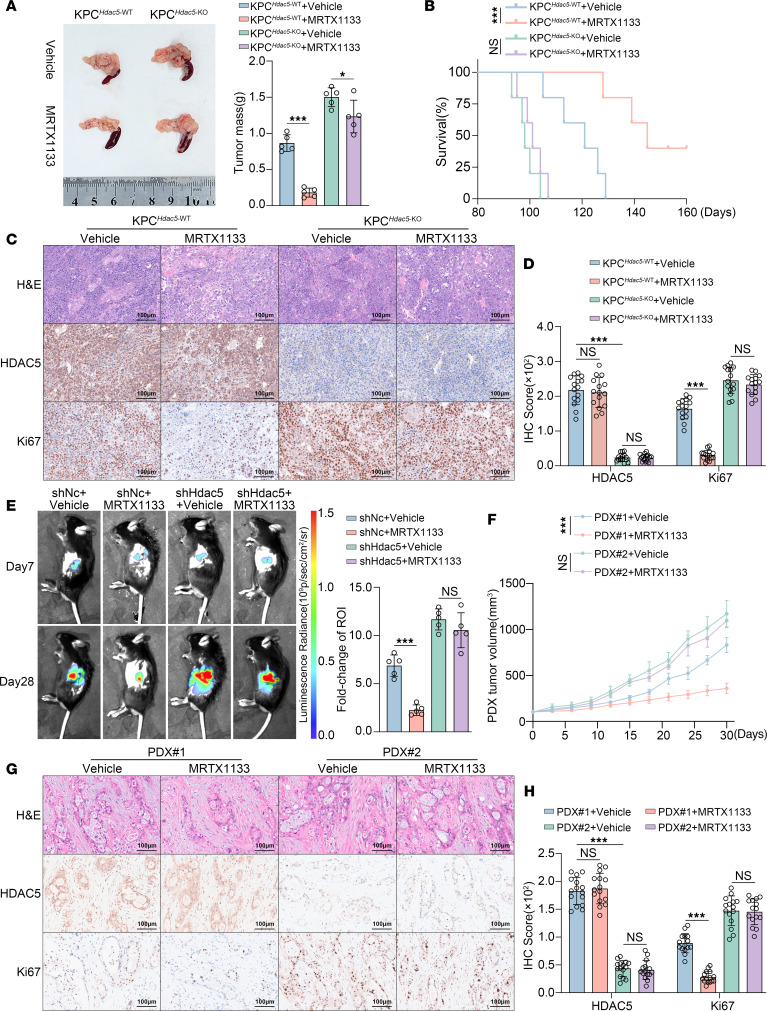
Loss of HDAC5 induces intrinsic resistance to KRAS inhibitor in vivo. (**A**) Representative macroscopic tumor images and tumor weights from KPC^Hdac5-WT^ and KPC^Hdac5-KO^ mouse models treated with vehicle or MRTX1133 (30 mg/kg, i.p., twice daily [bid]) (*n* = 5). (**B**) Kaplan-Meier survival curves with log-rank test (*n* = 5). (**C** and **D**) Representative IHC images of tumors in **C**. IHC scores were quantified in **D**. Scale bars = 100 μm. *n* = 5 biologically independent repeats and 3 independent IHC quantifications. (**E**) C57BL/6 mice were orthotopically injected with KPC-Luc cells expressing shNc or shHdac5. Bioluminescence imaging was performed on day 7, followed by treatment with vehicle or MRTX1133 (30 mg/kg, i.p., bid). Representative bioluminescence images and corresponding quantification were acquired on day 28 (*n* = 5). (**F**) Tumor growth curves in PDX models with treatment with vehicle or MRTX1133 (30 mg/kg, i.p., bid) (*n* = 5). (**G** and **H**) Representative IHC images of PDXs and quantified IHC scores (**H**). Scale bars = 100 μm. *n* = 5 biologically independent repeats and 3 independent IHC quantifications. All data are presented as the mean ± SD. Statistical significance was determined by 2-way ANOVA followed by Tukey’s multiple comparisons test (**A**, **D**–**F**, and **H**). *P < 0.05, ***P < 0.001.

We further validated our findings in PDAC models in vivo. We generated genetically engineered mice with either Hdac5 wild-type (KPC^Hdac5-WT^) or Hdac5-knockout (KPC^Hdac5-KO^) alleles using the KPC [*Kras^tm1(LSL-G12D)^Trp53^tm1(LSL-R172H)^Pdx1*-*Cre*] mouse model (Supplemental Figure 1, G and H). Consistent with our previous observation, tumor burden of the mice was significantly higher in the KPC^Hdac5-KO^ group compared with the KPC^Hdac5-WT^ group (Figure 2A). MRTX1133 treatment significantly reduced tumor burden and extended survival in the KPC^Hdac5-WT^ mice, while no significant effects were observed in the KPC^Hdac5-KO^ mice (Figure 2, A and B). IHC analysis of tumors following treatment revealed that MRTX1133 significantly reduced Ki67 expression in the KPC^Hdac5-WT^ mice, whereas no notable changes were observed in the KPC^Hdac5-KO^ mice (Figure 2, C and D). More importantly, in both the KPC-Luc cell-based orthotopic pancreatic cancer model and patient-derived xenograft (PDX) model, we also observed consistent phenomena: Tumors with HDAC5 deficiency exhibited resistance to MRTX1133, as evidenced by faster tumor growth rates and larger tumor volumes (Figure 2, E–H, and Supplemental Figure 1, I–L). To determine if HDAC5 loss confers broad innate resistance to KRAS inhibition, we evaluated 2 additional clinical trial–stage KRAS^G12D^ inhibitors, with HRS-4642 in KPC mice and GFH-375 in PDX models. Notably, HDAC5 deficiency consistently enhanced intrinsic resistance to both agents across the tested models (Supplemental Figure 2). Consistent with our high-throughput drug screening results, silencing HDAC5 in Mia PaCa-2 cells led to intrinsic resistance to sotorasib (the KRAS^G12C^ inhibitor) in both in vitro and in vivo models (Supplemental Figure 3). Collectively, our data strongly suggest that HDAC5 deficiency induces intrinsic resistance to KRAS inhibitors.

### Loss of HDAC5 upregulates MAPK signaling via c-Myc.

**Figure 3:**
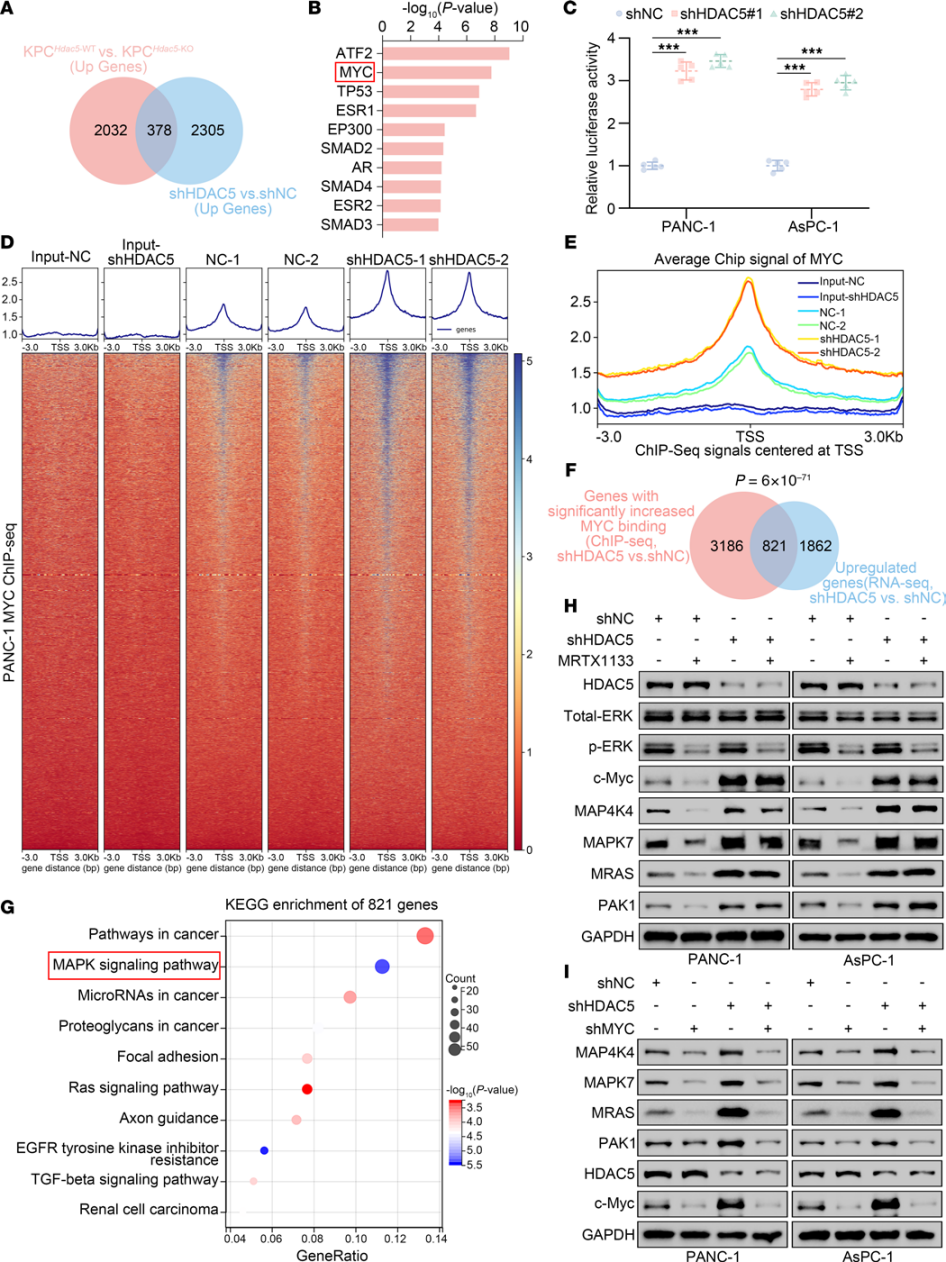
Loss of HDAC5 upregulates MAPK signaling via c-Myc. (**A**) Venn diagram showing the overlap of genes upregulated in KPC^Hdac5-KO^ mice (*n* = 5 per genotype; log_2_[fold-change] > 1, *P* < 0.05, 2,410 genes) identified via RNA-Seq and genes upregulated in shHDAC5-treated PANC-1 cells (*n* = 3 per condition; log_2_[fold-change] > 1, *P* < 0.05, 2,683 genes), revealing a shared subset of 378 genes. (**B**) Bar graph showing the top 10 enriched transcription factors from transcription factor analysis of 378 genes in **A** using Enrichr. ATF2, activating transcription factor 2. (**C**) Dual-luciferase reporter assays were performed to assess the transcriptional activity of c-Myc in HDAC5-depleted PANC-1 and AsPC-1 cells. Data are presented as mean ± SD (*n* = 5). Statistical significance was determined by 1-way ANOVA followed by Dunnett’s multiple comparisons test. ***P < 0.001. (**D**) Heatmap of MYC ChIP-Seq signal intensity (±3 kb around MYC binding sites) in control vs. HDAC5-knockdown PANC-1 cells. (**E**) The average ChIP signal of MYC centered at transcription start site (±3 kb) in indicated groups. (**F**) Venn diagram depicting the overlap between genes with enhanced MYC promoter occupancy after shHDAC5 knockdown, as identified by ChIP-Seq, and genes upregulated in PANC-1 cells following shHDAC5 knockdown, as determined by RNA-Seq. *P* = 6 × 10^–71^. (**G**) KEGG pathway enrichment analysis of 821 intersecting genes in **F**. (**H** and **I**) Western blot analysis of canonical MAPK pathway protein expression under indicated conditions.

To elucidate the mechanism underlying the intrinsic resistance to MRTX1133 induced by HDAC5 deficiency, we performed transcriptomic sequencing of tumor tissues from KPC^Hdac5-KO^ and KPC^Hdac5-WT^ mice, identifying 2,410 upregulated and 2,456 downregulated differentially expressed genes (DEGs) (Supplemental Figure 4, A–C). Kyoto Encyclopedia of Genes and Genomes (KEGG) pathway enrichment analysis of all DEGs revealed a significant enrichment of the MAPK signaling pathway (Supplemental Figure 4D). Additionally, RNA-sequencing (RNA-Seq) analysis of HDAC5-knockdown and control PANC-1 cells identified 2,683 upregulated and 2,396 downregulated DEGs (Supplemental Figure 4, E–G), with KEGG analysis again showing significant enrichment of the MAPK signaling pathway (Supplemental Figure 4H). Given that HDAC5 primarily functions as a transcriptional repressor, we further identified 378 genes that were consistently upregulated upon HDAC5 knockdown in both datasets (Figure 3A). Interestingly, transcription factor enrichment analysis of the 378 upregulated genes revealed a significant increase in MYC transcriptional activity (Figure 3B). Dual-luciferase reporter assays demonstrated that HDAC5 knockdown significantly enhanced the transcriptional activity of c-Myc (Figure 3C). Moreover, ChIP-Seq analysis in PANC-1 cells revealed that HDAC5 knockdown markedly increased the global binding intensity of MYC (Figure 3, D and E). Further analysis showed a significant overlap between genomic loci with increased MYC binding after HDAC5 knockdown (identified by DiffBind analysis) and the genes repressed by HDAC5 identified by RNA-Seq, resulting in the identification of 821 overlapping genes (Figure 3F). KEGG pathway analyses revealed that these overlapping genes were significantly enriched in the MAPK signaling pathway (Figure 3G). These data indicated HDAC5 loss resulted in the activation of MYC and MAPK signaling.

c-Myc, an oncogenic member of MYC proteins, is the key downstream effector molecule in the KRAS^G12D^-mediated activation of the RAF/MEK/ERK (MAPK) signaling cascade (16). If HDAC5 loss activates c-Myc via the RAF/MEK/ERK bypass pathway, that might enable c-Myc to drive the expression of downstream target genes of MAPK signaling even when KRAS^G12D^ is inhibited, thereby promoting cell survival and drug resistance (26). As predicted, ERK activation (phosphorylated ERK, p-ERK) and c-Myc expression were substantially downregulated upon MRTX1133 treatment in HDAC5-proficient PANC-1 cells (Figure 3H), along with the expression of c-Myc downstream genes, including MAP4K4, MAPK7, MRAS, and PAK1 (Figure 3H). However, in HDAC5-knockdown cells, MRTX1133 effectively inhibited p-ERK but failed to suppress c-Myc expression and its downstream genes (Figure 3H), with a marked upregulation of c-Myc following HDAC5 knockdown (Figure 3H). RT-qPCR, dual-luciferase reporter assay, and ChIP-qPCR further validated that, in HDAC5-deficient cells, the luciferase activity of c-Myc, the enrichment of c-Myc at the promoter regions of its target genes, and the expression of these target genes were no longer suppressed by MRTX1133 treatment, as observed in control cells (Supplemental Figure 5, A–C). RNA-Seq analysis further revealed consistent findings. In HDAC5-proficient AsPC-1 cells, MRTX1133 markedly suppressed the expression of MAPK pathway downstream genes. By contrast, HDAC5 knockdown resulted in a marked activation of MAPK pathway, which was not suppressed by the treatment with MRTX1133 (Supplemental Figure 6, A–E). Upon the condition of c-Myc knockdown, HDAC5 knockdown no longer induced the upregulation of the representative downstream genes of MAPK signaling, as elucidated by Western blot and RT-qPCR (Figure 3I and Supplemental Figure 5D). Notably, our prior transcription factor enrichment analysis also revealed significant enrichment of ATF2 (Figure 3B). Prior studies have established ATF2 as a pivotal downstream effector of the MAPK signaling cascade, with its activation predominantly contingent upon MAPK-mediated phosphorylation (27, 28). To investigate whether ATF2 is involved in HDAC5 loss–mediated MAPK activation, we performed Western blot assays and found that HDAC5 depletion markedly augmented ATF2-T71 phosphorylation, and this effect was abolished under MYC inhibition (Supplemental Figure 6, F and G). Thus, we propose that ATF2 activation upon HDAC5 deficiency depends on MYC-mediated MAPK activation.

Additionally, c-Myc knockdown reversed the HDAC5 knockdown–induced resistance to MRTX1133 treatment in both PANC-1 and AsPC-1 cells (Supplemental Figure 6H). Taken together, our data indicate that the loss of HDAC5 upregulates MAPK signaling through c-Myc via a RAF/MEK/ERK bypass mechanism, thereby inducing the intrinsic resistance to MRTX1133.

### HDAC5 promotes c-Myc ubiquitination and degradation through its deacetylation.

**Figure 4:**
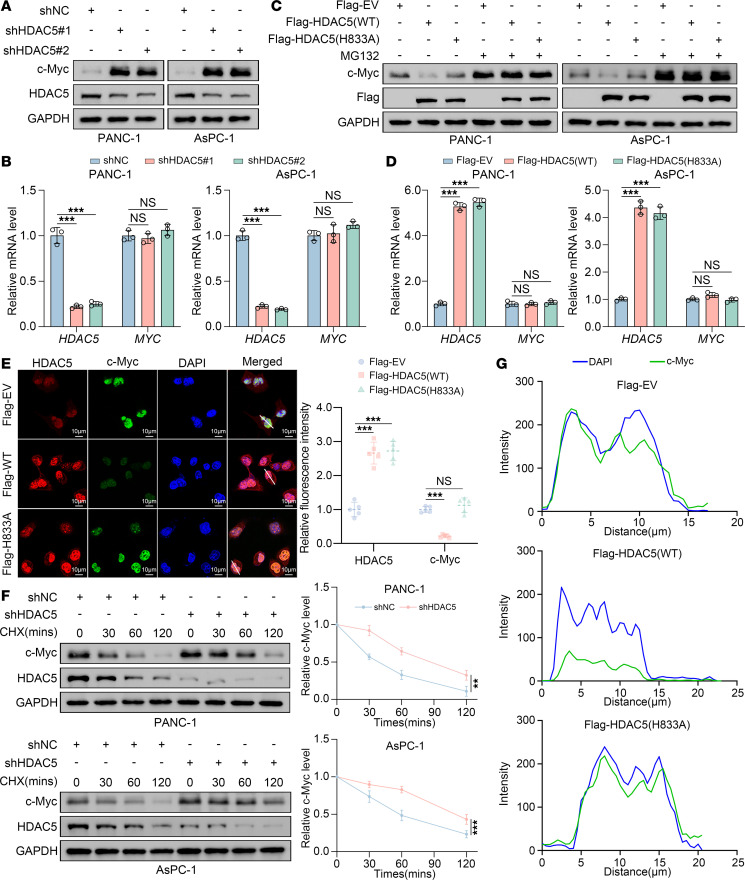
Loss of HDAC5 promotes the stabilization of c-Myc protein. (**A** and **B**) Western blot (**A**) and RT-qPCR (**B**) analyses of c-Myc expression in PANC-1 and AsPC-1 cells infected with indicated shRNAs for 48 hours (*n* = 3). (**C**) Western blot analysis of c-Myc protein levels in PANC-1 and AsPC-1 cells transfected with indicated plasmids and treated with DMSO or MG132 (10 μM, 8 hours). EV, empty vector. (**D**) RT-qPCR analysis of c-Myc mRNA in PANC-1 and AsPC-1 cells transfected with indicated plasmids for 48 hours (*n* = 3). (**E**) Immunofluorescence analysis of c-Myc in PANC-1 cells transfected with indicated plasmids for 48 hours. Representative images and fluorescence intensity quantification are shown (*n* = 5). (**F**) Western blot analysis and quantification of c-Myc protein stability in control or HDAC5-knockdown PANC-1 and AsPC-1 cells treated with cycloheximide (CHX, 50 μg/mL) for indicated times (*n* = 3). (**G**) Colocalization analysis of the merged images in **E**, showing pixel intensity profiles along the white line from left to right in each panel. Colors correspond to the merged images: green for c-Myc and blue for DAPI. All data are presented as the mean ± SD. Statistical significance was determined by 1-way ANOVA followed by Dunnett’s multiple comparisons test (**B**, **D**, and **E**) or 2-way ANOVA followed by Tukey multiple comparisons test (**F**). **P < 0.01, ***P < 0.001.

As previously observed, HDAC5 knockdown resulted in a prominent upregulation of c-Myc expression (Figure 3H). Further validation in PANC-1 and AsPC-1 cells showed that HDAC5 knockdown led to a marked increase in c-Myc protein expression without a substantial change in *MYC* mRNA levels (Figure 4, A and B). Consistently, ectopic overexpression of WT HDAC5 downregulated c-Myc expression specifically at the protein level, whereas the enzymatically inactivated HDAC5 (H833A) mutant failed to exert this effect, suggesting a mechanism dependent on HDAC5’s enzymatic activity (Figure 4, C and D). Furthermore, treatment with the proteasome inhibitor MG132 substantially upregulated c-Myc protein levels and counteracted the downregulation induced by HDAC5 overexpression, consistent with the reported degradation of c-Myc via the ubiquitin-proteasome system (Figure 4, C and D) (29, 30). Immunocytochemistry further verified that only WT-HDAC5 overexpression, but not the HDAC5 (H833A) mutant, repressed endogenous c-Myc expression (Figure 4E), whereas HDAC5 depletion markedly increased endogenous c-Myc protein levels (Supplemental Figure 7A). In line with these observations, HDAC5 knockdown prolonged the half-life of c-Myc protein in both tested cell lines (Figure 4F), indicating that HDAC5 loss enhances c-Myc protein stability.

Previous studies have demonstrated that c-Myc acetylation in neurons diminishes nuclear accumulation and facilitates calpain-mediated proteolysis (31). To assess whether this mechanism applies in pancreatic cancer cells, we performed immunofluorescence colocalization and nuclear-cytoplasmic fractionation assays, and we found that HDAC5 manipulation does not affect its subcellular distribution (Figure 4G and Supplemental Figure 7, A–C).

**Figure 5:**
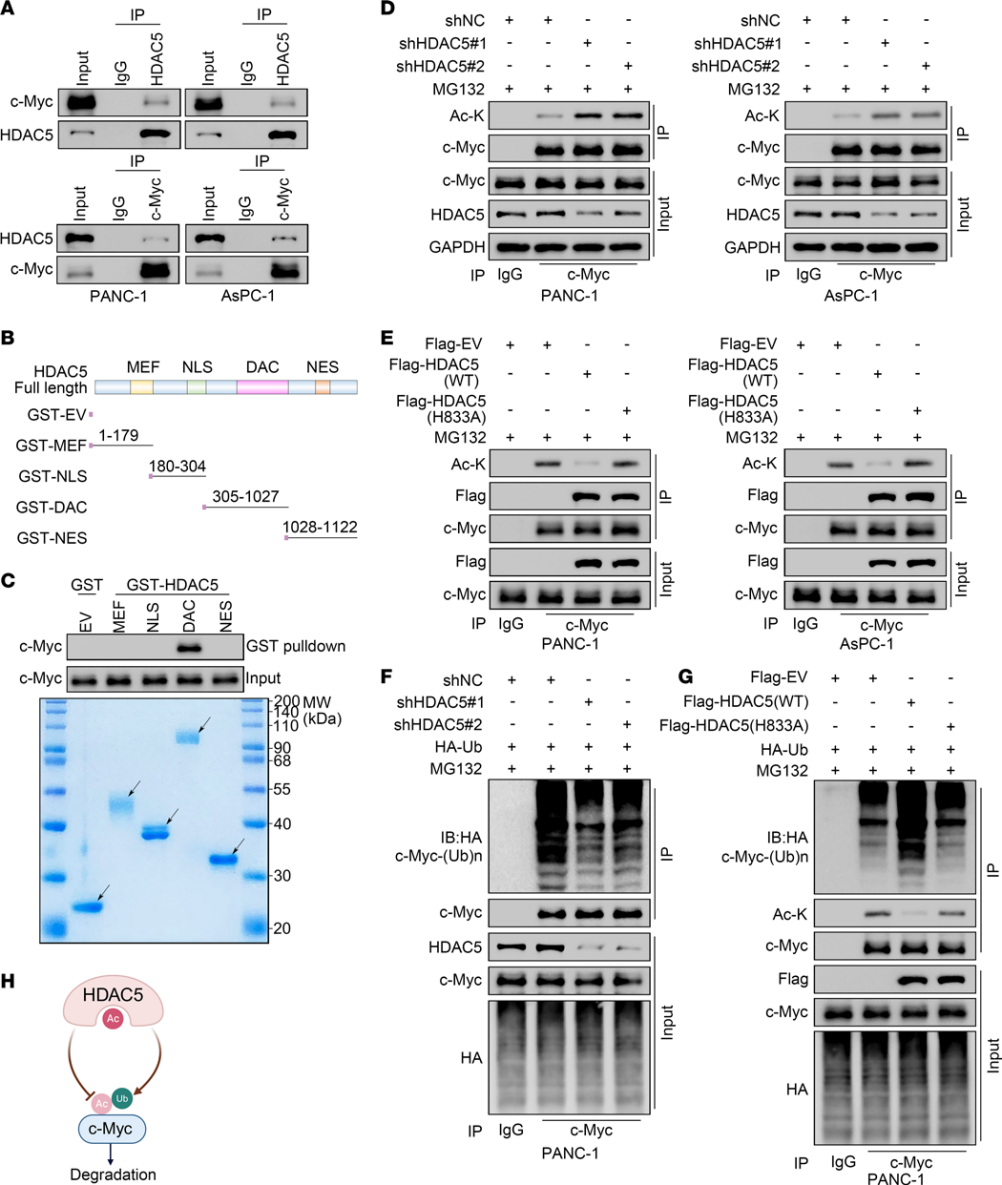
HDAC5 promotes c-Myc ubiquitination and degradation through its deacetylation. (**A**) Co-IP assay showing interaction between c-Myc and HDAC5 in PANC-1 and AsPC-1 cells. (**B**) Schematic diagrams of the truncations of GST-HDAC5. (**C**) Western blot analysis of full-length c-Myc protein in PANC-1 whole-cell lysate pulled down by GST or GST-HDAC5 recombinant proteins. Arrows indicate expected bands. (**D**) Co-IP detection of acetylated lysine on c-Myc in HDAC5-deficient PANC-1 and AsPC-1 cells. (**E**) Co-IP detection of acetylated lysine on c-Myc in PANC-1 and AsPC-1 cells overexpressing plasmids as indicated. (**F** and **G**) Co-IP analysis of c-Myc ubiquitination levels in HDAC5-deficient (**F**) or HDAC5-overexpressing (**G**) PANC-1 cells. (**H**) A hypothetical model depicting that HDAC5 deacetylates c-Myc, promoting its ubiquitination and degradation.

Observations indicated that c-Myc acetylation enhances its protein stability (14, 32, 33). Since HDAC5 promotes c-Myc protein degradation in an enzyme-dependent manner, we aimed to investigate whether HDAC5 regulates c-Myc protein acetylation. Reciprocal co-IP indicated the protein-protein interaction between HDAC5 and c-Myc at the endogenous level in the cell lines examined (Figure 5A). Glutathione-*S*-transferase (GST) pulldown assay indicated that the deacetylase (DAC) domain of HDAC5 is responsible for the interaction with c-Myc (Figure 5, B and C). HDAC5 knockdown resulted in a marked increase in endogenous c-Myc acetylation levels in both PANC-1 and AsPC-1 cells (Figure 5D). Consistent with this observation, overexpression of WT-HDAC5 reduced c-Myc acetylation, whereas the HDAC5 (H833A) mutant had no such effect (Figure 5E). More importantly, when HDAC5 status was manipulated, the ubiquitination level of c-Myc displayed an inverse trend relative to its acetylation level. Specifically, HDAC5 knockdown led to a reduction in c-Myc ubiquitination, whereas overexpression of WT-HDAC5 enhanced c-Myc ubiquitination (Figure 5, F and G, and Supplemental Figure 7, D and E). Collectively, our findings indicate that HDAC5 promotes c-Myc ubiquitination and subsequent degradation by mediating c-Myc deacetylation (Figure 5H).

### HDAC5 loss disrupts the acetylation-ubiquitination homeostasis at K148 of c-Myc.

**Figure 6:**
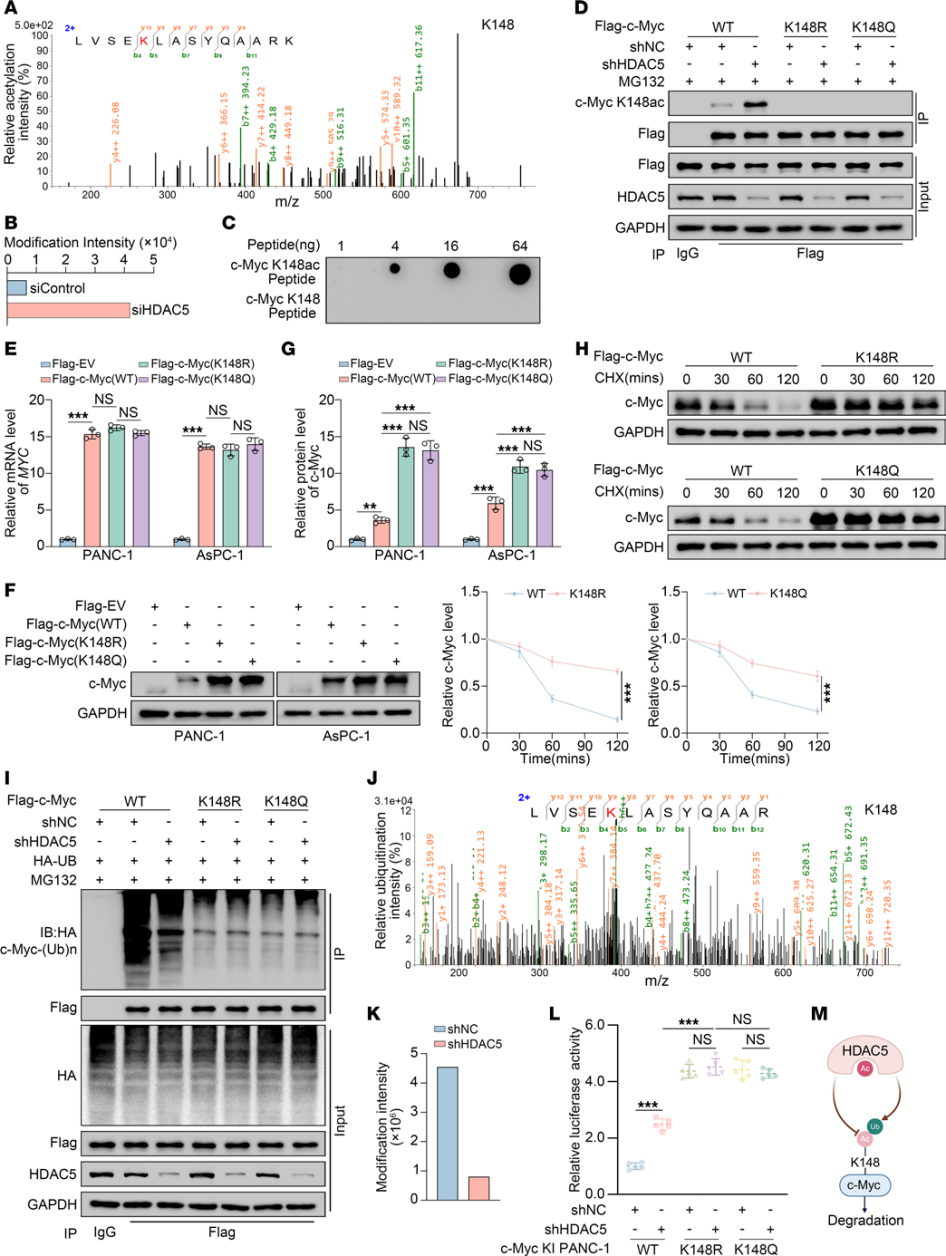
HDAC5 loss disrupts the acetylation-ubiquitination homeostasis at lysine 148 of c-Myc. (**A**) Illustration of c-Myc acetylation at K148 identified by mass spectrometry. (**B**) Mass spectrometry quantification of c-Myc-K148ac intensity in siControl vs. siHDAC5 groups. si, siRNA. (**C**) Different peptides dissolved in double-distilled H_2_O were applied to the nitrocellulose membrane, followed by immunoblotting with anti–c-Myc-K148ac antibody. (**D**) Co-IP detection of c-Myc-K148ac in PANC-1 cells expressing c-Myc (WT/K148R/K148Q) and treated with shNC or shHDAC5. (**E**–**G**) PANC-1 and AsPC-1 cells were transfected with equal amounts of c-Myc (WT/K148R/K148Q) plasmids for 48 hours. Afterward, cells were harvested for RT-qPCR analysis (**E**) and Western blot analysis (**F**), followed by protein quantification analysis (**G**) (*n* = 3). (**H**) Western blot analysis was conducted to evaluate c-Myc protein levels and normalized protein intensity in PANC-1 cells transfected with equal amounts of c-Myc (WT/K148R/K148Q) plasmids after treatment with 50 μg/mL CHX for the indicated durations (*n* = 3). (**I**) Co-IP detection of c-Myc ubiquitination in PANC-1 cells expressing c-Myc (WT/K148R/K148Q) and treated with shNC or shHDAC5. (**J**) Illustration of c-Myc ubiquitination at K148 identified by mass spectrometry. (**K**) A bar graph showing the intensities of c-Myc ubiquitination at K148 identified by mass spectrometry in shNC and shHDAC5 groups. (**L**) Luciferase reporter activities of c-Myc were assessed in PANC-1 cells infected with shHDAC5 and knockin c-Myc (WT/K148R/K148Q) (*n* = 5). (**M**) A hypothetical model illustrating how HDAC5 regulates c-Myc degradation through the competition between K148 acetylation and ubiquitination. All data are presented as the mean ± SD. Statistical significance was determined by 1-way ANOVA (**E** and **G**) or 2-way ANOVA (**H** and **L**), followed by Tukey’s multiple comparisons test. **P < 0.01, ***P < 0.001.

To identify the specific lysine residues involved in HDAC5-mediated deacetylation of c-Myc, we performed quantitative acetylation mass spectrometry analysis in control and HDAC5-knockdown PANC-1 cells. The results revealed a marked increase in the intensity of acetylation at K148 of c-Myc following HDAC5 knockdown, with an intensity ratio of 41,902.85 to 6,700.08 (Figure 6, A and B). To verify this, we generated acetylation-resistant (K148R) and acetylation-mimicking (K148Q) mutants by substituting the lysine (K) at position 148 of c-Myc with arginine (R) and glutamine (Q) (Supplemental Figure 8A). Co-IP analysis revealed that HDAC5 knockdown markedly increased the acetylation level of c-Myc (WT), while no changes were observed in c-Myc (K148R) or c-Myc (K148Q) (Supplemental Figure 8B). We further developed a specific antibody targeting K148-acetylated c-Myc (Figure 6C and Supplemental Figure 8, C–E). Co-IP analysis confirmed that HDAC5 knockdown substantially increased acetylation of c-Myc (WT) at K148, while the antibody failed to recognize the specific substrate in the K148R and K148Q mutants (Figure 6D).

To examine the selective role of HDAC5 in regulating c-Myc K148 acetylation in pancreatic cancer cells, we systematically depleted or pharmacologically inhibited class I HDACs (HDAC1, HDAC2, HDAC3), class IIa HDACs (HDAC4, HDAC7), and representative sirtuin family members (SIRT1, SIRT2) in PANC-1 cells (Supplemental Figure 9, A–L). Only HDAC5 depletion markedly elevated both c-Myc K148 acetylation and protein levels, whereas single perturbation of other HDACs or sirtuin family members had negligible effects (Supplemental Figure 9, A–L). Notably, treatment with the class I HDAC inhibitor MS-275 enhanced c-Myc K148 acetylation and protein expression in the HDAC5-proficient background, whereas it failed to do so in the HDAC5-knockdown setting (Supplemental Figure 9, B and H). This is consistent with prior reports indicating that HDAC5 can function as a “reader” of acetylated lysines, recruiting enzymatically active cofactors, including class I HDACs (HDAC1, HDAC2, HDAC3), to mediate deacetylation (17, 19). Furthermore, given reports that SIRT2 modulates c-Myc K148 acetylation in neuronal cells, we assessed the effects of SIRT2 knockdown alone or combined with HDAC5 depletion in PANC-1 and AsPC-1 cells. The results demonstrated that SIRT2 is dispensable for c-Myc K148 acetylation in pancreatic cancer cells, likely reflecting differences in cellular differentiation between pancreatic and neuronal lineages (Supplemental Figure 9, M–P). Importantly, selective inhibition of HDAC5 using LMK-235 alone robustly upregulated c-Myc K148 acetylation and protein levels (Supplemental Figure 9, Q and R). Collectively, these findings underscore HDAC5 as a pivotal regulator of c-Myc deacetylation at K148.

To further investigate how acetylation of c-Myc at K148 affects its ubiquitination and protein stability, we transfected equal amounts of c-Myc (WT), c-Myc (K148R), and c-Myc (K148Q) plasmids into PANC-1 and AsPC-1 cells. The results showed that the protein levels of c-Myc (K148R) and c-Myc (K148Q) were markedly higher than those of c-Myc (WT), with no notable difference observed between the 2 mutants (Figure 6, E–G). Further analysis revealed that the protein half-life of the c-Myc (K148R) and c-Myc (K148Q) mutants was substantially prolonged compared with that of c-Myc (WT) (Figure 6H). More interestingly, both c-Myc (K148R) and c-Myc (K148Q) exhibited markedly reduced ubiquitination levels compared with c-Myc (WT), and these levels were also unaffected by HDAC5 knockdown (Figure 6I). Based on these findings, we propose that HDAC5-mediated deacetylation of c-Myc at K148 likely affects ubiquitination at the same site, K148.

To validate this hypothesis, we performed quantitative ubiquitination modification mass spectrometry analysis in control and HDAC5transiently knocked down PANC-1 cells. As we proposed, HDAC5 knockdown also specifically reduced the ubiquitination at K148 of c-Myc, with a ubiquitination intensity ratio of 4,543,100 to 804,940 (Figure 6, J and K). Consistently, dual-luciferase reporter assay demonstrated that HDAC5 knockdown significantly enhanced the transcriptional activity of c-Myc (WT), while having no notable effect on the activity of the c-Myc (K148R) and c-Myc (K148Q) mutants, and both mutants exhibited even stronger luciferase activity compared with WT c-Myc following HDAC5 knockdown (Figure 6L). Collectively, our data suggest that HDAC5 promotes ubiquitination at K148 of c-Myc by mediating deacetylation at the same residue. Loss of HDAC5 leads to hyperacetylation of c-Myc at K148, disrupting the homeostasis of acetylation-ubiquitination at this site (dominant acetylation, diminished ubiquitination), which in turn enhances the protein stability and transcriptional activity of c-Myc (Figure 6M).

### HDAC5-mediated c-Myc deacetylation facilitates NEDD4-mediated ubiquitination at K148 of c-Myc.

**Figure 7:**
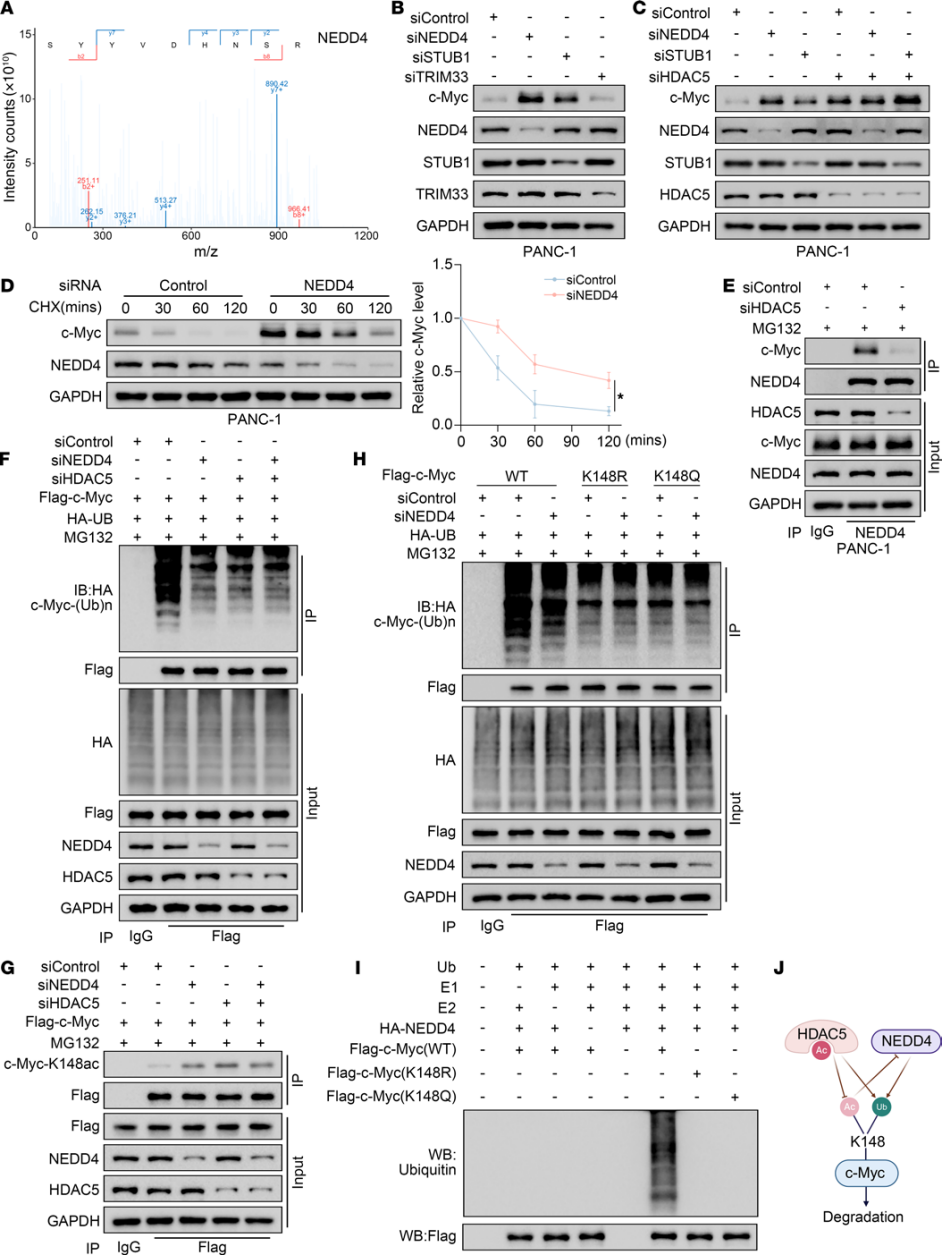
HDAC5-mediated c-Myc deacetylation facilitates NEDD4-mediated ubiquitination at K148 of c-Myc. (**A**) Mass spectrometry identified NEDD4 peptide fragments coprecipitated with c-Myc antibody, showing reduced signals upon HDAC5 knockdown. (**B** and **C**) Western blot analysis of PANC-1 cells infected with indicated siRNAs for 48 hours. (**D**) Western blot analysis and quantification of c-Myc protein stability in control or NEDD4-knockdown PANC-1 cells after CHX (50 μg/mL) treatment. All data are presented as the mean ± SD (*n* = 3). Statistical significance was determined by 2-way ANOVA followed by Tukey’s multiple comparisons test. *P < 0.05. (**E**) Endogenous co-IP analysis of the NEDD4/c-Myc interaction in PANC-1 cells transfected with indicated siRNAs. (**F** and **G**) Co-IP detection of c-Myc ubiquitination (**F**) and c-Myc-K148ac (**G**) in PANC-1 cells expressing c-Myc-WT and treated with siHDAC5, siNEDD4, or both. (**H**) Co-IP detection of c-Myc ubiquitination in PANC-1 cells expressing c-Myc (WT/K148R/K148Q) and treated with siControl or siNEDD4. (**I**) In vitro ubiquitination assay evaluating NEDD4 regulation of c-Myc (WT/K148R/K148Q) ubiquitination at K148. (**J**) A hypothetical model illustrating how HDAC5 and NEDD4 competitively modify the acetylation and ubiquitination of c-Myc at the K148 site, collaboratively promoting c-Myc protein degradation.

To identify the specific E3 ligase involved in HDAC5-mediated degradation of c-Myc, we performed proteomic analysis of c-Myc co-IP samples from control and HDAC5-knockdown PANC-1 cells. This revealed 3 E3 ligases — NEDD4, tripartite motif containing 33 (TRIM33), and STIP1 homology and U-Box containing protein 1 (STUB1), whose binding to c-Myc was markedly reduced after HDAC5 knockdown (Figure 7A and Supplemental Figure 10, A and B). Western blot analysis revealed that knockdown of NEDD4 or STUB1 markedly increased c-Myc protein levels, whereas knockdown of TRIM33 had no discernible effect on c-Myc expression (Figure 7B and Supplemental Figure 10, C–F). Further analysis revealed that in STUB1-knockdown cells, HDAC5 depletion further enhanced c-Myc expression. In contrast, in NEDD4-knockdown cells, HDAC5 depletion failed to elevate c-Myc levels (Figure 7C and Supplemental Figure 10G), indicating that HDAC5 and NEDD4 may regulate c-Myc protein levels via a shared mechanism.

Reciprocal co-IP confirmed the interaction between NEDD4 and c-Myc (Supplemental Figure 11, A and B). Ectopic overexpression of NEDD4 downregulated c-Myc protein expression, and this effect could be rescued by the treatment of MG132 (Supplemental Figure 11, C and D). Knockdown of NEDD4 prolonged the half-life of c-Myc protein to a similar extent as HDAC5 knockdown (Figure 7D and Supplemental Figure 11, E and F). Notably, NEDD4 and c-Myc interaction was markedly attenuated following HDAC5 knockdown (Figure 7E and Supplemental Figure 11G). Additionally, overexpressing either HDAC5 or NEDD4 alone, or both together, resulted in a similar inhibitory effect on c-Myc protein levels, which was reversed by the treatment of MG132 (Supplemental Figure 11H). We also observed a consistent pattern in co-IP assays. Single knockdown of either HDAC5 or NEDD4 led to a similar reduction in c-Myc ubiquitination, and dual knockdown of both genes could not further decrease c-Myc ubiquitination (Figure 7F). This was in direct contrast with the changes in acetylation at K148 of c-Myc (Figure 7G and Supplemental Figure 11I). More importantly, knockdown of NEDD4 specifically reduced the ubiquitination of c-Myc (WT) but had no effect on the ubiquitination levels of c-Myc (K148R) or c-Myc (K148Q) (Figure 7H), indicating NEDD4 specifically mediates the ubiquitination at K148 of c-Myc. This was further verified by the in vitro ubiquitination assay, which showed that NEDD4 mediated the ubiquitination of c-Myc (WT) but not c-Myc (K148R) or c-Myc (K148Q) (Figure 7I). In conclusion, these data indicate that NEDD4 specifically mediates the ubiquitination at K148 of c-Myc, promoting c-Myc degradation, while HDAC5 loss–mediated hyperacetylation at K148 of c-Myc dampens this process (Figure 7J).

### Pharmacological or genetic inhibition of c-Myc overcomes the resistance to KRAS inhibitor induced by HDAC5 loss.

**Figure 8:**
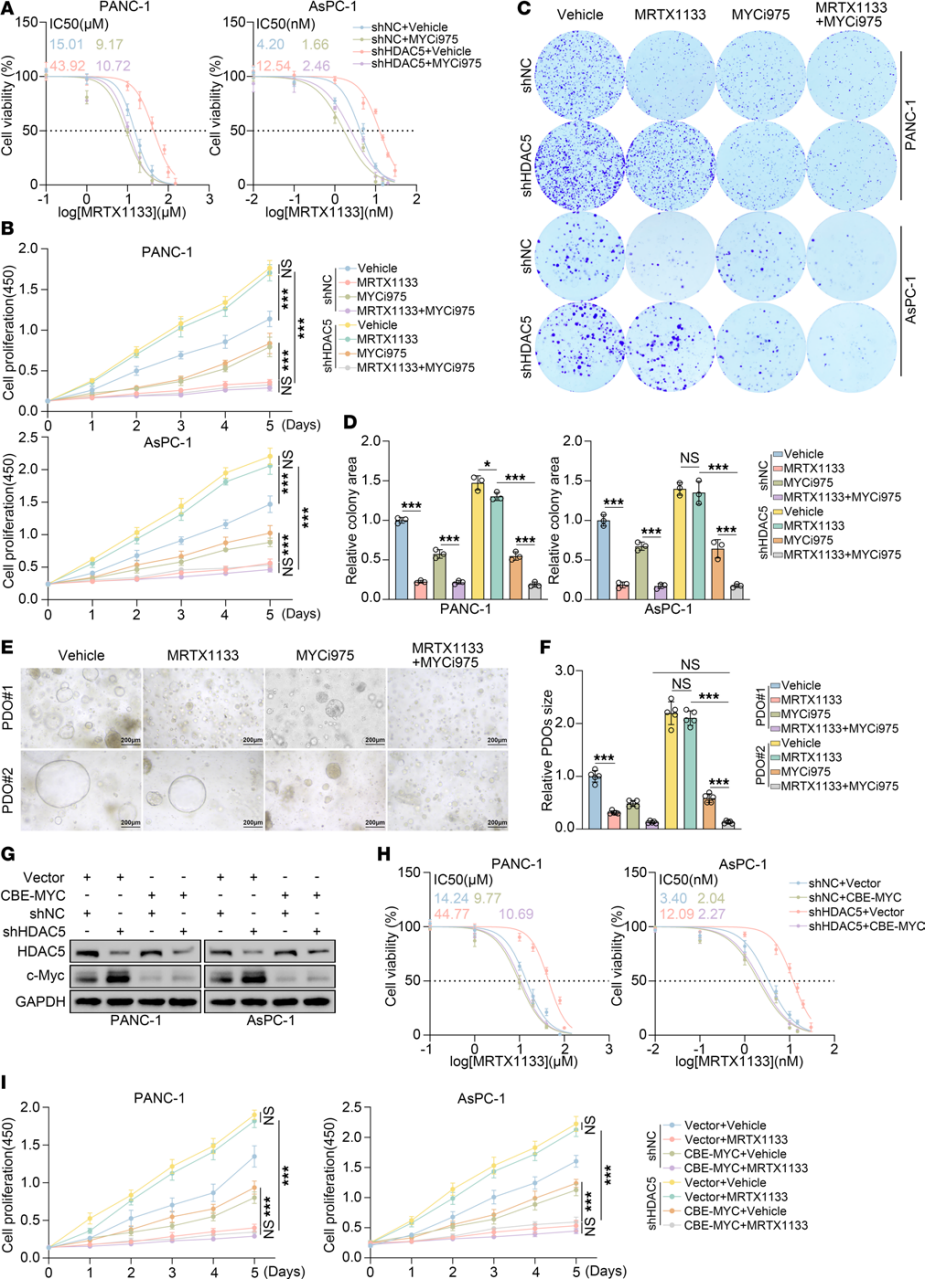
Pharmacological or genetic inhibition of c-Myc overcomes the resistance to KRAS inhibitor induced by HDAC5 loss in vitro. (**A**) IC_50_ values of MRTX1133 in PANC-1 and AsPC-1 cells expressing indicated plasmids for 48 hours, with or without MYCi975 (10 μM), measured by CCK8 assay. (**B**) Cell viability of PANC-1 and AsPC-1 cells transfected with the indicated plasmids and treated with DMSO, MRTX1133, MYCi975 (10 μM), or the combination, assessed by CCK8 assay. (**C** and **D**) PANC-1 and AsPC-1 cells were infected with the indicated shRNAs. At 48 hours postinfection, the cells were treated with vehicle (DMSO), MRTX1133 (10 μM for PANC-1; 5 nM for AsPC-1), MYCi975 (10 μM), or the combination. Colony formation assays were then performed. The resulting colonies were imaged and quantified using ImageJ (NIH) (**D**) (*n* = 3). (**E** and **F**) Representative images and size quantification of PDOs treated with DMSO, MRTX1133 (1 μM), MYCi975 (4 μM), or their combination. Scale bar = 200 μm (*n* = 5). (**G**) Western blot validation of MYC gene editing efficiency using a CBE in PANC-1 and AsPC-1 cells. (**H**) IC_50_ values of MRTX1133 under indicated conditions, assessed by CCK8 assay. (**I**) Cell viability of PANC-1 and AsPC-1 cells transfected with vector or CBE-MYC and treated with DMSO or MRTX1133 (PANC-1: 10 μM; AsPC-1: 5 nM) (*n* = 3). All data are presented as the mean ± SD. Statistical significance was determined by 2-way ANOVA followed by Tukey’s multiple comparisons test (**B**, **D**, **F**, and **I**). *P < 0.05, ***P < 0.001.

MYCi975 exerts its antitumor effects through a dual regulatory mechanism by directly targeting Myc protein to disrupt Myc/MAX dimer formation, thereby blocking Myc-dependent transcriptional activation while enhancing Myc degradation via the ubiquitin-proteasome pathway (34). Through Western blot, dual-luciferase reporter assay, ChIP-qPCR, and RT-qPCR, we demonstrated that MYCi975 inhibited the upregulation of representative downstream genes in the MAPK signaling pathway induced by HDAC5 knockdown in HDAC5-deficient cells (Supplemental Figure 12). The drug sensitivity analysis (IC_50_) revealed that MYCi975 reversed the resistance of HDAC5-knockdown PANC-1 and AsPC-1 cells to MRTX1133 (Figure 8A). 3D cell culture experiments further confirmed that MYCi975 effectively reversed the resistance of the cell lines examined and that combination therapy with MRTX1133 significantly inhibited cell proliferation (Supplemental Figure 13A). This finding was further validated through CCK8 assays and colony formation (Figure 8, B–D). In PDOs, MYCi975 also significantly sensitized HDAC5-low–expressing organoids to the treatment of MRTX1133 (Figure 8, E and F). Additionally, IHC analysis revealed that in HDAC5-low–expressing organoids, both the protein level of c-Myc and the acetylation level at the K148 site were significantly elevated (Supplemental Figure 13, B and C), whereas the combined treatment with MYCi975 and MRTX1133 significantly reduced the acetylation level of c-Myc at K148 in HDAC5-low–expressing organoids and decreased the expression of Ki67 (Supplemental Figure 13, B and C). 10058-F4 and MYCMI-6 act as MYC inhibitors, attenuating MYC-driven transcription by disrupting its essential dimerization with MAX while preserving c-Myc protein levels. We coadministered 10058-F4 (Supplemental Figure 14, A–D) or MYCMI-6 (Supplemental Figure 14, E–H) with MRTX1133 and evaluated their effects using IC_50_ measurements, colony formation assays, and CCK8 proliferation assays. Both compounds markedly mitigated MRTX1133 resistance in HDAC5-deficient cells, though their effect was less pronounced than that of MYCi975, which almost completely reversed the resistance. Given that HDAC5 loss elevates c-Myc acetylation, a key driver of intrinsic KRAS inhibitor resistance, we further investigated cotreatment with the P300/CBP inhibitor A-485 or the PCAF inhibitor L-45. Coadministration of these inhibitors partially relieved the MRTX1133 intrinsic resistance elicited by HDAC5 deficiency (Supplemental Figure 15). These findings indicate that pharmacological suppression of MYC in combination therapy can effectively counteract the intrinsic KRAS inhibitor resistance driven by HDAC5 deficiency.

CDS-NG-A3A-BE4max is a next-generation hyperactive cytosine base editor (hyCBE) with an expanded targeting range and markedly enhanced C-to-T editing efficiency while maintaining low off-target effects and minimal cytotoxicity. It is well suited for applications in gene therapy, disease modeling, and precise editing at complex genomic loci (35). Furthermore, studies have demonstrated that this editor can efficiently introduce stop codons, effectively silencing the proto-oncogene *MYC* (Supplemental Figure 16A) (36). We successfully silenced the *MYC* gene in HDAC5-deficient PANC-1 and AsPC-1 cells using this base editor, and the efficiency of *MYC* gene silencing was validated by Western blot and RT-qPCR (Figure 8G and Supplemental Figure 16B). IC_50_ demonstrated that silencing the *MYC* gene using this base editor reversed the resistance of HDAC5-knockdown PANC-1 and AsPC-1 cells to MRTX1133 (Figure 8H). CCK8 assays confirmed that silencing *MYC* via this base editor effectively reversed cell resistance, and combination treatment with MRTX1133 significantly inhibited cell proliferation (Figure 8I).

**Figure 9:**
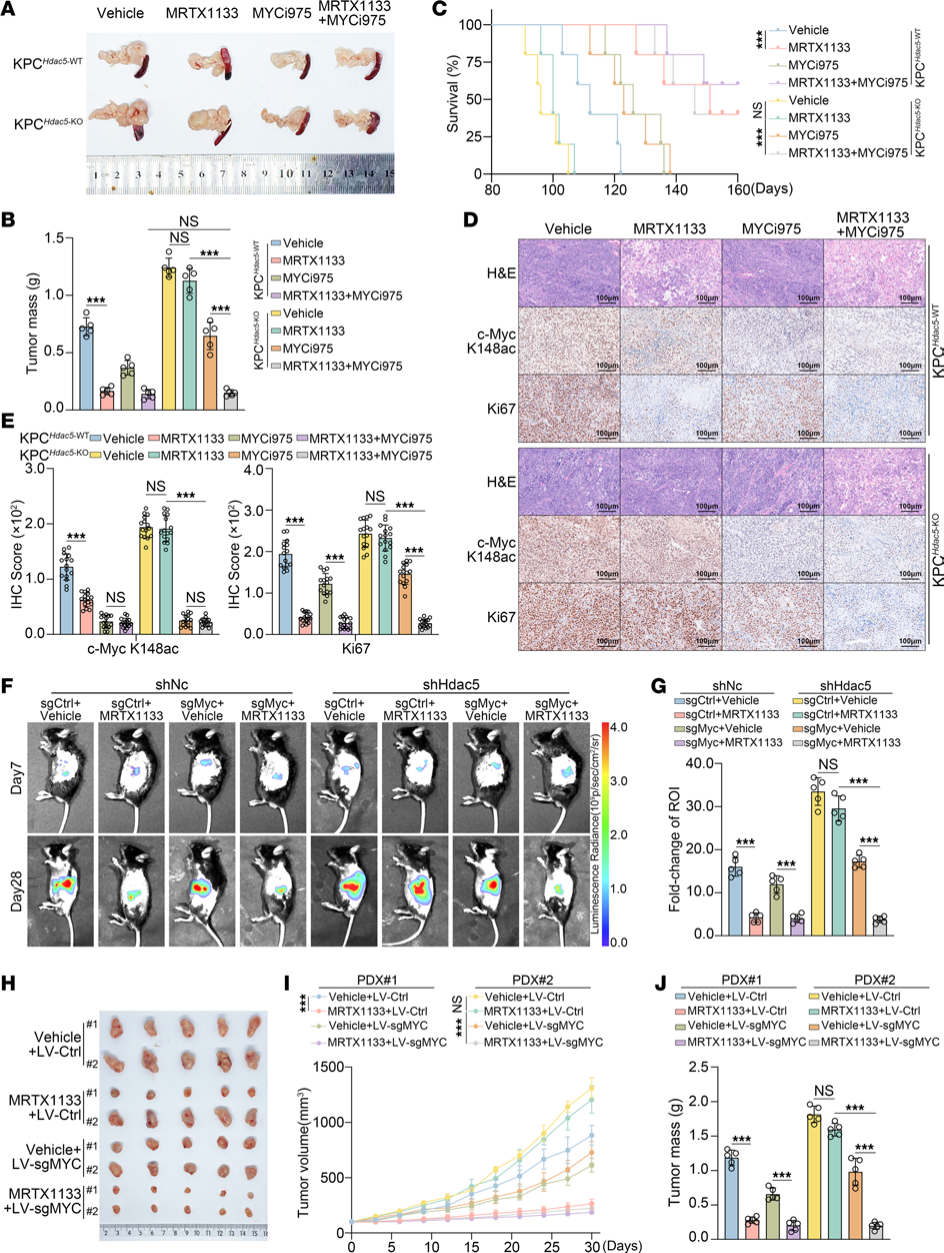
Pharmacological or genetic inhibition of c-Myc overcomes the resistance to KRAS inhibitor induced by HDAC5 loss in vivo. (**A**) Representative macroscopic images of pancreatic tumors from KPC^Hdac5-WT^ and KPC^Hdac5-KO^ mice after sacrifice. (**B**) Tumor weight analysis in KPC mice treated with vehicle, MRTX1133 (30 mg/kg, i.p. bid), MYCi975 (50 mg/kg, i.p. bid), or their combination (*n* = 5). (**C**) Kaplan-Meier survival curves with log-rank test (*n* = 5). ***P < 0.001. (**D** and **E**) Representative IHC images of tumors from treated mice and quantified IHC scores (**E**). Scale bars = 100 μm. *n* = 5 biologically independent repeats and 3 independent quantifications. (**F** and **G**) Representative bioluminescence images and quantification (*n* = 5). (**H**) Macroscopic images of PDX tumors after 30 days of treatment. (**I** and **J**) Tumor growth curves (**I**) and final tumor weights (**J**) in PDX models treated with Vehicle + LV-Control, MRTX1133 (30 mg/kg, i.p. bid) + LV-Control, Vehicle + LV-sgMYC (50 μL lentivirus, s.c., weekly), or MRTX1133 + LV-sgMYC. All data are presented as the mean ± SD. Statistical significance was determined by 2-way ANOVA followed by Tukey’s multiple comparisons test (**B**, **E**, **G**, **I**, and **J**). ***P < 0.001.

We further validated the findings in vivo utilizing the KPC^Hdac5-WT^ and KPC^Hdac5-KO^ mouse models (Supplemental Figure 17A). We found that MYCi975 improved the sensitivity of KPC^Hdac5-KO^ mice to MRTX1133. Combined treatment with MRTX1133 significantly reduced tumor burden and prolonged survival in the KPC^Hdac5-KO^ mice (Figure 9, A–C). IHC analysis further demonstrated that the combination of MYCi975 and MRTX1133 markedly reduced the c-Myc K148 acetylation levels in tumors of KPC^Hdac5-KO^ mice and inhibited the expression of Ki67 (Figure 9, D and E, and Supplemental Figure 17, B and C). Furthermore, in vivo, combining MYCMI-6 with MRTX1133 markedly increased the sensitivity of KPC^Hdac5-KO^ mice to MRTX1133 (Supplemental Figure 17, D–G).

We established Hdac5-knockdown KPC-Luc cells in vitro and subsequently achieved stable *Myc* gene knockout in this model (Supplemental Figure 18A). Using this cell line, we further developed a C57BL/6 pancreatic cancer orthotopic model (Supplemental Figure 18B). The study demonstrated that silencing *Myc* enhanced the treatment sensitivity of Hdac5-low–expressing orthotopic tumors to MRTX1133. In vivo imaging analysis further revealed that combination treatment with MRTX1133 significantly reduced the fluorescence intensity of Hdac5-low–expressing orthotopic tumors (Figure 9, F and G). We further established a PDX mouse model of pancreatic cancer with subcutaneous transplantation. We constructed a lentiviral vector encoding CRISPR-Cas9/sgMYC and delivered it into the PDX mouse model to achieve in vivo gene editing. Experimental results demonstrated that lentivirus-mediated sgMYC delivery markedly enhanced the sensitivity of HDAC5-low PDX models to MRTX1133. Combined MRTX1133 and *MYC*-knockdown treatment suppressed tumor growth, as further confirmed by IHC analysis (Figure 9, H–J, and Supplemental Figure 18, C–F).

To evaluate the generality of MYC coinhibition in overcoming HDAC5 deficiency–driven intrinsic resistance to KRAS inhibitors, we administered MYCi975 together with HRS-4642 in KPC^Hdac5-WT^ and KPC^Hdac5-KO^ mice and with GFH-375 in PDX models. The combination treatment significantly increased tumor responsiveness to both KRAS^G12D^ inhibitors (Supplemental Figures 19 and 20). In *KRAS^G12C^* mutant Mia PaCa-2 cells, combined *MYC* suppression likewise reversed HDAC5 deficiency–mediated resistance to sotorasib, a finding further substantiated through systematic in vitro and in vivo analyses (Supplemental Figures 21 and 22). In summary, our data indicated that pharmacological or genetic inhibition of c-Myc alleviated the intrinsic resistance to KRAS inhibitor induced by HDAC5 deficiency in multiple PDAC models.

## Discussion

Preclinical studies and early-phase clinical trials indicate that primary and acquired resistance to KRAS inhibitors frequently arises, potentially because of aberrant activation of compensatory signaling pathways (37). Studies have shown that, in resistant PDAC cell lines, organoids, and animal models, there is a substantial increase in the gene copy number of RTK/RAS pathways (such as *EGFR*, *MET*, *BRAF*, *ETV1*) and epithelial-mesenchymal transition–related (EMT-related) factors (such as *ZEB1*, *TWIST1*) (38). Moreover, the expression of EMT genes, *MYC* activity, and secondary mutations in *KRAS^G12D^* (such as the V9W variant) were identified as the key drivers of resistance (39). In addition, PDAC with gene alterations, such as *PTEN*, *NF1*, and *RB1*, may exhibit intrinsic resistance to MRTX1133 (5, 38), while upregulation of immune checkpoint markers (such as *CTLA4*, *LAG3*) and cellular heterogeneity further exacerbate resistance (7, 8, 37, 38). In our current study, we identify the gene alteration of *HDAC5* (HDAC5 deletion) as a driver of intrinsic resistance to MRTX1133 in *KRAS^G12D^*-mutant PDAC, via the activation of c-Myc transcriptional activity independent of the KRAS/MEK/ERK axis, resulting in sustained MAPK signaling and promoting cell survival despite KRAS inhibition. These findings reveal HDAC5’s role in rewiring KRAS signaling networks and provide a mechanistic basis for developing combination therapies to overcome KRAS inhibitor resistance in HDAC5 loss PDAC.

c-Myc acetylation represents a pivotal regulatory mechanism across multiple pathological contexts. In neuronal systems, SIRT2-mediated deacetylation of c-Myc at K148 modulates its conformational stability and subcellular localization, and regulates proapoptotic activity, suggesting SIRT2 as a potential therapeutic target in stroke (31). In cancer research, despite some discrepant findings, most observations indicated that acetylation acts as an activating modification that potentiates c-Myc function. In anaplastic thyroid carcinoma, KAT5 acetylates and stabilizes c-Myc, promoting proliferation, invasion, and EMT (40). In hepatocellular carcinoma (HCC), the PCK1/KAT5 axis facilitates c-Myc acetylation, activating c-Myc–mediated EMT to promote metastasis. In non–small cell lung cancer, acetate promotes c-Myc acetylation, stabilizing c-Myc and inducing PD-L1, glycolytic enzymes, and cell cycle regulators — thereby fostering immune evasion and supporting tumor growth (41). Conversely, in HCC, HDAC5 deacetylates c-MYC at K143/K157, enhancing transcription of CDK1, CDK4, and CDC25C to drive cell cycle progression (42). Collectively, these studies underscore c-Myc acetylation as a context-dependent regulatory node controlled by specific acetyltransferases and deacetylases. Targeting these modifying enzymes offers a promising strategy for modulating c-Myc activity in multiple diseases, providing a mechanistic rationale for precision therapeutic development.

The stability of the c-Myc protein is tightly regulated by the ubiquitin-proteasome system, where ubiquitination facilitates proteasomal degradation through a feedback mechanism that interacts with protein synthesis pathways. This process ensures the dynamic balance of c-Myc protein levels and fine-tunes its transcriptional activity (43, 44). Additionally, c-Myc stability plays a critical role in the KRAS pathway. Beyond the well-characterized ERK-dependent phosphorylation that controls c-Myc stability (12), emerging studies have revealed an additional layer of regulation through acetylation-dependent modulation of c-Myc ubiquitination and proteasomal turnover (14, 29, 32). However, the detailed molecular mechanisms underlying this regulation remain poorly characterized. Our study demonstrates a competitive relationship between acetylation and ubiquitination modifications specifically at the K148 site of c-Myc protein. HDAC5 collaborates with the ubiquitin E3 ligase NEDD4 to promote c-Myc K148 ubiquitination and degradation, while HDAC5 loss–mediated c-Myc K148 hyperacetylation impairs NEDD4-mediated ubiquitination, leading to an abnormal stability of c-Myc even under KRAS inhibition. This observation aligns with the classic “acetylation of c-Myc interference with ubiquitination” model (45, 46). Notably, our findings establish that the dynamic equilibrium between acetylation and ubiquitination at K148 serves as a molecular switch regulating c-Myc stability, providing a deeper mechanistic understanding of this regulatory phenomenon.

KRAS inhibitors, currently in clinical trials for multiline treatment of PDAC, represent a last resort for patients with advanced disease. However, the emergence of resistance to these agents poses a life-threatening challenge for patients with PDAC who have exhausted other treatment alternatives. Thus, constructing effective drug combination strategies is paramount for circumventing this treatment resistance. Studies have demonstrated that combining MRTX1133 with the ERBB inhibitor afatinib or the PI3Kα inhibitor BYL-719 enhances antitumor effects and reverses acquired resistance (5, 8). Furthermore, combining MRTX1133 with immune checkpoint inhibitors enhances efficacy by activating T cell–mediated immune responses (6, 7, 38). Additionally, intervention of YAP/TAZ signaling, the EGFR/HER family, the S6 pathway, and SHP2 further expands the potential for combination therapy (5). Despite c-Myc’s reputation as an undruggable target, we established viable therapeutic strategies, such as cofactor deprivation and gene editing. Our multiplatform validation in PDAC models reveals that c-Myc ablation circumvents the HDAC5 loss–induced intrinsic resistance to MRTX1133, restoring KRAS inhibitor vulnerability. Additionally, our study suggests that HDAC5 expression levels or c-Myc acetylation status could serve as biomarkers for predicting the efficacy of MRTX1133, providing a theoretical basis for personalized treatment in PDAC.

Several limitations in our study warrant consideration. First, most of our findings were obtained from PDAC cell lines, organoids, and murine models, which may incompletely reflect the heterogeneity and complex microenvironment of human pancreatic tumors. Second, although we demonstrate that the HDAC5/c-Myc/MAPK signaling axis is pivotal for intrinsic resistance to KRAS inhibitors, the potential involvement of other regulators — such as alternative E3 ligases, competing acetyltransferases, deubiquitinases, or HDAC5-independent pathways — remains undetermined. Third, the exact contribution of this mechanism to KRAS^G12D^-acquired resistance remains unresolved and requires further investigation to determine its effect on adaptive resistance. Finally, although cotargeting c-Myc holds potential to sensitize HDAC5-deficient PDAC to KRAS inhibitors, its translational applicability needs further validation in larger patient-derived cohorts and clinical settings.

In conclusion, this study elucidates the molecular mechanism by which HDAC5 deletion enhances the protein stability of the transcription factor c-Myc, continuously activates the MAPK signaling pathway, and drives intrinsic resistance to MRTX1133 in *KRAS^G12D^* mutant PDAC. We further confirm that the multifaceted inhibition of c-Myc can effectively reverse the resistance phenotype mediated by HDAC5 deletion, providing a strategy based on KRAS-MYC dual-node blockade to overcome resistance to KRAS-targeted therapy in HDAC5-deficient PDAC.

## Methods

### Sex as a biological variable.

Our study included both male and female animals, and comparable results were observed across sexes.

### Cell lines and cell culture.

The cell lines used in this study, including HEK293T (CL-0005), PANC-1 (CL-0184), AsPC-1 (CL-0027), and Mia PaCa-2 (CL-0627) were purchased from Procell Life Science & Technology Co., Ltd. The murine KPC-Luc cell line (NM-YD04-TG01) was purchased from Model Organisms Inc. All cell lines were authenticated by short tandem repeat profiling and routinely tested for mycoplasma contamination using the Lookout Mycoplasma PCR Detection Kit (Sigma-Aldrich). Cells were cultured in DMEM (Gibco) supplemented with 10% FBS (Gibco) at 37°C in a humidified incubator with 5% CO_2_.

### Animals.

Immunocompromised NOG mice (NOD.Cg*-Prkdc^scid^ Il2rg^tm1Sug^* /Jic) of 6 weeks of age were purchased from Vital River Laboratories (#408) and used for establishing PDX models. C57BL/6 mice at 4 weeks of age were purchased from Vital River Laboratories (#219) and used to establish a pancreatic cancer orthotopic model with KPC-Luc cells. KP [*Kras^tm1(LSL-G12D)^Trp53^tm1(LSL-R172H)^*, C001320] mice, *Hdac5*-KO (S-KO-02424) mice, and Tg(*Pdx1-Cre*) (C001408) mice were all purchased from Cyagen and interbred to generate KPC^Hdac5-KO^ mice. KPC [*Kras^tm1(LSL-G12D)^Trp53^tm1(LSL-R172H)^Pdx1*-Cre] mice were purchased from Cyagen (C001308). Four-week-old BALB/c nude mice purchased from Vital River Laboratories (#401) were used to establish a xenograft model with Mia PaCa-2 cells. All mice were housed in a specific pathogen–free facility at Tongji Medical College, Huazhong University of Science and Technology.

### Generation of c-Myc K148ac polyclonal antibody.

A polyclonal antibody specifically recognizing c-Myc K148 acetylation (K148ac) was custom-generated. Detailed descriptions can be found in Supplemental Methods.

### ChIP and ChIP-Seq analysis.

Ten million PANC-1 cells were cross-linked with 1% formaldehyde at room temperature for 10 minutes, quenched with 125 mM glycine, and subsequently harvested. For each condition, 2 independent biological replicates were prepared. The harvested cells were submitted to Active Motif China for ChIP-Seq. Detailed descriptions can be found in Supplemental Methods.

### RNA sequencing and bioinformatics analysis.

RNA sequencing was performed by Haplox Biotechnology. The sample cohort for RNA sequencing consisted of pancreatic tumors from KPC^Hdac5-WT^ and KPC^Hdac5-KO^ mice (*n* = 5 biological replicates per genotype) and AsPC-1 cells subjected to respective treatments (*n* = 3 biological replicates per group). Detailed descriptions can be found in Supplemental Methods.

### Statistics.

Statistical analyses were performed using GraphPad Prism 9.5. An unpaired 2-tailed Student’s *t* test was used for comparisons between 2 groups. For comparisons involving 3 or more groups, 1-way or 2-way ANOVA followed by Tukey’s post hoc test was applied. *P* values < 0.05 were considered statistically significant. Data were shown as mean ± SD.

### Study approval.

The human-derived tissues involved in this study were approved by the Ethics Committee of Tongji Medical College, Huazhong University of Science and Technology (Approval No. UHCT-IEC-SOP-016-03-01), and written informed consent was obtained from all patients. The animal experiments conducted in this study were approved by the Institutional Animal Care and Use Committee of Tongji Medical College, Huazhong University of Science and Technology (Approval No.: IACUC 2583).

### Data availability.

All source data values were provided in the Supporting Data Values file. The ChIP-Seq data have been deposited in the NCBI GEO database under accession number GSE309501, and the RNA-Seq data are available in the GEO database under accession numbers GSE175374, GSE293877, and GSE309499. Requests for further information, resources, and reagents should be directed to the lead contact. Additional methods are provided in Supplemental Methods.

## Author contributions

Y Zhou and TC were responsible for conceptualization. Y Zhou, TC, and HY designed the methodology. HY developed the software. TC performed the validation. Formal analysis was conducted by TC, HY, and Y Zhou. Investigation was carried out by TC, HY, KW, GQ, Y Zhao, XL, YL, TZ, JL, JZ, ZL, RW, BW, SG, and XJ. Resources were provided by Y Zhou, XJ, HW, TY, KW, and SG. Data curation was performed by Y Zhou, TC, and HY. The original draft of the manuscript was written by TC and HY, and all authors contributed to review and editing. Visualization was performed by TC and HY. Supervision was provided by KW, HW, and Y Zhou. Project administration was handled by Y Zhou, XJ, and HW. Funding was acquired by Y Zhou, HW, BW, SG and TY. The order of authorship was determined based on overall contributions and was approved by all authors.

## Funding support

- National Natural Science Foundation of China, including No. 82573059 and No. 82102794 (to Y Zhou), No. 82573316 and No. 82373331 (to HW), No. 82273209 (to BW).
- Hubei Provincial Natural Science Foundation (No. 2021CFB590 to SG).
- Key Research and Development Program of Hubei Province No. 2022BCA012 (to TY).

## Supplementary Material

Supplemental data

Unedited blot and gel images

Supporting data values

## Version 1 12/11/2025

In-Press Preview

## Version 2 02/02/2026

Electronic publication
