# Supplementary material for: HDAC5 deficiency induces intrinsic resistance to KRAS inhibition by disrupting c-Myc acetylation-ubiquitination homeostasis
Source: J Clin Invest. 2025 Dec 11;136(3):e195814. doi: 10.1172/JCI195814 (PMC12867140; doi:10.1172/JCI195814)
Supplement: Supplemental data [file jci-136-195814-s047.pdf]

Supplemental material includes Supplemental Methods, 22 Supplemental Figures and Supplemental Figure legends, 2 Supplemental Tables.

Supplemental Methods

Microbe Strains

Competent E. coli DH5α or BL21 (DE3) cells were transformed and cultured on LB agar plates supplemented with 100 mg/mL of Ampicillin at 37°C for plasmid selection.

Transfection of Plasmids

Transfection was performed following the manufacturer's protocols using either Lipofectamine 2000 (Thermo Fisher Scientific, Cat#11668030) or Neofect (Renjiu Bio Technology, Cat#TF20121201). Briefly, 2-5μl of transfection reagent was combined with 1μg plasmid DNA. Both components were independently diluted in Opti-MEM medium (Gibco, Cat#11058021), subsequently combined, and allowed to form complexes at room temperature for 5 minutes. The resulting DNA-lipid complexes were then administered to cultured cells.

RNA Interference

Control and gene-specific shRNA lentiviral constructs were obtained from Sigma-Aldrich. The packaging plasmids psPAX2 and PMD2.G were co-transfected with shRNA constructs into HEK293T cells using a lentiviral packaging system. Following a 24-hour incubation, the culture medium was refreshed, and viral supernatants were subsequently collected through filtration. The harvested viral particles were then employed to transduce cancer cells using 6μg/mL polybrene (Beyotime, Cat#C0351)

as a transduction enhancer. Target cells were collected 48 hours post-puromycin selection. All shRNA and siRNA sequences are detailed in Supplemental Table 1.

CRISPR/Cas9-Mediated Gene Knockout

The oligonucleotides targeting MYC were annealed and ligated into GV708 vectors (Genechem, U6-sgRNA-EF1a-Cas9-FLAG-CMV-EGFP-P2A-puro) through restriction enzyme-based subcloning. The sgRNA expression vectors were co-transfected with packaging plasmids pMDG.1 and psPAX2 into HEK293T cells using Lipofectamine 2000 transfection reagent, following the manufacturer's optimized protocol. The culture medium was refreshed 24 hours post-transfection, and the viral supernatants were collected at 72 hours. The viral particles were concentrated by ultracentrifugation at 80,000 × g for 2 hours at 4°C. Transduced cells expressing sgRNA were selected by puromycin treatment (Beyotime, Cat# ST551) at empirically determined concentrations for 5-7 days to establish stable cell lines. The specific sgRNA sequences used for CRISPR/Cas9-mediated MYC knockout are documented in Supplemental Table 1.

Transfection of CBE

The CDS-NG-A3A-BE4max plasmid and its associated sgMYC plasmid were kindly provided by Dr. Keshan Wang at the Tianjin Institute of Industrial Biotechnology. Cells were plated in 24-well plates and transfected with polyethylenimine (PEI; Polysciences, USA) following the manufacturer's instructions. A total of 600 ng CBE plasmid and 300 ng sgRNA expression plasmid were mixed with 50 µl Opti-MEM (Gibco, USA) and 2.7 µl PEI for transfection. After 24 hours, the medium was replaced with fresh

medium, and cells were selected using 5 μg/ml puromycin (Beyotime, Cat#ST551) for 3–5 days to generate stable cell lines.

High-throughput screening

PANC-1 and MIA PaCa-2 cells were cultured in DMEM supplemented with 10% FBS and digested into a single-cell suspension using 1× TrypZean Solution (Sigma-Aldrich, USA). Cells were then seeded at a density of 1,000 cells per well in a final volume of 100 μL per well in 96-well plates using a Multidrop®™ Combi Reagent Dispenser (Thermo Fisher Scientific). After overnight incubation at 37°C in a humidified atmosphere with 5% CO2, cells were treated with the compound library. Compounds were purchased from SelleckChem (#L8000) and dissolved either in DMSO or double-deionized water, with each drug applied at 10 μM. Cell viability was assessed after 48 hours using the CCK-8 assay.

Quantitative Real-Time PCR (RT-qPCR)

Total RNA was extracted using TRIzol reagent (Thermo Fisher Scientific, Cat#15596018). RNA concentration and purity were assessed with a NanoDrop 2000 spectrophotometer (Thermo Fisher Scientific). Reverse transcription was performed using the PrimeScript™ RT Reagent Kit (Takara Bio Inc, Cat#RR037A) according to the manufacturer's protocol. Real-time quantitative PCR was carried out using TB Green™ Fast qPCR Mix (Takara Bio Inc, Cat#RR430A) on an iCycler QTX detection system (Bio-Rad). Relative gene expression levels were determined using the $2^{-\Delta\Delta CT}$ method, with GAPDH as the endogenous control. Primer sequences are provided in Supplemental Table 1.

Co-Immunoprecipitation (Co-IP) and Western Blot Analysis

For co-immunoprecipitation (Co-IP), cells were collected and lysed in IP buffer (Beyotime, Cat#P0013) on ice for 30 minutes, followed by centrifugation at 12,000 rpm for 10 minutes at 4°C. The supernatant was then incubated overnight at 4°C with either the primary antibody or IgG, along with protein A/G agarose beads (Beyotime, Cat#P2055). The beads were washed at least six times with IP buffer on ice and subsequently boiled in loading buffer for Western blot analysis. Protein concentration of the supernatant was determined using the BCA protein quantification assay (Beyotime, Cat#P0012S). Equal amounts of protein were mixed with loading buffer (Beyotime, Cat#P0015), boiled for 10 minutes, and then separated via SDS-PAGE before being transferred onto a nitrocellulose membrane (Millipore, Cat#IPVH00005). The membrane was blocked with 5% milk powder in 1×TBST for 1 hour at room temperature, followed by overnight incubation with primary antibodies at 4°C. After three washes with 1×TBST, the membrane was incubated with HRP-conjugated secondary antibodies for 1 hour at room temperature. Protein bands were detected using ECL detection reagents (Meilunbio, Cat#MA0186) and visualized with the ChemiDoc XRS imaging system (Bio-Rad).

Generation of c-Myc K148ac Polyclonal Antibody

A polyclonal antibody specifically recognizing c-Myc K148 acetylation (K148ac) was custom-generated. Synthetic peptides corresponding to the acetylated (VSE(-Ac-K-)LASY) and unmodified (VSEKLASY) sequences were produced via solid-phase synthesis, purified by HPLC (>90% purity), and verified by mass spectrometry. The

acetylated peptide was covalently coupled to keyhole limpet hemocyanin (KLH) using m-maleimidobenzoyl-N-hydroxysuccinimide (MBS) to serve as the immunogen. Four 2–3-month-old rabbits were immunized subcutaneously at multiple dorsal sites with a primary injection of 1.6 mg peptide in Freund's complete adjuvant, followed by seven booster injections of 0.8 mg peptide in Freund's incomplete adjuvant on days 28, 42, 47, 54, 64, 74, and 84. Final bleeding was performed on day 94. Antisera were affinity-purified sequentially using acetylated and unmodified peptide-conjugated Sepharose 4B columns to isolate K148ac-specific antibodies. Antibody concentrations were determined by Bradford assay, and specificity was confirmed by Western blot, and dot blot, showing strong recognition of the acetylated peptide without cross-reactivity to the unmodified peptide or other common acetylated peptides (H3 K9ac/K9, H3 K27ac/K27, p53 K370ac/K370, p53 K382ac/K382). Purified antibodies were stored at -20°C in PBS (pH 7.0–7.2) containing 0.02% NaN_3 and 50% glycerol, avoiding repeated freeze-thaw cycles.

ChIP and ChIP-seq Analysis

Ten million PANC-1 cells were cross-linked with 1% formaldehyde at room temperature for 10 min, quenched with 125 mM glycine, and subsequently harvested. For each condition, two independent biological replicates were prepared. The harvested cells were submitted to Active Motif China (Shanghai, China) for ChIP–seq. Chromatin preparation, ChIP reactions, library construction, and data analysis were carried out by Active Motif. Briefly, chromatin was isolated using lysis buffer and fragmented by sonication. DNA was sheared to an average size of 200–500 bp using the EpiShear

probe sonicator (Active Motif, Cat#53051). Input genomic DNA was obtained by reverse cross-linking at 65 °C for 4 h, followed by sequential digestion with RNase A (Thermo Fisher) and Proteinase K (Thermo Fisher). DNA was purified with the QIAGEN PCR Purification Kit and quantified using a OneDrop spectrophotometer (Wins). Fragmented chromatin designated for immunoprecipitation was incubated overnight at 4 °C with 2 μg anti-c-Myc antibody (Abcam, Cat# ab32072). Twenty-five microliters of rProtein A Magarose Beads (Smart-lifesciences) were added and incubated for 2 h at 4 °C. Immune complexes were washed, eluted from the beads with SDS buffer, and reverse cross-linked overnight at 65 °C. The resulting chromatin was treated with RNase A and Proteinase K. ChIP DNA was purified using the QIAGEN PCR Purification Kit and quantified with a Qubit fluorometer (Thermo Fisher). Sequencing libraries were generated from ChIP and input DNAs using the Hieff NGS® DNA Library Prep Kit (Yeasen). Libraries were multiplexed and sequenced on an Illumina NovaSeq 6000 platform using the NovaSeq 6000 S4 Reagent Kit v1.5 (300 cycles).

Raw sequencing reads in FASTQ format were processed with fastp (v1.0.1) to remove adapter sequences, filter out low-quality reads, and trim low-quality bases, yielding high-quality clean reads for downstream analysis. The clean reads were aligned to the UCSC hg38 reference genome (hg38. analysisSet.fa) using BWA-MEM2 (v2.3). After alignment, PCR duplicates were marked and removed using Picard MarkDuplicates (v3.4.0). Peak calling was performed with MACS3 (v3.0.3) using a q-value threshold of 0.05. Differential binding analysis between experimental groups was

conducted using the DiffBind (v3.16.0) R package, which normalized read counts across a consensus peak set and identified differential sites with an FDR-adjusted p-value < 0.05. Finally, data visualization, including heatmaps and signal profile plots across peaks, was generated using deepTools (v3.5.6).

ChIP-qPCR

ChIP assays were conducted in pancreatic cancer cells following the manufacturer's instructions, using the Pierce Magnetic ChIP Kit (Thermo Fisher Scientific, Cat#26157, www.thermofisher.cn/search/results?query=26157&persona=DocSupport&refinementAction=true&personaClicked=true). Protein G magnetic beads and a specific antibody were utilized for chromatin immunoprecipitation, with approximately 5μg of antibody used for 25μg of chromatin. Additionally, normal rabbit IgG was used as a negative control. The PCR Kit (Promega) was then applied to amplify the purified input and immunoprecipitated DNA. Following purification, qPCR was performed to analyze the promoters of the DNA samples. The primer sequences and antibodies used in the experiment are listed in Supplemental Table 1.

Dual Luciferase Reporter Assay

To assess the transcriptional activity of MYC, MAP4K4, MAPK7, MRAS, and PAK1, PANC-1 cells were transfected with luciferase reporter constructs containing their promoters, which were cloned into the pGL3-basic vector (Promega), along with a Renilla luciferase reporter (phRL-TK, Genechem). Transcriptional activity was measured using the Dual-Luciferase Reporter Assay System (Promega, Cat#E1910), normalizing firefly luciferase activity to Renilla luciferase activity for accurate

quantification of promoter activity.

Generation of Knockin Cell Lines

The guide RNAs for generating MYC K148R and MYC K148Q cell lines were cloned into the pSpCas9-2A-Puro (PX459) vector. PANC-1 cells were co-transfected with PX459 vectors and pMD19-T plasmids containing the K148R/K148Q point mutation, which served as the template for homology-directed repair. Following selection with 2 mg/mL puromycin for 36 hours, the cells were plated into 96-well plates to establish monoclonal cell populations. The knockin efficiency was confirmed by genomic sequencing and immunoblotting. All gRNAs used in this study are listed in Supplemental Table 1.

RNA Sequencing and Bioinformatics Analysis

RNA sequencing was performed by Haplox Biotechnology (Jiangxi, China). The sample cohort for RNA sequencing consisted of pancreatic tumors from KPC*Hdac5*-WT/KO mice (n=5 biological replicates per genotype) and AsCP-1 cells subjected to respective treatments (n=3 biological replicates per group). Approximately 30 mg of tissue, ground in liquid nitrogen, was subjected to total RNA extraction using TRIzol™ Reagent (Thermo, #15596018). RNA purity was assessed with a NanoDrop™ One (Thermo, #ND-ONE-W), and concentration was measured using the Qubit™ RNA BR Assay Kit (Invitrogen, #Q10210) on a Qubit™ 3 Fluorometer (Thermo, #Qubit 3.0). RNA integrity was evaluated with RNA Screen Tape (Agilent, #5067-5576) and RNA Screen Tape Sample Buffer (Agilent, #5067-5577) on an Agilent 4200 TapeStation System (Agilent, #Agilent 4200). For mRNA library preparation, 0.1–1 µg of total RNA

was used for poly(A) RNA isolation with the NEBNext® Poly(A) mRNA Magnetic Isolation Module (NEB, #E7490L), followed by library construction using the NEBNext® Ultra™ II mRNA Library Prep Kit for Illumina® (NEB, #E7770L). Library concentration was determined using the Qubit™ dsDNA HS Assay Kit (Invitrogen, #Q32851) on the Qubit™ 3 Fluorometer (Thermo, #Qubit 3.0), and fragment size distribution was assessed using D1000 Screen Tape (Agilent, #5067-5582) with D1000 reagents (Agilent, #5067-5583). Library molarity was precisely quantified using the KAPA Library Quant Kit (Illumina, #kk4824) on a QuantStudio™ 3 Real-Time PCR System (Thermo, #A28572). Sequencing was performed on a NovaSeq 6000 platform using the NovaSeq S4 Reagent Kit according to the manufacturer's instructions.

Raw sequencing reads in FASTQ format were processed using fastp (v1.0.1) to remove adapter sequences, filter out low-quality reads, and trim low-quality bases, yielding high-quality clean reads for downstream analysis. The clean reads were aligned to the human UCSC hg38 reference genome using transcript annotations from the UCSC RefSeq database (hg38.ncbiRefSeq.gtf). For mouse data, alignment was performed against the GRCm39 reference genome with GENCODE M28 annotations. Alignment was carried out using Subread (v2.1.1), and gene-level read counts were quantified with featureCounts (v2.1.1, part of the Subread package). For samples with biological replicates, differential gene expression analysis was performed using DESeq2 (v1.46.0), which models read counts using a negative binomial distribution and applies the Benjamini–Hochberg procedure to control the false discovery rate

(FDR). Genes with an absolute $\log_2$ fold change greater than 1 and an FDR-adjusted p-value < 0.05 were considered differentially expressed. Functional enrichment analysis of the differentially expressed genes was carried out via KEGG pathway over-representation analysis using the clusterProfiler R package (v4.14.0), with all expressed genes used as the background. Pathways with an adjusted p-value < 0.05 were deemed significantly enriched. Additionally, GSEA was applied to a genome-wide gene list ranked by $\log_2$ fold change, using KEGG gene sets. Enrichment scores were calculated, and their significance was evaluated by permutation testing. Significantly enriched gene sets (FDR < 0.05) were identified to interpret transcriptomic alterations from a functional perspective.

GST pulldown assay

GST-tagged HDAC5 was expressed in E. coli BL21(DE3) bacteria transformed with the corresponding GST-HDAC5 construct. Protein expression was induced with 0.5 mM IPTG at 16°C for 16 hours. Bacterial cells were then lysed, and GST-HDAC5 fusion proteins were purified using glutathione-Sepharose beads. For the pulldown assay, PANC-1 cells were lysed using IP lysis buffer (Beyotime Biotechnology, Cat# P0013) on ice for approximately 0.5 hours, followed by ultrasonication. The purified GST-HDAC5 protein beads were incubated with the cell lysates overnight at 4°C. Beads were washed eight times on ice with ice-cold binding buffer (20 mmol/L Tris, 100 mmol/L NaCl, 1 mmol/L EDTA, 5% Glycerol, 1 mmol/L DTT, 1 mmol/L PMSF, 1 mmol/L Benzamidine), and bound proteins were eluted using ice-cold IP lysis buffer, followed by Western blot analysis.

Mass spectrometry

To investigate the acetylated lysine sites of c-Myc, PANC-1 cells were transfected with Flag-c-Myc for 24 hours under conditions with or without shHDAC5 lentiviral infection. Acetylated peptides were then enriched using anti-acetyl-lysine antibody-conjugated magnetic beads (Cell Signaling Technology, Cat#13416) and subjected to mass spectrometry analysis at Jingjie PTM Biolab Co., Ltd. (Hangzhou, China).

To identify the ubiquitinated lysine sites of c-Myc, PANC-1 cells transfected with Flag-c-Myc for 24 hours were treated with or without shHDAC5 lentiviral infection. The harvested cells were subsequently sent to Bioprofile Biotechnology Co., Ltd. (Shanghai, China) for mass spectrometry analysis.

To elucidate HDAC5-regulated c-Myc-interacting proteins, PANC-1 cells transfected with Flag-c-Myc for 24 hours were processed with or without shHDAC5 lentiviral infection. Cells were lysed using IP buffer (Beyotime, Cat#P0013) and proteins were purified with protein A/G agarose beads (Beyotime, Cat#P2055). The purified proteins were then sent to Jingjie PTM Biolab Co., Ltd. (Hangzhou, China) for mass spectrometry analysis.

Immunohistochemistry (IHC)

IHC staining intensity and the percentage of positive cells were evaluated with the assistance of Image J software. Staining intensity was scored as follows: 0 = negative, 1 = weak staining, 2 = moderate staining, and 3 = strong staining. The staining score was calculated using the formula: H-Score = (percentage of positive cells at each intensity level × staining intensity score) × 100, and then summed. The assessments

were conducted by two independent pathologists who were blinded to the experimental conditions. Detailed antibody information is provided in Supplemental Table 2. The IHC experiments were conducted at BIOSSCI Biotech Co., Ltd. (Hubei, China).

Colony Formation Assay

Pancreatic cancer cells (500 cells per well) were seeded into 6-well plates with 2 mL of DMEM containing 10% FBS. After incubation for two weeks, cells were fixed with 4% paraformaldehyde for 30 minutes, stained with 1% Crystal Violet Staining Solution (Beyotime, #C0121) for 20 minutes, and then washed three times with PBS.

CCK-8 Assay

Pancreatic cancer cells were seeded into 96-well plates at a density of 3000 cells per well in 200 μl of DMEM containing 10% FBS and cultured for 5 days. One hour before the incubation period ended, 20 μl of CCK-8 reagent (Beyotime, #C0037) was added to each well as per the manufacturer's instructions, and the optical absorbance of each well at 450 nm was measured using a microplate reader.

3D-culture

Pancreatic cancer cells were cultured in a three-dimensional (3D) system with Matrigel (Cat#356234, Corning). Briefly, the cells were trypsinized, centrifuged, and re-suspended in DMEM to a final concentration of 3×10^5 cells/mL. The cell suspension was then plated into a 24-well plate that had been pre-coated with Matrigel. The plate was incubated at 37°C for 30 minutes. Subsequently, DMEM mixed with 10% Matrigel was added, and cells were cultured for 7 days, with the Matrigel-containing medium replaced every 2 days. Cell growth was evaluated using the CCK8 assay.

Immunofluorescence Assay

Pancreatic cancer cells were treated with Lyso-Tracker (Thermo Fisher Scientific, USA) for 20 minutes, fixed with paraformaldehyde for 15 minutes, and permeabilized with 0.2% Triton X-100 for 10 minutes. The cells were then incubated with primary antibodies at 4°C overnight, followed by incubation with fluorescent secondary antibodies for 1 hour. Cell nuclei were stained with DAPI for 10 minutes and subsequently analyzed using confocal microscopy (Andor, Dragonfly, 633 objective lens). The antibodies used in the immunofluorescence assay are listed in Supplemental Table 2.

In Vitro Ubiquitination Assay

In vitro ubiquitination assays were performed in ubiquitination reaction buffer (50 mM Tris, pH 7.4, 5 mM $MgCl_2$, 2 mM dithiothreitol). The reaction contained human recombinant E1 (100 ng, Abcam), human recombinant E2 UbcH5c (200 ng, Merck Millipore), and ubiquitin (10 μg, Boston Biochem). NEDD4 protein was immunopurified from the cell lysate of cells overexpressing HA-NEDD4. Additionally, 2 μg Flag-c-Myc (WT/K148R/K148Q) protein was expressed and purified from E. coli Rosetta (DE3). The ubiquitination reaction mixture (30 μl) was incubated at 30°C for 2 hours, followed by boiling for 5 minutes with loading buffer. Finally, ubiquitination levels were analyzed by Western blotting using ubiquitin and Flag antibodies.

Spontaneous Pancreatic Cancer Model

Eight-week-old, sex-matched KPC$^{\textit{Hdac5}\text{-WT}}$ and KPC$^{\textit{Hdac5}\text{-KO}}$ transgenic mice were maintained under SPF conditions. Starting at 10 weeks of age, mice were treated with

MRTX1133 (30 mg/kg, i.p., bid; Selleck Chemicals, Cat#E1051), MYCi975 (50 mg/kg, i.p., bid; Selleck Chemicals, Cat#S8906), HRS-4642 (10 mg/kg, i.v., q.w.; Selleck Chemicals, Cat#E4651) or an equivalent volume of vehicle control (DMSO/PBS). Tumor growth was monitored twice weekly. After 5 weeks, the mice were euthanized, and the pancreas was collected for imaging and subsequent analysis.

Orthotopic Implantation of Pancreatic Cancer Model

Four-week-old C57BL/6 mice were randomly divided into eight groups, with five mice per group. Mice in different experimental groups were orthotopically injected with 5×10^5 KPC-Luc cells infected with different lentiviruses, suspended in 10 μL PBS, and injected into the tail of the pancreas. Tumor growth in vivo was monitored twice per week. Upon tumor formation (day 7), mice treated with MRTX1133 (30mg/kg, i.p., bid) or an equivalent volume of vehicle control (DMSO/PBS). On day 28 of the experiment, mice were subjected to in vivo bioluminescence imaging (Lago X) before euthanasia. The housing conditions were maintained at a temperature of 21°C–27°C, relative humidity of 40%–60%, and a 12-hour light/dark cycle.

Patient-Derived Xenografts (PDX)

The PDAC tissue samples used in this study were obtained from Wuhan Union Hospital, and informed consent was obtained from the patients. Surgically resected PDAC tissues were pathologically confirmed, dissected from primary tumors, and fragmented into small pieces. The tissue was implanted subcutaneously into the left back of NOG mice (F0 generation). When the tumor volume reached approximately 500-800 mm^3, the xenografts were harvested and re-implanted into another group of NOG mice (F1

generation). When the tumor volume in F1 generation mice reached 100 mm^3, mice treated with MRTX1133 (30 mg/kg, i.p., bid), GFH-375 (30 mg/kg, p.o., bid; provided by GenFleet) or an equivalent volume of vehicle control (DMSO/PBS). Concurrently, Lentivirus-sgMYC (5×10^8 TU/mL) was injected subcutaneously at five points around and in the center of the tumor (10 μl per point, weekly) or Lentivirus-sgControl in equivalent volume. After 30 days of treatment, all mice were euthanized, and PDX tissues were collected for IHC analysis. Tumor volume was measured every three days., calculated as follows: tumor volume (mm^3) $=(\text{width})^2\times\text{length}\times1/2$.

Organoids

Organoids were established from surgically resected PDAC tissue of the primary tumor, with prior pathological assessment. The tissue was mechanically minced with a scalpel, followed by enzymatic dissociation in a digestion solution containing culture medium and collagenase XI (Sigma-Aldrich, Cat#C9407). The isolated single cells were then suspended in liquid Matrigel, plated in domes, and overlaid with human complete feeding medium. The cultures were passaged biweekly for cell line expansion or characterization, and the organoid size was assessed weekly.

Xenograft Mouse Model

Four-week-old nude mice were obtained from Vital River (China) and housed under SPF conditions. After random assignment, mice were subcutaneously inoculated with 5×10^6 Mia PaCa-2 cells (shNC or shHDAC5). Beginning on day 6 post-implantation, mice received treatment with either vehicle, Sotorasib (3 mg/kg, p.o., q.d., Selleck Chemicals, Cat# S8830), MYCi975 (50 mg/kg, i.p., bid), or their combination. Tumor

volumes were measured at three-day intervals. On day 30, mice were euthanized, and tumors were harvested, weighed, and imaged.

Supplemental Figures and Supplemental Figure legends

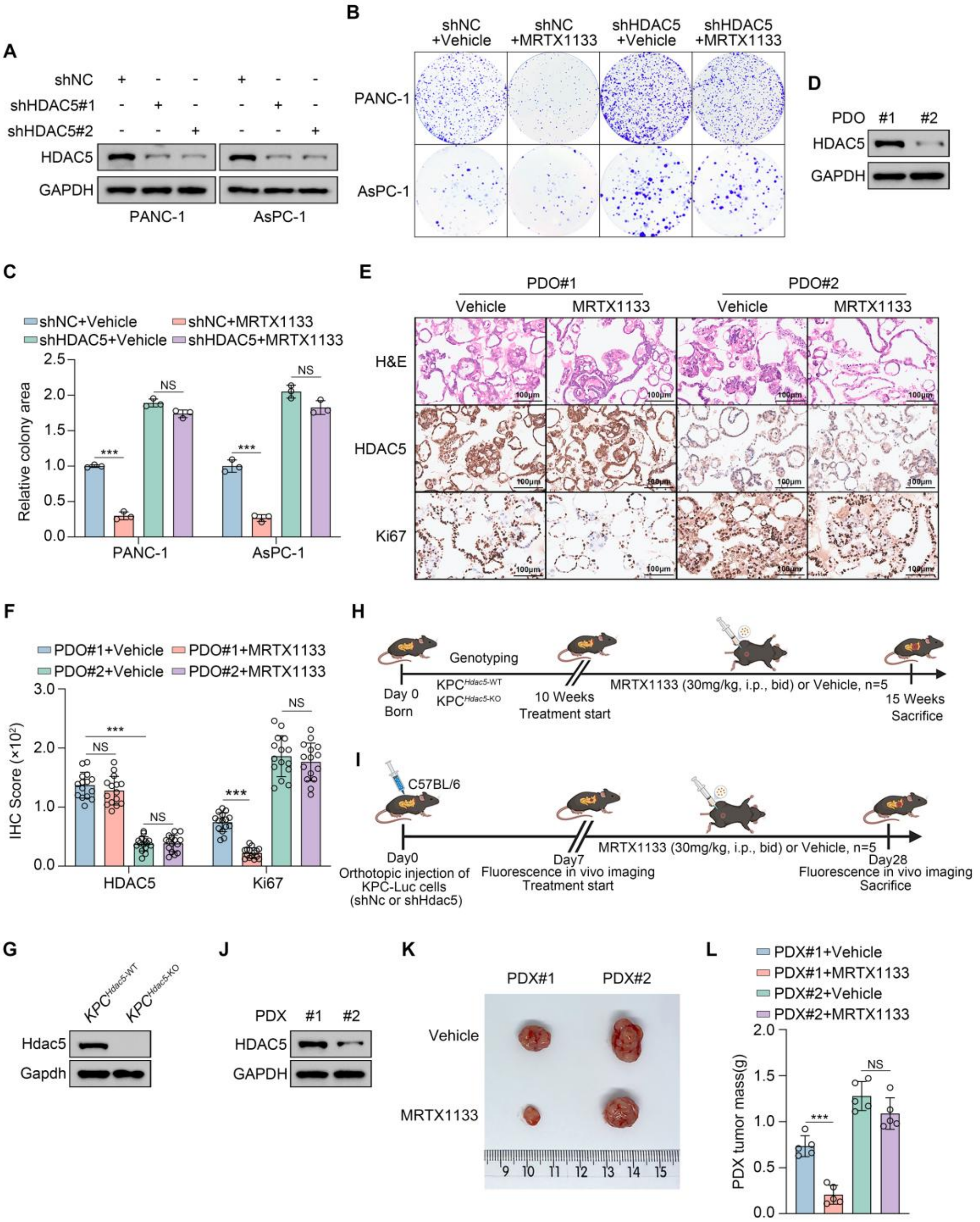

Supplemental Figure 1. Loss of HDAC5 induces intrinsic resistance to MRTX1133. Related to Figure 1 and 2. (A) Western blot analysis shows the HDAC5 knockdown efficiency in PANC-1 and AsPC-1 cells. **(B** and **C)** Colony formation of PANC-1 and AsPC-1 cells expressing indicated shRNAs, treated with DMSO or MRTX1133

(PANC-1: 10 μM; AsPC-1: 5 nM). Colonies were imaged and quantified using ImageJ (n = 3). **(D)** Western blot analysis of HDAC5 protein expression levels in two PDOs. **(E** and **F)** Representative IHC images of PDOs. IHC scores were quantified in **(F)**. Scale bars = 100 μm, n=5 biologically independent repeats and 3 independent quantifications. **(G)** Western blot analysis of Hdac5 protein expression levels in KPC$^{\textit{Hdac5}\text{-WT}}$ and KPC$^{\textit{Hdac5}\text{-KO}}$ mice. **(H)** Schematic representation of in vivo MRTX1133 therapeutic experiments in KPC$^{\textit{Hdac5}\text{-WT}}$ and KPC$^{\textit{Hdac5}\text{-KO}}$ mice models. **(I)** Schematic illustration of in vivo MRTX1133 therapeutic experiments using orthotopic mice models. **(J)** Western blot analysis of HDAC5 protein expression levels in two PDXs. **(K)** Representative images of PDX tumors were collected and photographed following euthanasia. **(L)** PDXs tumor weights (n = 5). All data are presented as the mean ± SD. Statistical significance was determined by two-way ANOVA followed by Tukey multiple comparisons test (**C**, **F** and **L**). NS, not significant; $^{*}P < 0.05$, $^{**}P < 0.01$, $^{***}P < 0.001$.

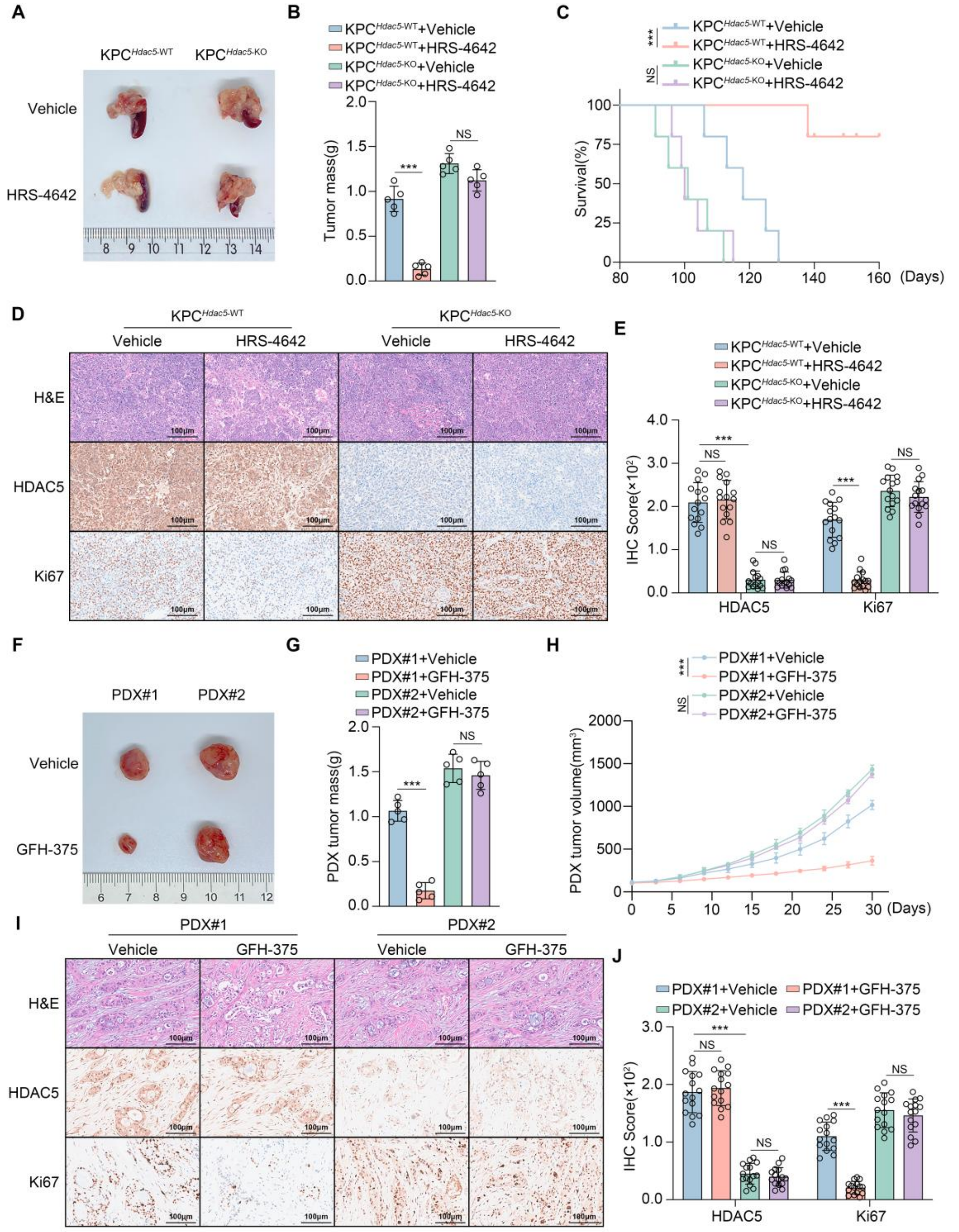

Supplemental Figure 2. Loss of HDAC5 induces intrinsic resistance to HRS4642 and GFH-375 in vivo. Related to Figure 1 and 2. (A) Representative macroscopic images of tumors from KPC$^{Hdac5\text{-WT}}$ and KPC$^{Hdac5\text{-KO}}$ mice treated with vehicle or HRS-4642 (10mg/kg, i.v., q.w.). **(B)** Statistical analysis of tumor weights in KPC$^{Hdac5\text{-WT}}$ and KPC$^{Hdac5\text{-KO}}$ mice (n = 5). **(C)** Kaplan-Meier survival curves with log-rank test (n = 5). **(D** and **E)** Representative IHC images of tumors. IHC scores were quantified in **(E)**.

Scale bars = 100 μm, n = 5 biologically independent repeats and 3 independent quantifications. **(F)** Representative images of PDX tumors treated with vehicle or GFH-375 (30 mg/kg, p.o., bid) were collected and photographed following euthanasia. **(G)** PDX tumor weights (n = 5). **(H)** Tumor volumes over time in PDX models (n = 5). **(I and J)** Representative IHC images of PDXs. IHC scores were quantified in **(J)**. Scale bars = 100 μm, n = 5 biologically independent repeats and 3 independent quantifications. All data are presented as the mean ± SD. Statistical significance was determined by two-way ANOVA followed by Tukey multiple comparisons test (**B**, **E**, **G**, **H** and **J**). NS, not significant; *$P < 0.05$, **$P < 0.01$, ***$P < 0.001$.

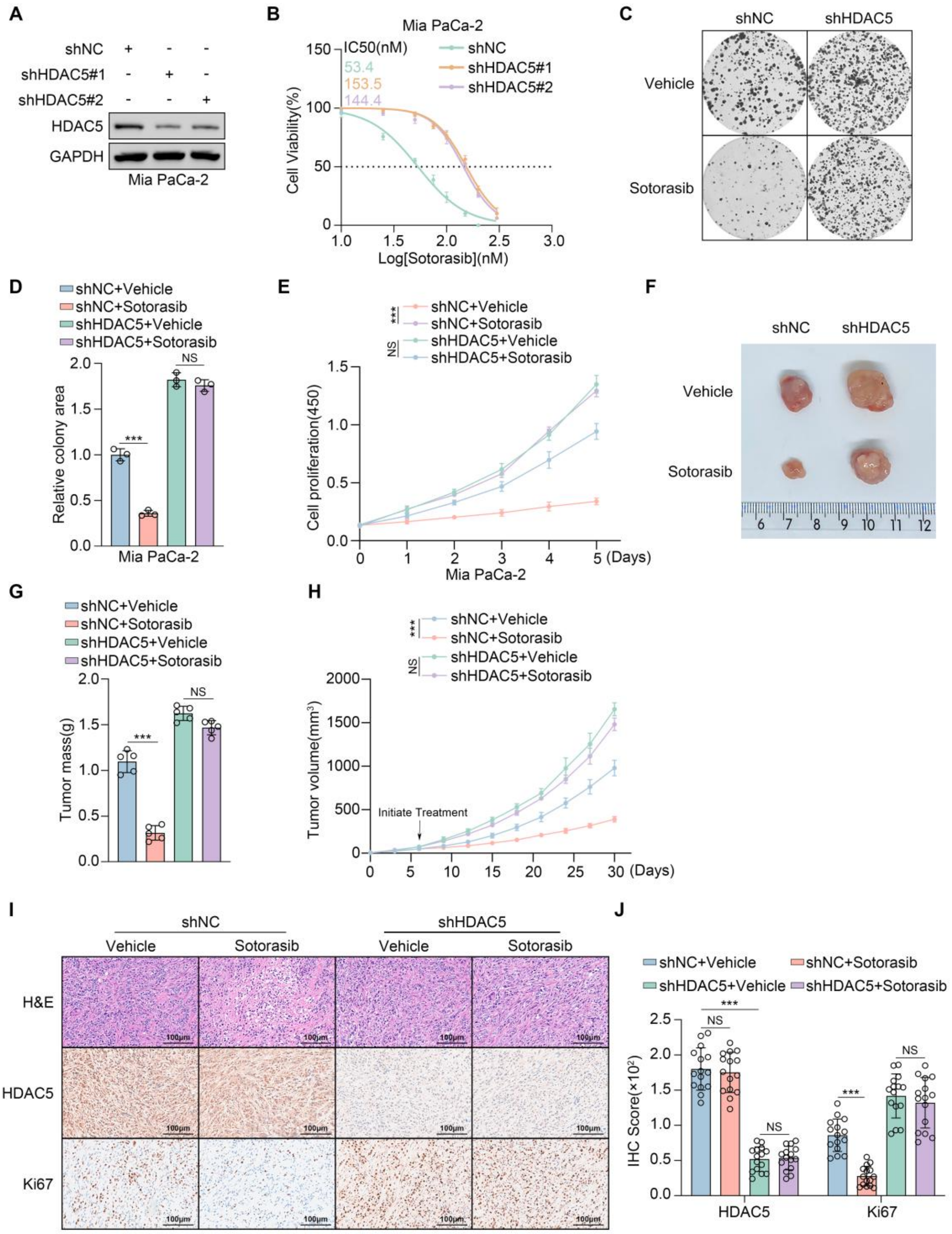

Supplemental Figure 3. Loss of HDAC5 induces intrinsic resistance to KRAS^{G12C} inhibitor. Related to Figure 1 and 2. (A) Western blot analysis shows the HDAC5 knockdown efficiency in Mia PaCa-2 cells. **(B)** IC_{50} values of Sotorasib in Mia PaCa-2 cells with HDAC5 knockdown, measured by CCK-8 assay. (**C** and **D**) Colony formation of Mia PaCa-2 cells expressing indicated shRNAs, treated with Vehicle or Sotorasib (20 nM). Colonies were quantified using ImageJ (n = 3). **(E)** Cell viability of

Mia PaCa-2 cells expressing indicated shRNAs, treated with DMSO or Sotorasib (20 nM) (n=3). **(F)** Mia PaCa-2 cells, with or without HDAC5 knockdown, were subcutaneously implanted into BALB/c-nu mice. Treatments with vehicle or Sotorasib (3 mg/kg, p.o., q.d.) were initiated on day 6 post-implantation. Tumor volumes were measured every 3 days. Mice were euthanized on day 30, and tumors were excised, photographed, and weighed. **(G)** Tumors weight from subcutaneous models (n = 5). **(H)** Tumor volumes over time in subcutaneous models (n = 5). (**I** and **J**) Representative IHC images of subcutaneous models. IHC scores were quantified in **(J)**. Scale bars = 100 µm, n = 5 biologically independent repeats and 3 independent IHC quantifications. All data are presented as the mean ± SD. Statistical significance was determined by two-way ANOVA followed by Tukey multiple comparisons test **(D, E, G, H** and **J)**. NS, not significant; *$P < 0.05$, **$P < 0.01$, ***$P < 0.001$.

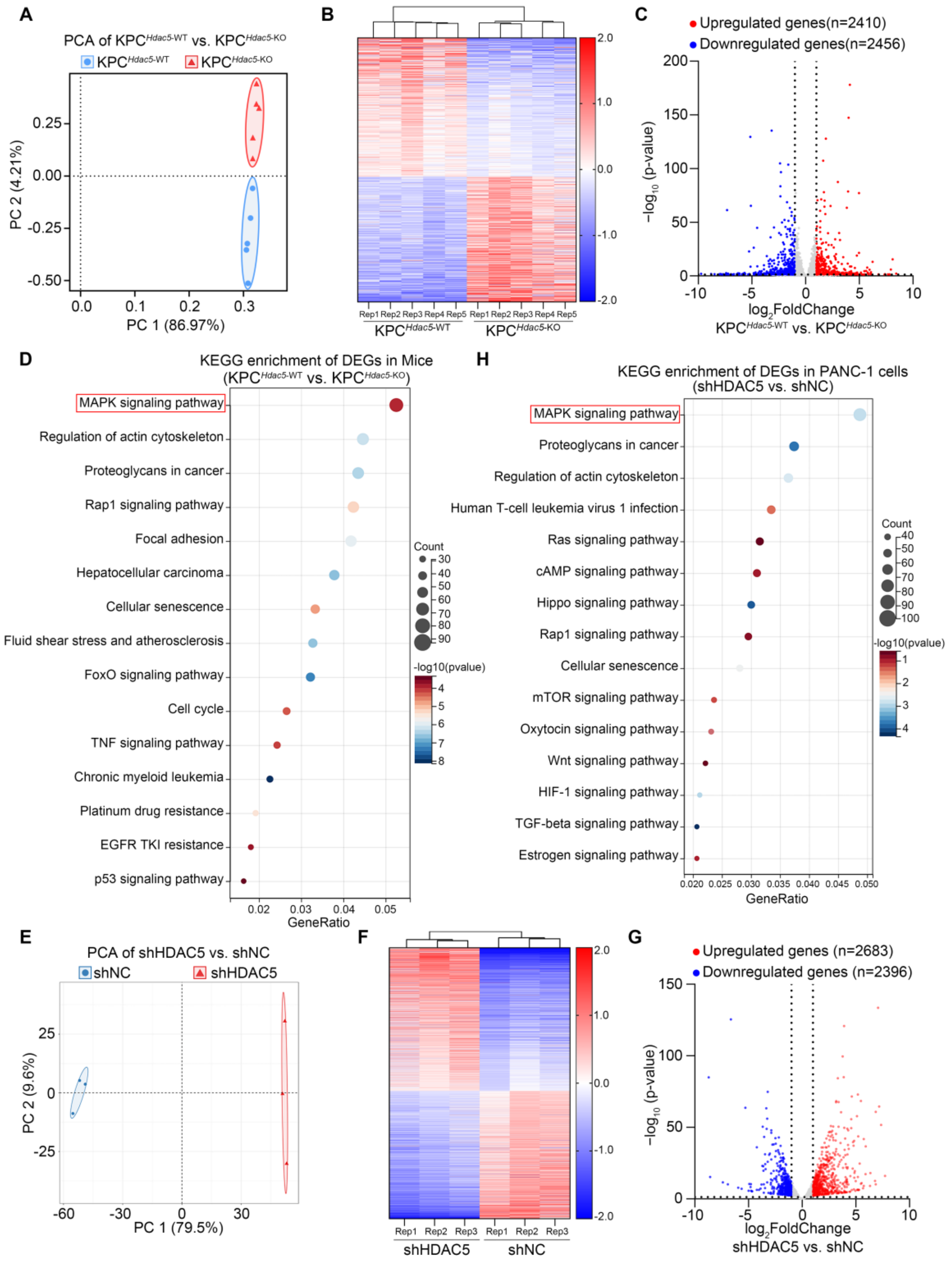

Supplemental Figure 4. Loss of HDAC5 drives MAPK signaling enrichment. Related to Figure 3. (A) Principal component analysis (PCA) of RNA-seq data comparing KPC$^{Hdac5\text{-KO}}$ and KPC$^{Hdac5\text{-WT}}$ (n = 5). (**B** and **C**) Heatmap and volcano plot showing DEGs (| log2 (fold change) | > 1, P < 0.05) in KPC$^{Hdac5\text{-KO}}$ and KPC$^{Hdac5\text{-WT}}$ tumor tissues. Red dots represent upregulated genes, while blue dots represent downregulated genes. **(D)** KEGG pathway enrichment analysis was performed on the

4,866 DEGs that were identified in the volcano plot **(C)**. **(E)** PCA of RNA-seq data comparing shNC and shHDAC5 PANC-1 cells (n = 3). (**F** and **G**) Heatmap and volcano plot showing DEGs (| log2 (fold change) | > 1, $P < 0.05$) in shNC and shHDAC5 PANC-1 cells. Red dots represent upregulated genes, while blue dots represent downregulated genes. **(E)** KEGG pathway enrichment analysis was performed on the 5,079 differentially expressed genes that were identified in the volcano plot **(G)**. **(H)** KEGG pathway enrichment analysis was performed on the 5,079 DEGs that were identified in the volcano plot **(G)**.

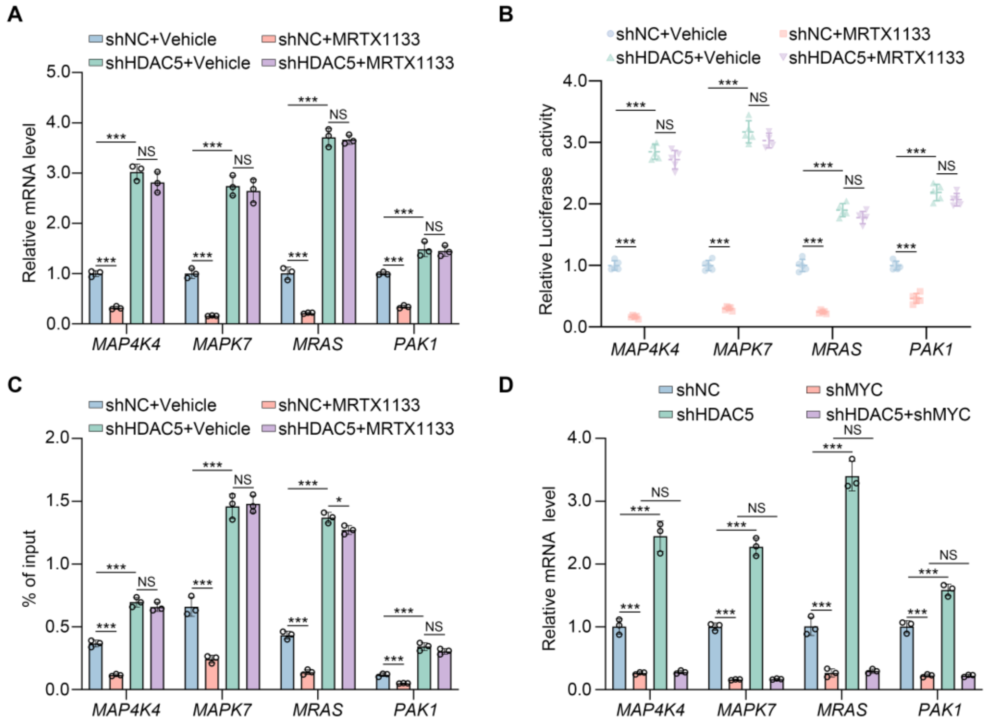

Supplemental Figure 5. Loss of HDAC5 upregulates MAPK signaling via c-Myc. Related to Figure 3. (A) RT-qPCR analysis of MAPK pathway gene mRNA in PANC-1 cells transfected with indicated plasmids and treated with DMSO or MRTX1133 (10μM, 48 h) (n = 3). **(B)** Dual-luciferase reporter assays were performed to assess the transcriptional activity of canonical MAPK pathway genes in PANC-1 cells under the indicated conditions (n = 5). **(C)** ChIP-qPCR analysis of MYC enrichment at the promoter regions of MAPK pathway genes in PANC-1 cells (n = 3). **(D)** RT-qPCR analysis of MAPK pathway mRNA expression under indicated conditions (n = 3). All data are presented as the mean ± SD. Statistical significance was determined by two-way ANOVA followed by Tukey multiple comparisons test **(A, B, C** and **D)**. NS, not significant; *$P < 0.05$, **$P < 0.01$, ***$P < 0.001$.

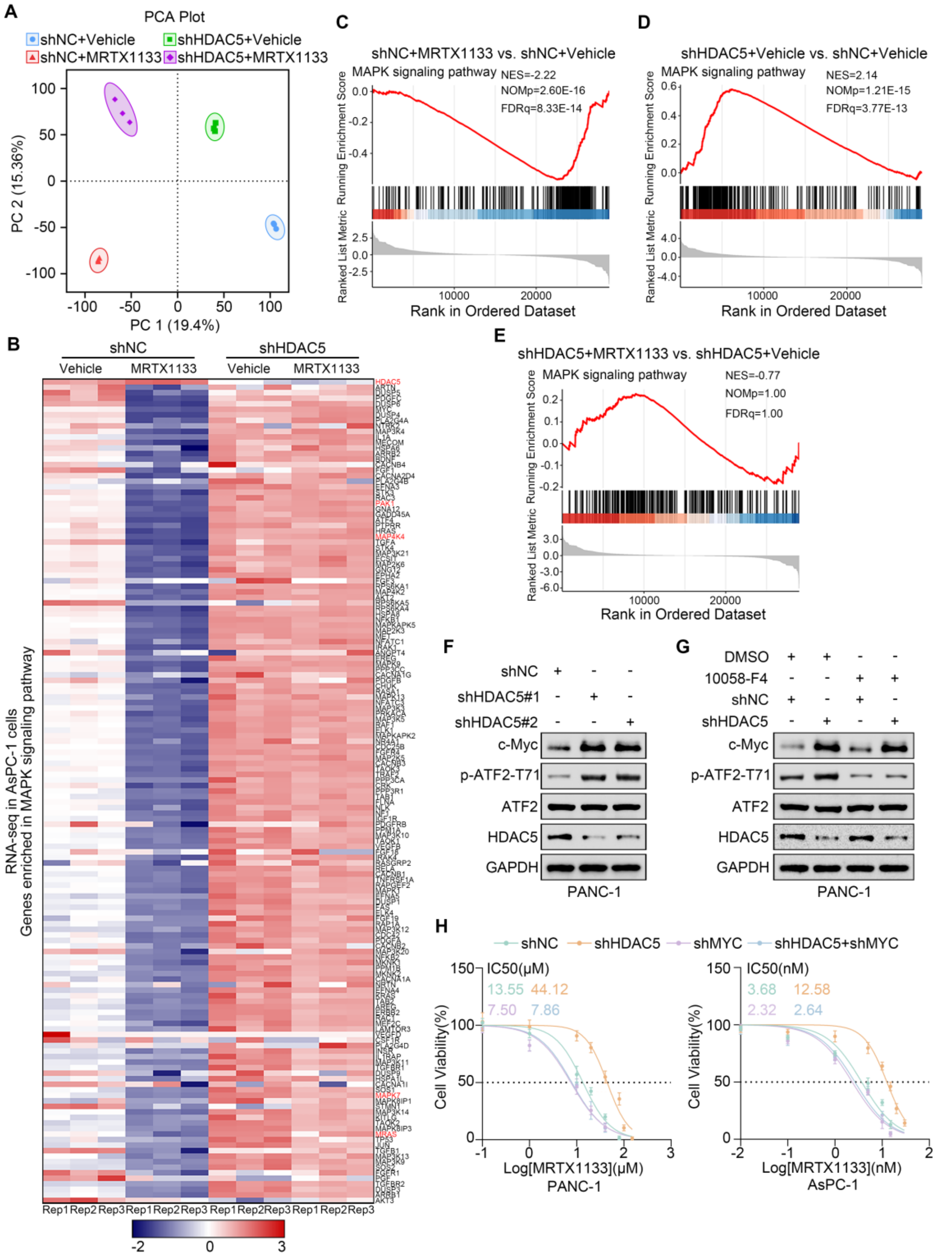

Supplemental Figure 6. Loss of HDAC5 upregulates MAPK signaling via c-Myc. Related to Figure 3. (A) Principal component analysis (PCA) of RNA-seq data from AsPC-1 cells transduced with shNC or shHDAC5 and subsequently treated with DMSO or MRTX1133 (5 nM, 48 h), with three biological replicates per treatment group. **(B)** Heatmap showing the expression of HDAC5 and a subset of genes involved in the MAPK signaling pathway. **(C-E)** Gene set enrichment analysis (GSEA) depicting the

enrichment of MAPK signaling pathway genes between the indicated groups. **(F and G)** PANC-1 cells were transfected with the indicated shRNAs for 48 h and analyzed by Western blot **(F)**. Post-transfection, cells were treated with DMSO or 10058-F4 (50 μM, 24 h) and analyzed by Western blot **(G)**. **(H)** The IC_{50} values of MRTX1133 were determined in PANC-1 and AsPC-1 cells under the indicated conditions using the CCK8 assay.

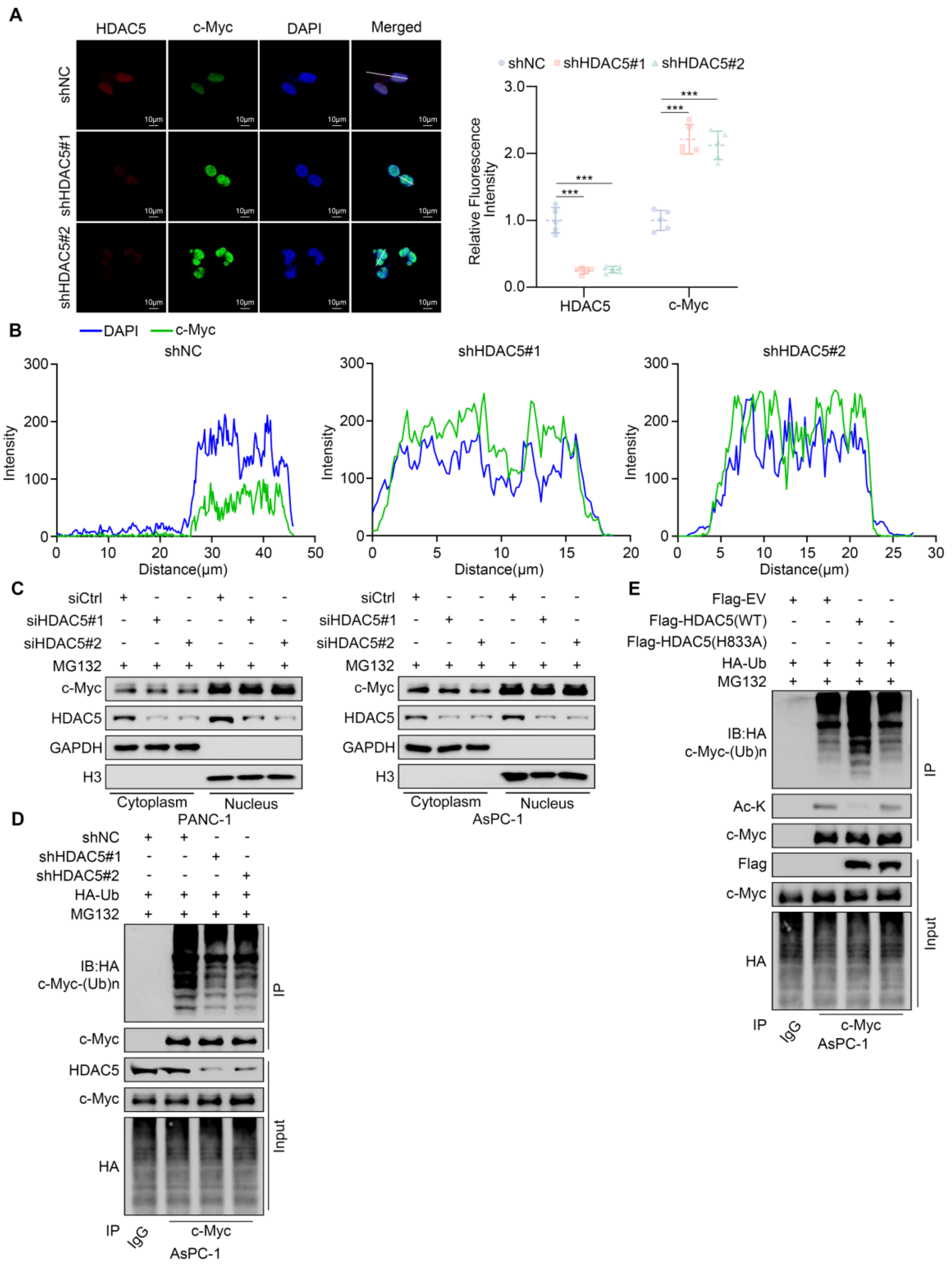

Supplemental Figure 7. HDAC5 promotes c-Myc ubiquitination and degradation through its deacetylation. Related to Figure 4 and 5. (A) PANC-1 cells were transfected with the indicated shRNA for 48 hours and then transferred to culture slides for immunofluorescence. Representative images and fluorescence intensity quantification are shown. Data are shown as mean ± SD (n = 5). Statistical significance was determined by one-way ANOVA followed by Dunnett's multiple comparisons test.

***$P < 0.001$. **(B)** Colocalization analysis of the merged images in **(A)**, showing pixel intensity profiles along the white line from left to right in each panel. Colors correspond to the merged images: green for c-Myc and blue for DAPI. **(C)** Western blot analysis of c-Myc expression in nuclear and cytoplasmic fractions of PANC-1 and AsPC-1 cells transfected with the indicated shRNAs for 48 h, followed by treatment with MG132 (10 μM, 8 h). **(D)** Co-IP assay to detect ubiquitination of c-Myc in HDAC5-deficient AsPC-1 cells. **(E)** Co-IP assay to detect ubiquitination of c-Myc in AsPC-1 cells transfected with the indicated plasmids.

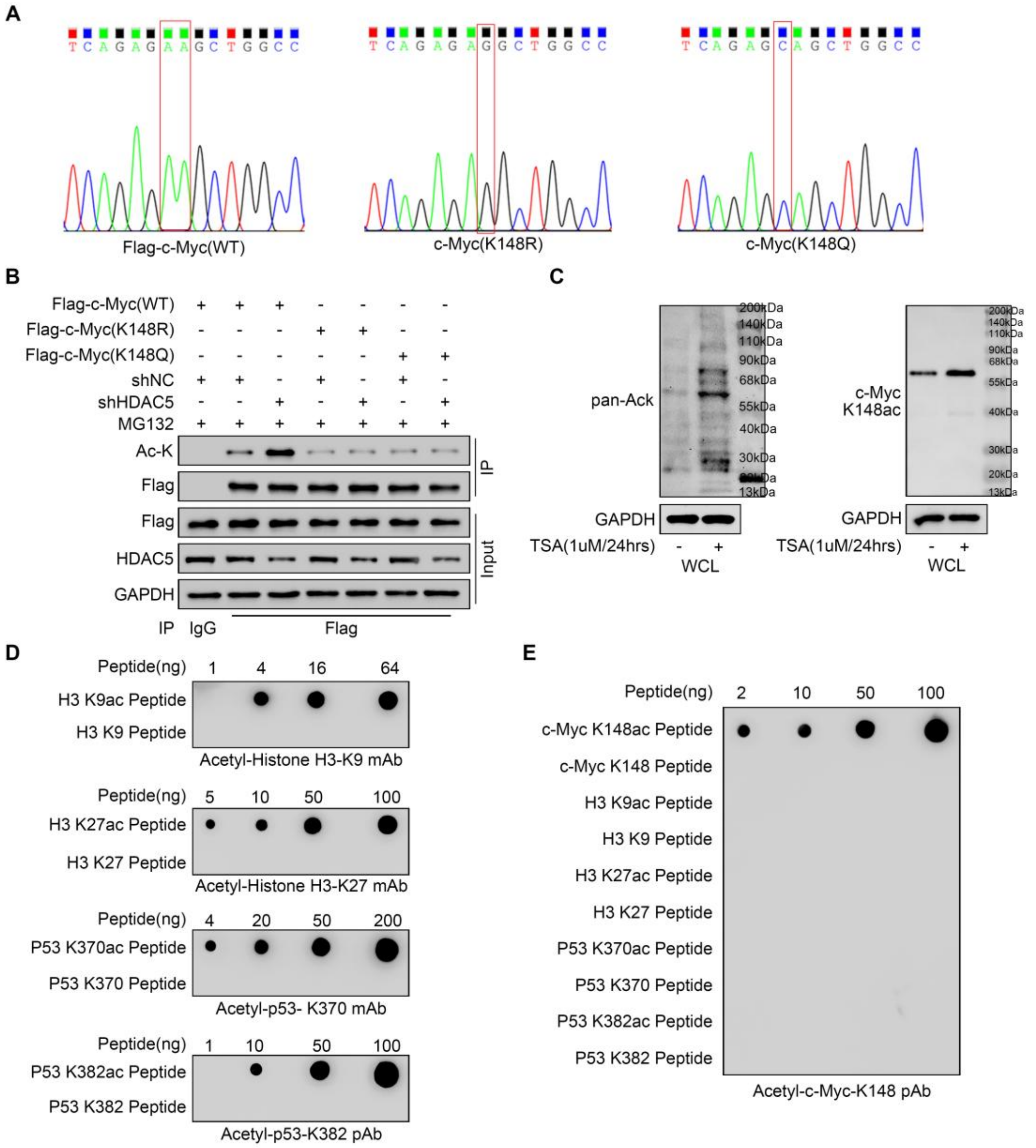

Supplemental Figure 8. HDAC5 loss disrupts the acetylation-ubiquitination homeostasis at lysine 148 of c-Myc. Related to Figure 6. (A) Sanger DNA sequencing traces of c-Myc (WT/ K148R/ K148Q) plasmid. **(B)** PANC-1 cells were transfected with equal amounts of c-Myc (WT/K148R/K148Q) plasmids, followed by knockdown with shNC or shHDAC5, and c-Myc acetylation was assessed. **(C)** PANC-1 cells were treated with DMSO or TSA (1 µM, 18 h). Whole-cell lysates were analyzed by Western blot using a pan–acetyl-lysine antibody (left) and a c-Myc K148ac–specific antibody (right). **(D and E)** Synthetic peptides dissolved in ddH_2O were spotted onto nitrocellulose membranes for dot blot analysis. Peptide integrity was verified by

immunoblotting using the corresponding antibodies **(D)**. The same peptides were probed with the anti–c-Myc-K148ac antibody to assess its specificity **(E)**.

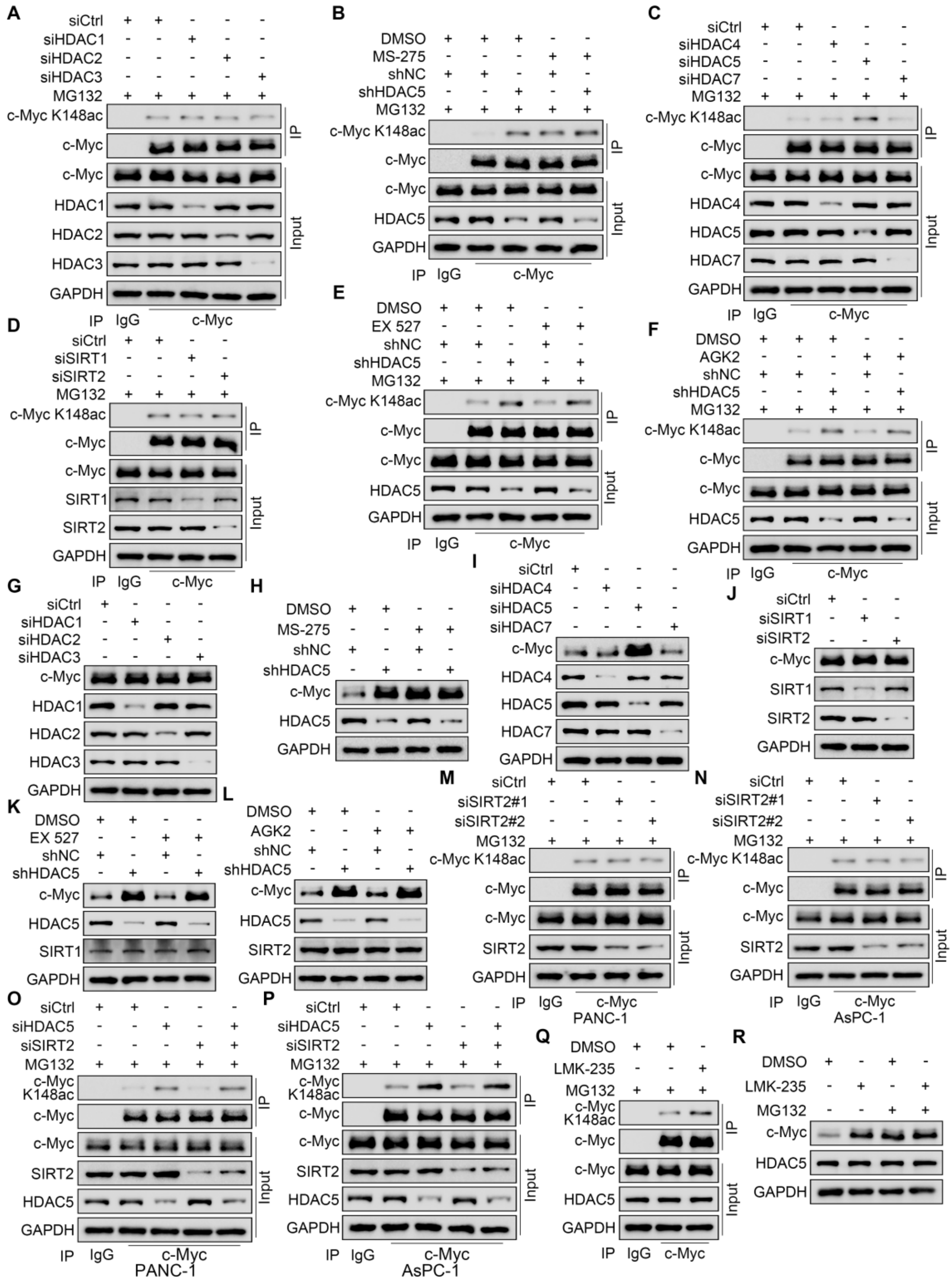

Supplemental Figure 9. HDAC5-specific deacylation of c-Myc K148 regulates protein stability. Related to Figure 6. (A-F) PANC-1 cells were treated under the indicated conditions (MS-275, 5 μM, 24 h; EX527, 10 μM, 8 h; AGK2, 10 μM, 24 h; MG132, 10 μM, 8 h), followed by western blot analysis of c-Myc K148 acetylation. **(G-L)** PANC-1 cells were treated under the indicated conditions, followed by western blot analysis of c-Myc protein level. **(M-P)** PANC-1 and AsPC-1 cells were transfected

with the indicated siRNAs, treated with MG132 (10 μM, 8 h), and analyzed for c-Myc K148 acetylation by western blotting. **(Q** and **R)** PANC-1 cells were treated with LMK-235 (1 μM, 24 h) and MG132 (10 μM, 8 h), followed by western blot analysis of c-Myc K148 acetylation **(Q)** and total protein levels **(R)**.

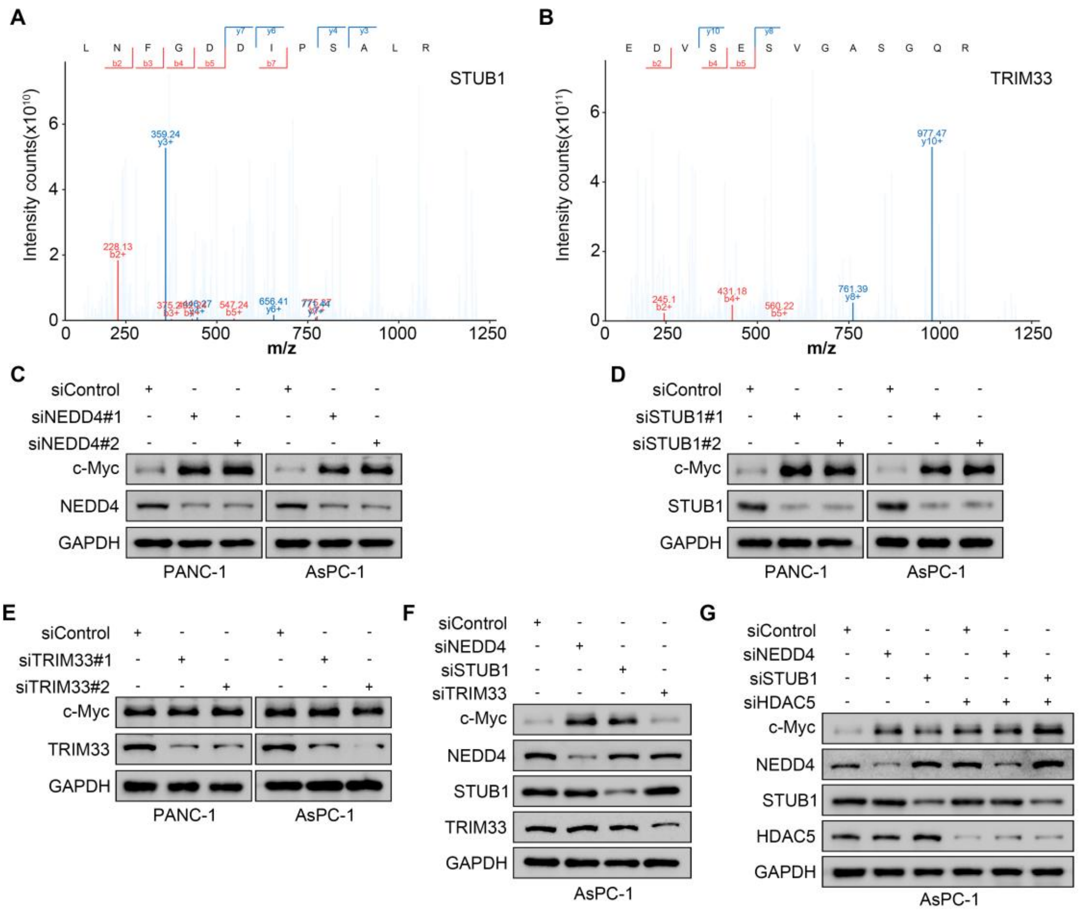

Supplemental Figure 10. HDAC5 and NEDD4 cooperatively regulate c-Myc protein stability. Related to Figure 7. (A and B) STUB1**(A)** and TRIM33**(B)** peptide fragment was precipitated with c-Myc antibody by MS, showing significantly reduced signals in the shHDAC5-treated group compared to the control group. **(C-E)** PANC-1 and AsPC-1 cells were infected with indicated siRNAs for 48 hours, cells were harvested for Western blot analysis. **(F and G)** AsPC-1 cells were infected with indicated siRNAs for 48 hours, cells were harvested for Western blot analysis.

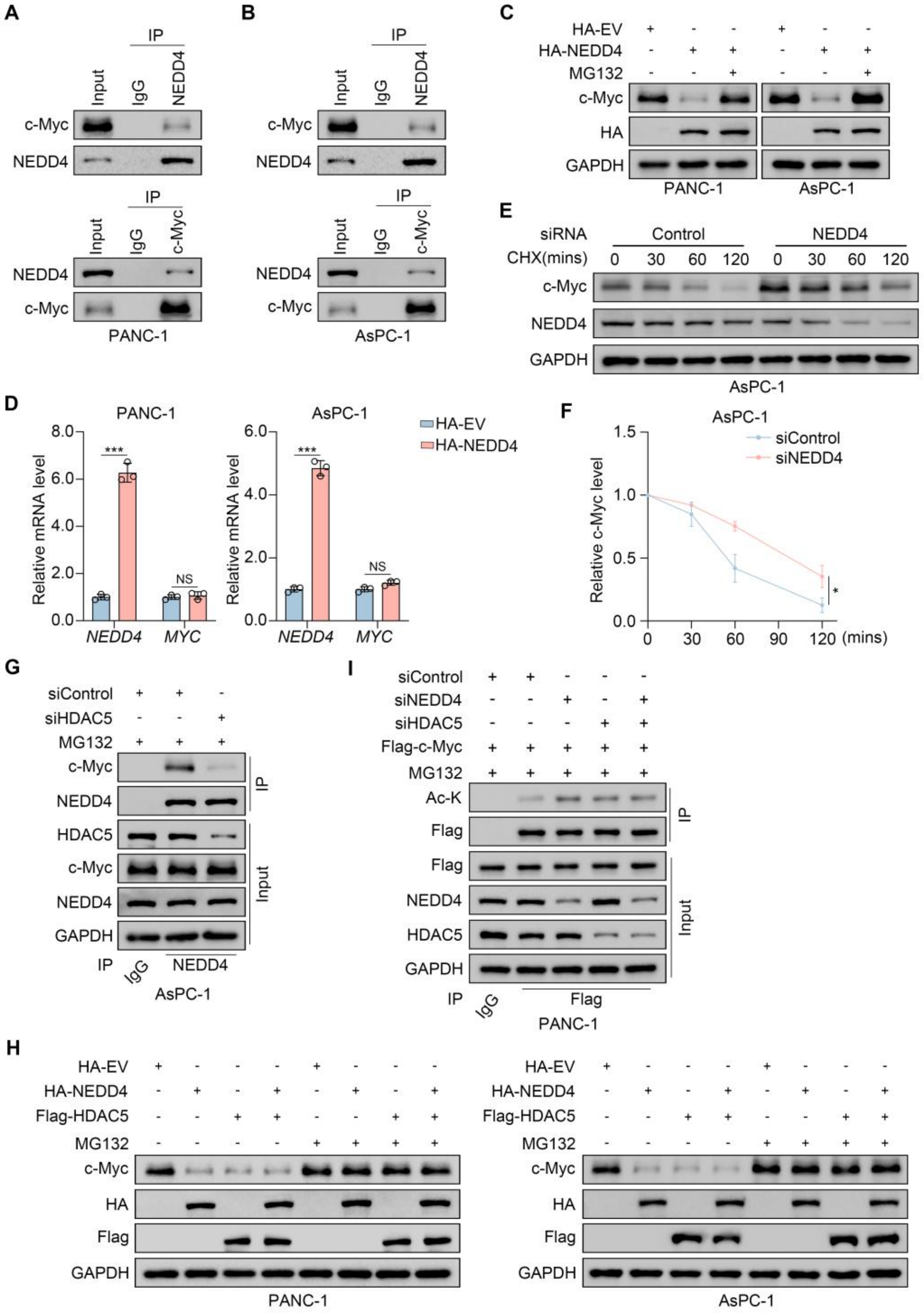

Supplemental Figure 11. HDAC5-mediated c-Myc deacetylation facilitates NEDD4-mediated ubiquitination at K148 of c-Myc. Related to Figure 7. (A and **B)** Co-IP assay indicating the interacting relationship of c-Myc and NEDD4 in PANC-1 cells **(A)** and AsPC-1 cells **(B)**. **(C)** Western blot analysis of c-Myc protein levels in PANC-1 and AsPC-1 cells transfected with indicated plasmids and treated with DMSO or MG132 (10μM, 8 h). **(D)** RT-qPCR analysis of PANC-1 and AsPC-1 cells infected

with indicated plasmids for 48 h. Data are mean ± SD (n = 3). Statistical significance was determined by unpaired two-tailed t-test. NS, not significant, ***$P < 0.001$. **(E and F)** Western blot analysis of c-Myc protein stability in control and NEDD4-knockdown AsPC-1 cells treated with 50 μg/mL CHX for the indicated times. Data are shown as mean ± SD (n=3). Statistical significance was determined by two-way ANOVA followed by Tukey multiple comparisons test. * $P < 0.05$. **(G)** AsPC-1 cells were transfected with the indicated siRNAs and then harvested for endogenous Co-IP analysis to evaluate the interaction between NEDD4 and c-Myc. **(H)** Western blot analysis of c-Myc protein levels in PANC-1 and AsPC-1 cells transfected with indicated plasmids and treated with DMSO or MG132 (10μM, 8 h). **(I)** PANC-1 cells were transfected with equal amounts of c-Myc (WT) plasmids, followed by knockdown with siHDAC5, siNEDD4, or both, and c-Myc acetylation was assessed.

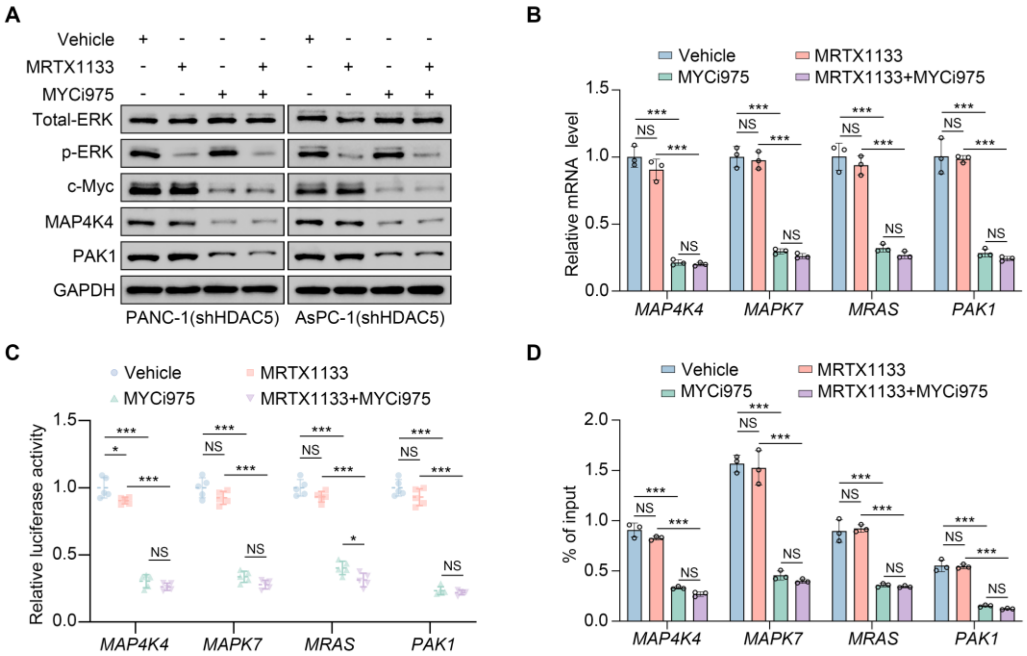

Supplemental Figure 12. MYCi975 suppresses MAPK pathway reactivation driven by HDAC5 loss. Related to Figure 8 and 9. (A) Western blot analysis of canonical MAPK pathway proteins in HDAC5-deficient PANC-1 and AsPC-1 cells following treatment under the indicated conditions (MRTX1133: PANC-1, 10 μM, 48 h; AsPC-1, 5 nM, 48 h; MYCi975: 10 μM, 48 h). **(B)** RT-qPCR analysis of mRNA levels of canonical MAPK pathway genes in HDAC5-deficient PANC-1 cells under the indicated conditions (MRTX1133: PANC-1, 10 μM, 48 h; AsPC-1, 5 nM, 48 h; MYCi975: 10 μM, 48 h) (n=3). **(C)** Dual-luciferase reporter assays were performed to assess the transcriptional activity of genes in the canonical MAPK pathway in HDAC5-deficient PANC-1 cells under the indicated conditions (MRTX1133: PANC-1, 10 μM, 48 h; AsPC-1, 5 nM, 48 h; MYCi975: 10 μM, 48 h) (n=5). **(D)** ChIP-qPCR analysis of MYC enrichment at the promoters of genes in the canonical MAPK pathway in HDAC5-deficient PANC-1 cells under the indicated conditions (MRTX1133: PANC-1, 10 μM, 48 h; AsPC-1, 5 nM, 48 h; MYCi975: 10 μM, 48 h) (n=3). All data are presented as the mean ± SD. Statistical significance was determined by two-way ANOVA followed by Tukey multiple comparisons test (**B, C** and **D**). NS, not significant; *$P < 0.05$, **$P < 0.01$, ***$P < 0.001$.

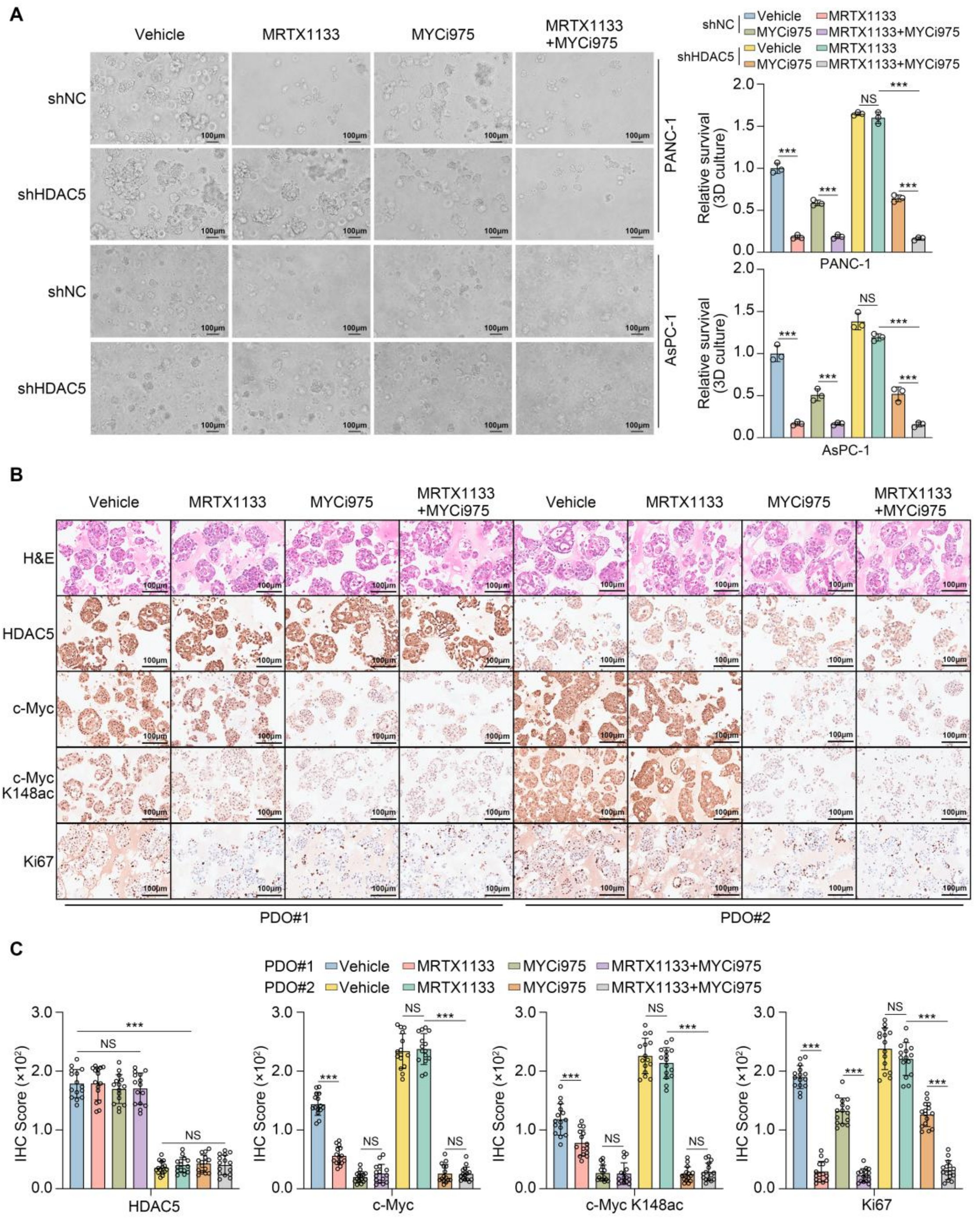

Supplemental Figure 13. Pharmacological inhibition of c-Myc overcomes the resistance to MRTX113 induced by HDAC5 loss in vitro. Related to Figure 8. (A) Representative images of 3D cultures of PANC-1 and AsPC-1 cells infected with indicated shRNAs. Cells were treated with vehicle (DMSO), MRTX1133 (10 μM for PANC-1, 5 nM for AsPC-1), MYCi975 (10 μM) or their combination for 48 hours post-infection (n=3). **(B)** Representative IHC images of PDOs treated with vehicle (DMSO),

MRTX1133 (1 μM, 5 days), MYCi975 (4 μM, 5 days) or their combination. **(C)** IHC scores were quantified in **(B)**. Scale bars = 100 μm. n = 5 biologically independent repeats and 3 independent IHC quantifications. All data are presented as the mean ± SD. Statistical significance was determined by two-way ANOVA followed by Tukey multiple comparisons test (**A** and **C**). NS, not significant; **P* < 0.05, ***P* < 0.01, ****P* < 0.001.

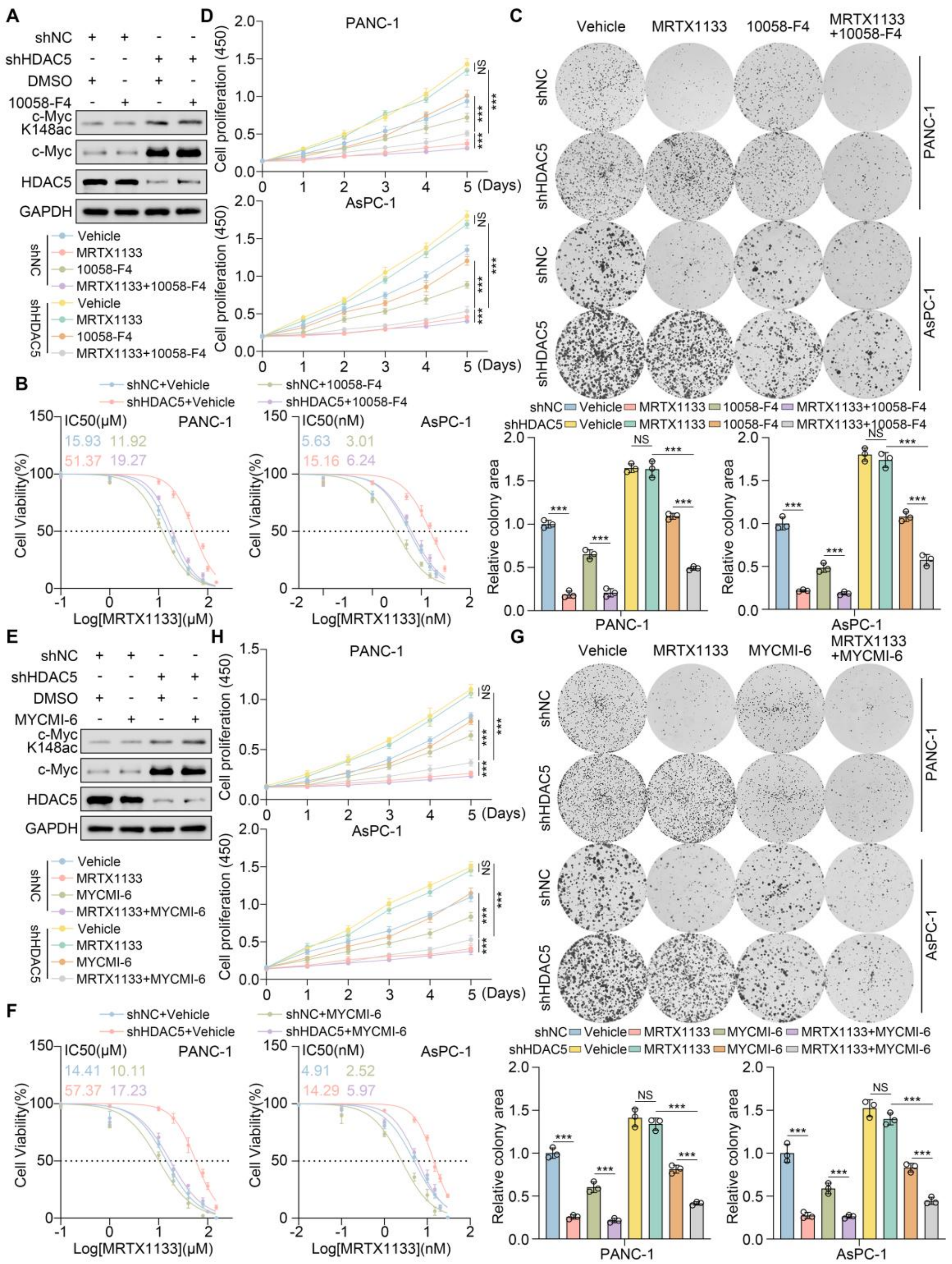

Supplemental Figure 14. Pharmacological targeting of c-Myc reverses HDAC5 loss–mediated resistance to MRTX1133. Related to Figure 8. (A) Western blot analysis of c-Myc K148 acetylation and total c-Myc protein levels in PANC-1 cells transfected with the indicated shRNAs for 48 h, followed by treatment with vehicle (DMSO) or 10058-F4 (50 μM, 24 h). **(B)** PANC-1 and AsPC-1 cells were infected with the indicated shRNAs for 48 h, and the IC_{50} of MRTX1133 was assessed by CCK-8

assay after treatment with vehicle (DMSO) or 10058-F4 (50 μM). **(C)** PANC-1 and AsPC-1 cells were infected with the indicated shRNAs for 48 h, then treated with vehicle (DMSO), MRTX1133 (10 μM for PANC-1, 5 nM for AsPC-1), 10058-F4 (50 μM) or their combination. Colony formation was assessed, and colonies were imaged and quantified using ImageJ (n = 3). **(D)** PANC-1 and AsPC-1 cells were infected with the indicated shRNAs for 48 h, followed by treatment with vehicle (DMSO), MRTX1133 (10 μM for PANC-1, 5 nM for AsPC-1), 10058-F4 (50 μM) or their combination, and subjected to the CCK-8 assay (n = 3). **(E-H)** The same experiments were performed using MYCMI-6 (0.5 μM) instead of 10058-F4. All data are presented as the mean ± SD. Statistical significance was determined by two-way ANOVA followed by Tukey multiple comparisons test (**C, D, G** and **H**). NS, not significant; *$P < 0.05$, **$P < 0.01$, ***$P < 0.001$.

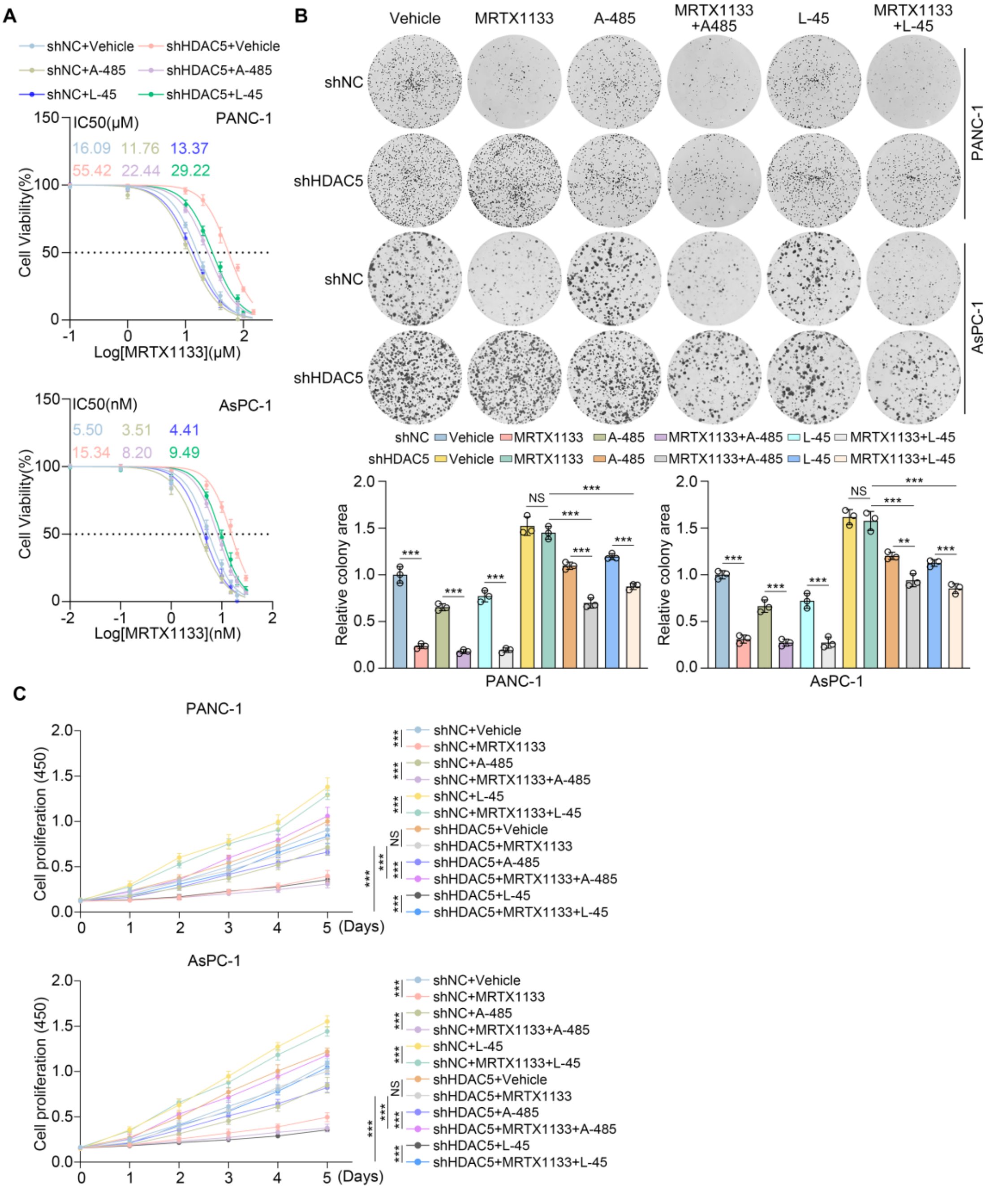

Supplemental Figure 15. p300/CBP and PCAF inhibitors partially reverse HDAC5 loss–induced MRTX1133 resistance. Related to Figure 8. (A) PANC-1 and AsPC-1 cells were infected with the indicated shRNAs for 48 h, and the IC_{50} of MRTX1133 was determined by CCK-8 assay after treatment with vehicle (DMSO), A-485 (0.5 μM, 48 h), or L-45 (0.1 μM, 48 h). **(B)** PANC-1 and AsPC-1 cells were infected with the indicated shRNAs for 48 h, followed by treatment under the indicated conditions (A-

485: 0.5 μM; L-45: 0.1 μM; MRTX1133: 10 μM for PANC-1, 5 nM for AsPC-1). Colony formation was assessed and quantified using ImageJ (n = 3). **(C)** PANC-1 and AsPC-1 cells were infected with the indicated shRNAs for 48 h, followed by treatment under the indicated conditions (A-485: 0.5 μM; L-45: 0.1 μM; MRTX1133: 10 μM for PANC-1, 5 nM for AsPC-1), and then subjected to the CCK-8 assay (n = 3). All data are presented as the mean ± SD. Statistical significance was determined by two-way ANOVA followed by Tukey multiple comparisons test **(B** and **C)**. NS, not significant; $*P < 0.05$, $**P < 0.01$, $***P < 0.001$.

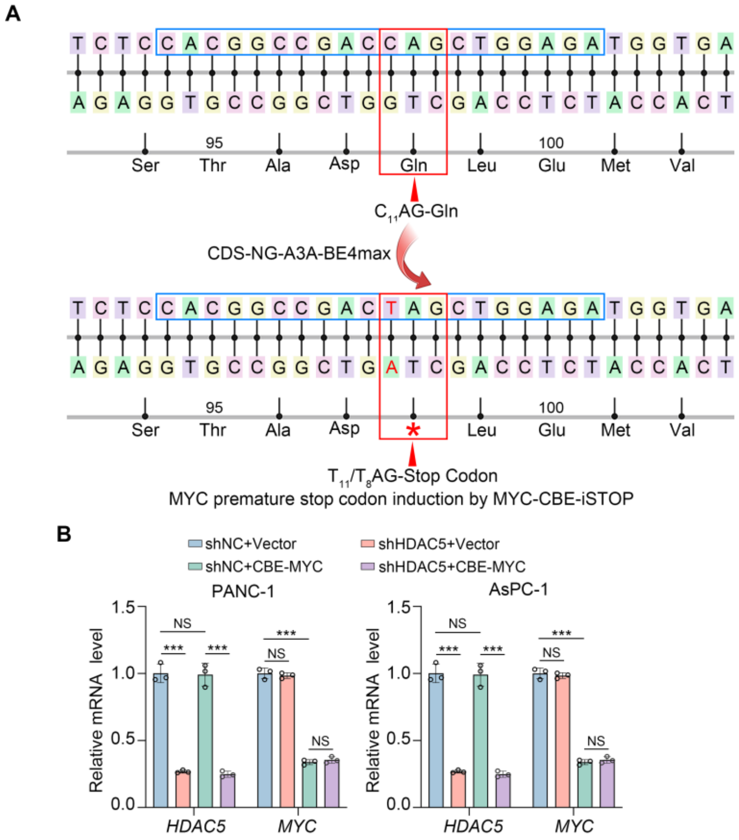

Supplemental Figure 16. Efficient MYC gene Editing by CBE. Related to Figure 8. (A) Schematic illustration of introducing a stop codon into the *MYC* gene using CBE. **(B)** The editing efficiency of CBE targeting the *MYC* gene in PANC-1 and AsPC-1 cells was validated at the mRNA level by RT-qPCR analysis. Data are presented as the mean ± SD. Statistical significance was determined by two-way ANOVA followed by Tukey multiple comparisons test. NS, not significant, $***P < 0.001$.

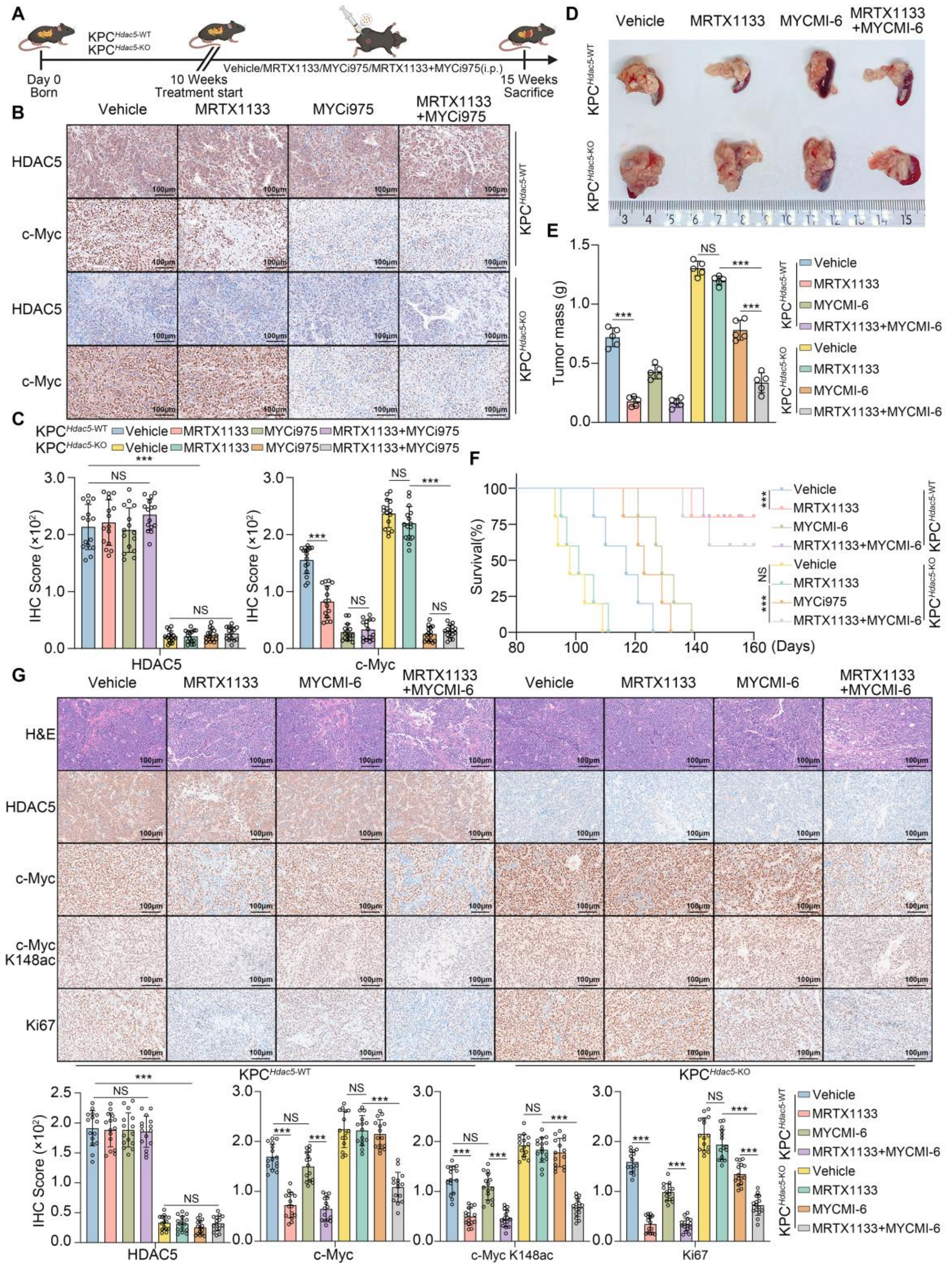

Supplemental Figure 17. Pharmacological inhibition of c-Myc overcomes the resistance to MRTX113 induced by HDAC5 loss in vivo. Related to Figure 9. (A) Schematic representation of in vivo therapeutic experiments using vehicle (DMSO), MRTX1133 (30 mg/kg, i.p. bid), MYCi975 (50mg/kg, i.p. bid) or their combination in KPC$^{Hdac5\text{-}WT}$ and KPC$^{Hdac5\text{-}KO}$ mice models. **(B and C)** Representative IHC images of tumors from KPC$^{Hdac5\text{-}WT}$ and KPC$^{Hdac5\text{-}KO}$ mice treated with vehicle (DMSO),

MRTX1133 (30 mg/kg, i.p. bid), MYCi975 (50mg/kg, i.p. bid) or their combination. IHC scores were quantified in **(C)**. Scale bars=100μm. n=5 biologically independent repeats. **(D)** Representative macroscopic images of pancreatic tumors from KPC$^{Hdac5\text{-WT}}$ and KPC$^{Hdac5\text{-KO}}$ mice treated with vehicle, MRTX1133 (30 mg/kg, i.p., bid), MYCMI-6 (20 mg/kg, i.p., q.d.) or their combination. **(E)** Statistical analysis of tumor weights (n = 5). **(F)** Kaplan-Meier survival curves with log-rank test (n = 5). NS, not significant; ***P < 0.001. **(G)** Representative IHC images of KPC$^{Hdac5\text{-WT}}$ and KPC$^{Hdac5\text{-KO}}$ tumors. IHC scores were quantified. Scale bars = 100 μm, n = 5 biologically independent repeats and 3 independent IHC quantifications. Data are presented as the mean ± SD. Statistical significance was determined by two-way ANOVA followed by Tukey multiple comparisons test **(C, E** and **G)**. NS, not significant; *P < 0.05, **P < 0.01, ***P < 0.001.

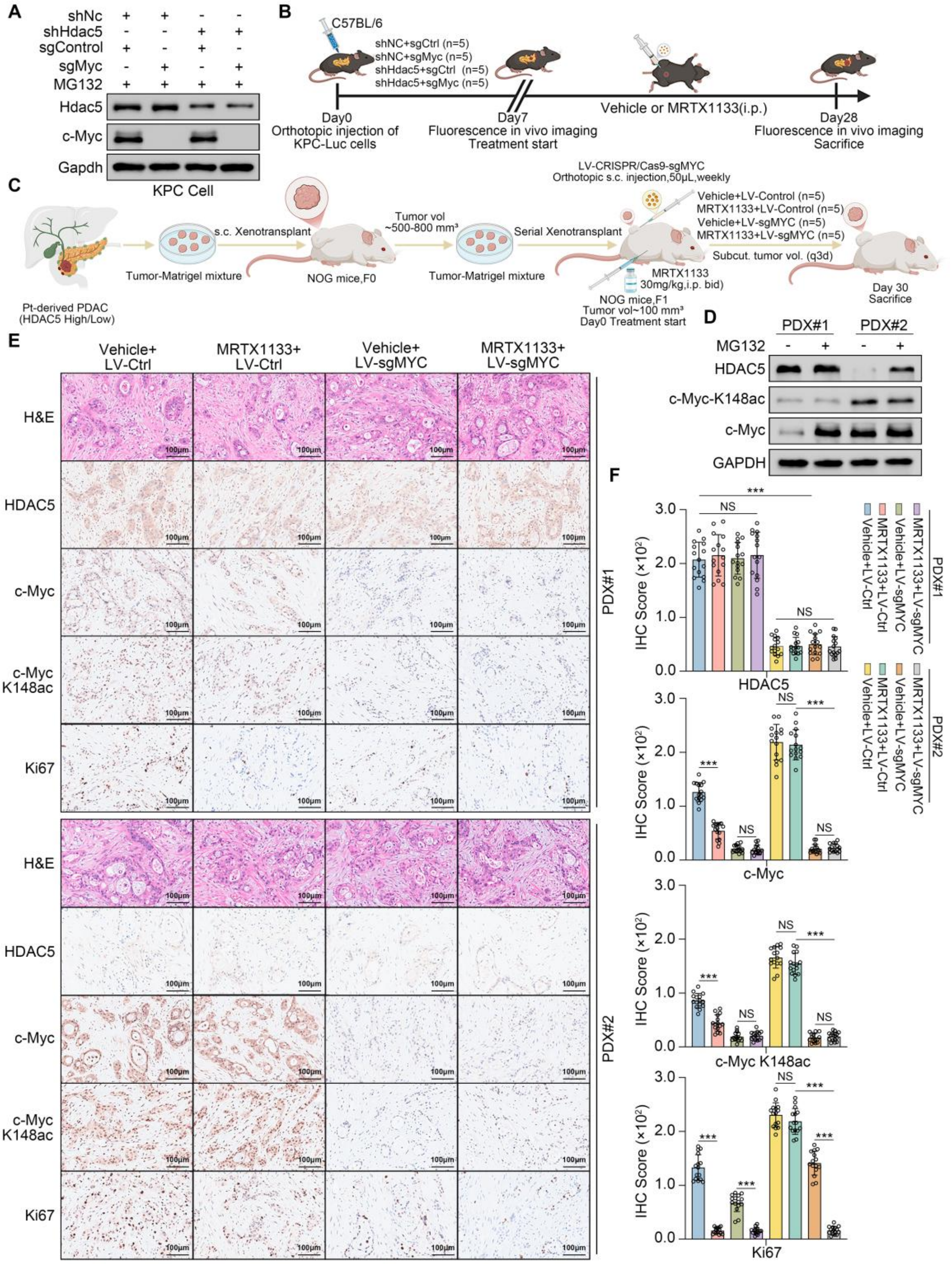

Supplemental Figure 18. Genetic inhibition of MYC overcomes the resistance to MRTX113 induced by HDAC5 loss in vivo. Related to Figure 9. (A) Western blot analysis of Hdac5 and c-Myc knockout efficiency in KPC cells. **(B)** Schematic representation of in vivo therapeutic experiments using vehicle, MRTX1133 (30 mg/kg, i.p., bid), MYCi975 (50 mg/kg, i.p., bid) or their combination, in orthotopic mouse models established with KPC-Luc cells stably expressing shHDAC5 and sgMYC after

infection with indicated lentiviral plasmids. **(C)** The schematic illustrates the construction of the PDX model and the subsequent treatment regimen. **(D)** Western blot analysis of c-Myc-K148 acetylation and indicated protein expression in PDXs. **(E and F)** Representative IHC images of tumors from PDXs. IHC scores were quantified. Scale bars=100μm. Data are shown as mean ± SD, n=5 biologically independent repeats. Statistical significance was determined by two-way ANOVA followed by Tukey multiple comparisons test. NS, not significant; *$P < 0.05$, **$P < 0.01$, ***$P < 0.001$.

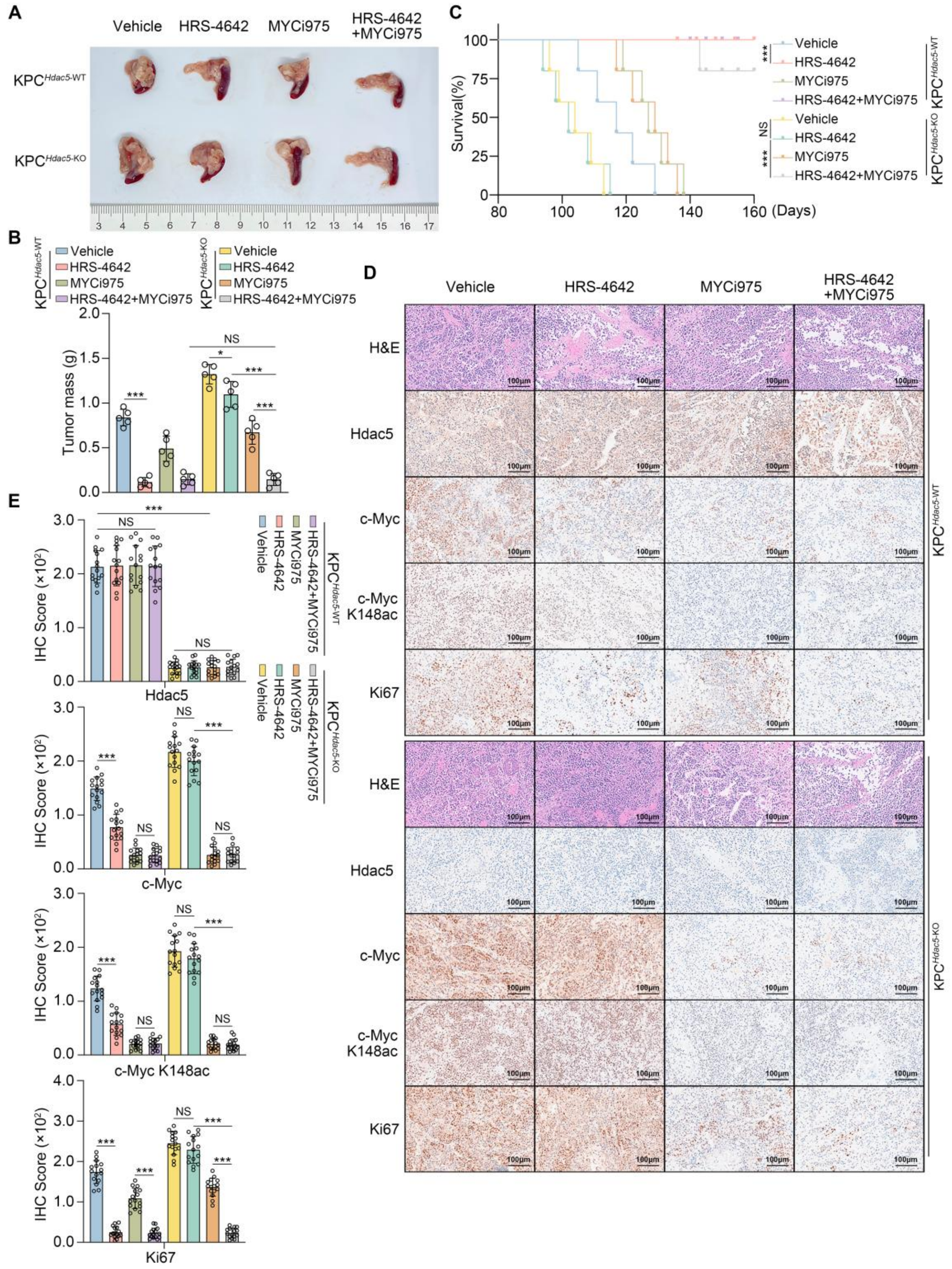

Supplemental Figure 19. Pharmacological inhibition of c-Myc overcomes the resistance to HRS-4642 induced by HDAC5 loss in vivo. Related to Figure 9. (A) Representative macroscopic images of pancreatic tumors from KPC$^{Hdac5\text{-WT}}$ and KPC$^{Hdac5\text{-KO}}$ mice treated with vehicle, HRS-4642 (10 mg/kg, i.v., q.w.), MYCi975 (50 mg/kg, i.p., bid) or their combination. **(B)** Statistical analysis of tumor weights in KPC$^{Hdac5\text{-WT}}$ and KPC$^{Hdac5\text{-KO}}$ mice treated with vehicle, HRS-4642, MYCi975 or their

combination (n = 5). **(C)** Kaplan–Meier survival curves analyzed by log-rank test (n = 5). NS, not significant; ***$P < 0.001$. **(D** and **E)** Representative IHC images of tumors treated with vehicle, HRS-4642, MYCi975 or their combination. IHC scores were quantified **(E)**. Scale bars = 100 μm. n = 5 biologically independent repeats and 3 independent IHC quantifications. All data are presented as the mean ± SD. Statistical significance was determined by two-way ANOVA followed by Tukey multiple comparisons test. NS, not significant; *$P < 0.05$, **$P < 0.01$, ***$P < 0.001$.

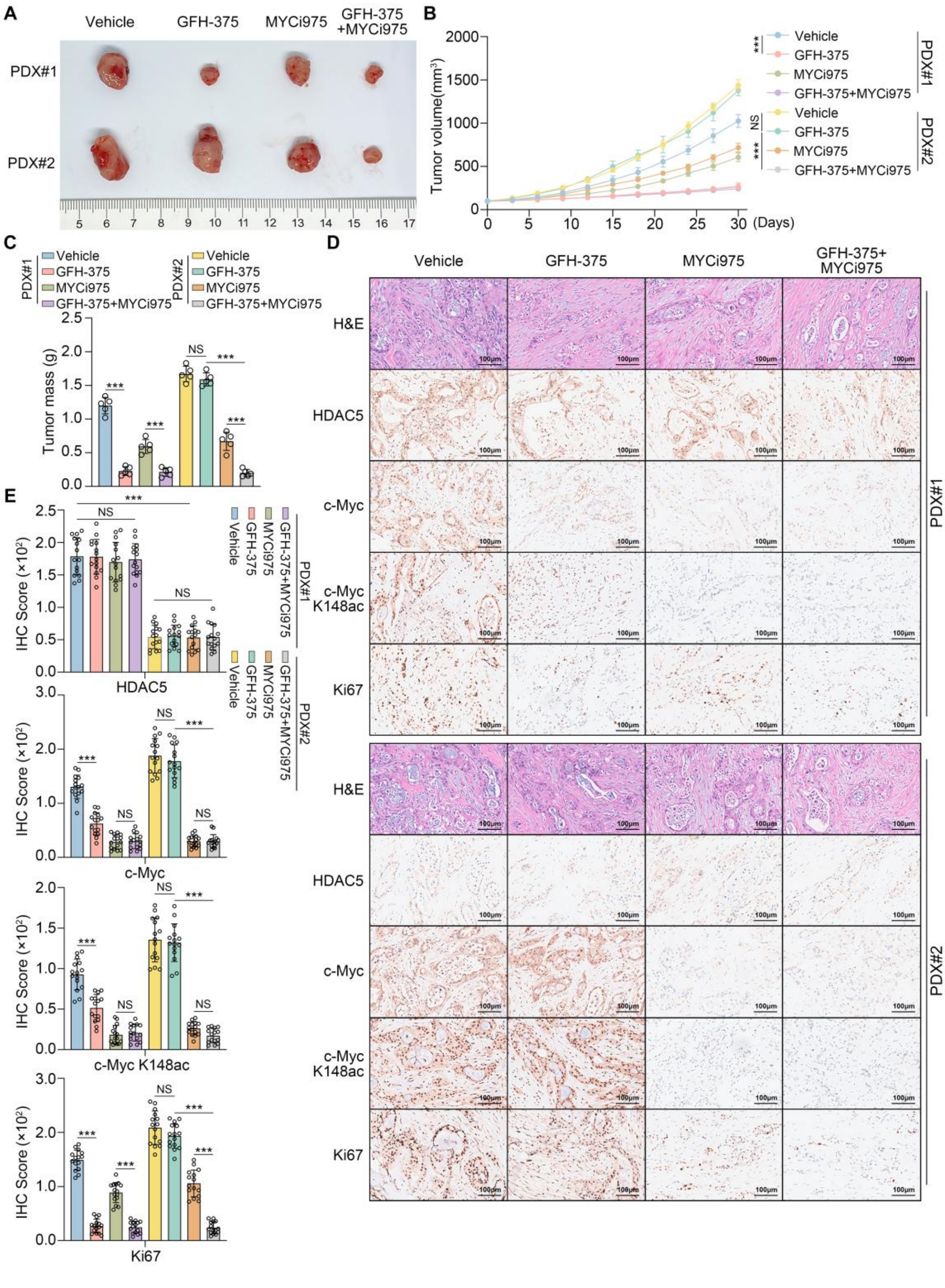

Supplemental Figure 20. Pharmacological inhibition of c-Myc overcomes the resistance to GFH-375 induced by HDAC5 loss. Related to Figure 9. (A) Representative images of PDX tumors treated with vehicle, GFH-375 (30 mg/kg, p.o., bid), MYCi975 (50 mg/kg, i.p., bid), or their combination, collected and photographed at day 30 post-euthanasia. **(B)** Tumor volumes over time in PDX#1 and PDX#2 models treated with vehicle, GFH-375, MYCi975 or their combination (n = 5). **(C)** Weight of

tumors harvested from PDX models treated with vehicle, GFH-375, MYCi975 or their combination (n = 5). **(D** and **E)** Representative IHC images of PDXs. IHC scores were quantified **(E)**. Scale bars = 100 μm. n = 5 biologically independent repeats and 3 independent IHC quantifications. All data are presented as the mean ± SD. Statistical significance was determined by two-way ANOVA followed by Tukey multiple comparisons test **(B, C** and **E)**. NS, not significant; $^{*}P < 0.05$, $^{**}P < 0.01$, $^{***}P < 0.001$.

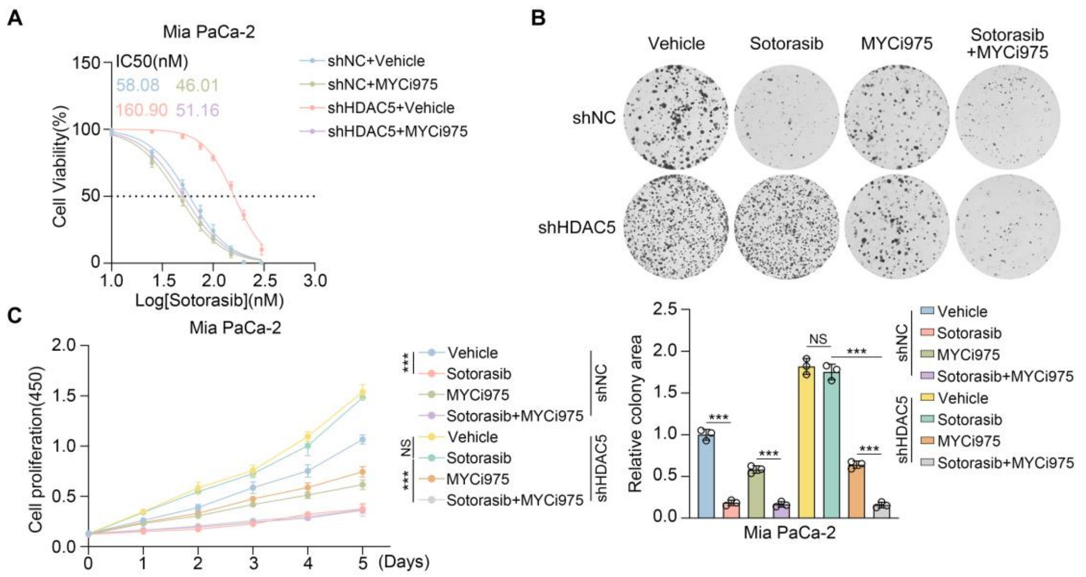

Supplemental Figure 21. Pharmacological inhibition of c-Myc overcomes resistance to $KRAS^{G12C}$ inhibitors induced by HDAC5 loss in vitro. Related to Figure 9. (A) Mia PaCa-2 cells were infected with the indicated shRNAs for 48 h, and the IC_{50} of Sotorasib was assessed by CCK-8 assay following treatment with Vehicle (DMSO) or MYCi975 (10μM, 48 h). **(B)** Mia PaCa-2 cells were infected with the indicated shRNAs for 48 h, then treated with vehicle, Sotorasib (20 nM), MYCi975(10 μM), or their combination. Colony formation was assessed, and colonies were imaged and quantified using ImageJ (n = 3). **(C)** Mia PaCa-2 cells were infected with the indicated shRNAs for 48 h, followed by treatment with vehicle, Sotorasib (20 nM), MYCi975(10 μM), or their combination, and subsequently subjected to the CCK-8 assay (n = 3). All data are presented as the mean ± SD. Statistical significance was determined by two-way ANOVA followed by Tukey multiple comparisons test **(B** and **C)**. NS, not significant; *$P < 0.05$, **$P < 0.01$, ***$P < 0.001$.

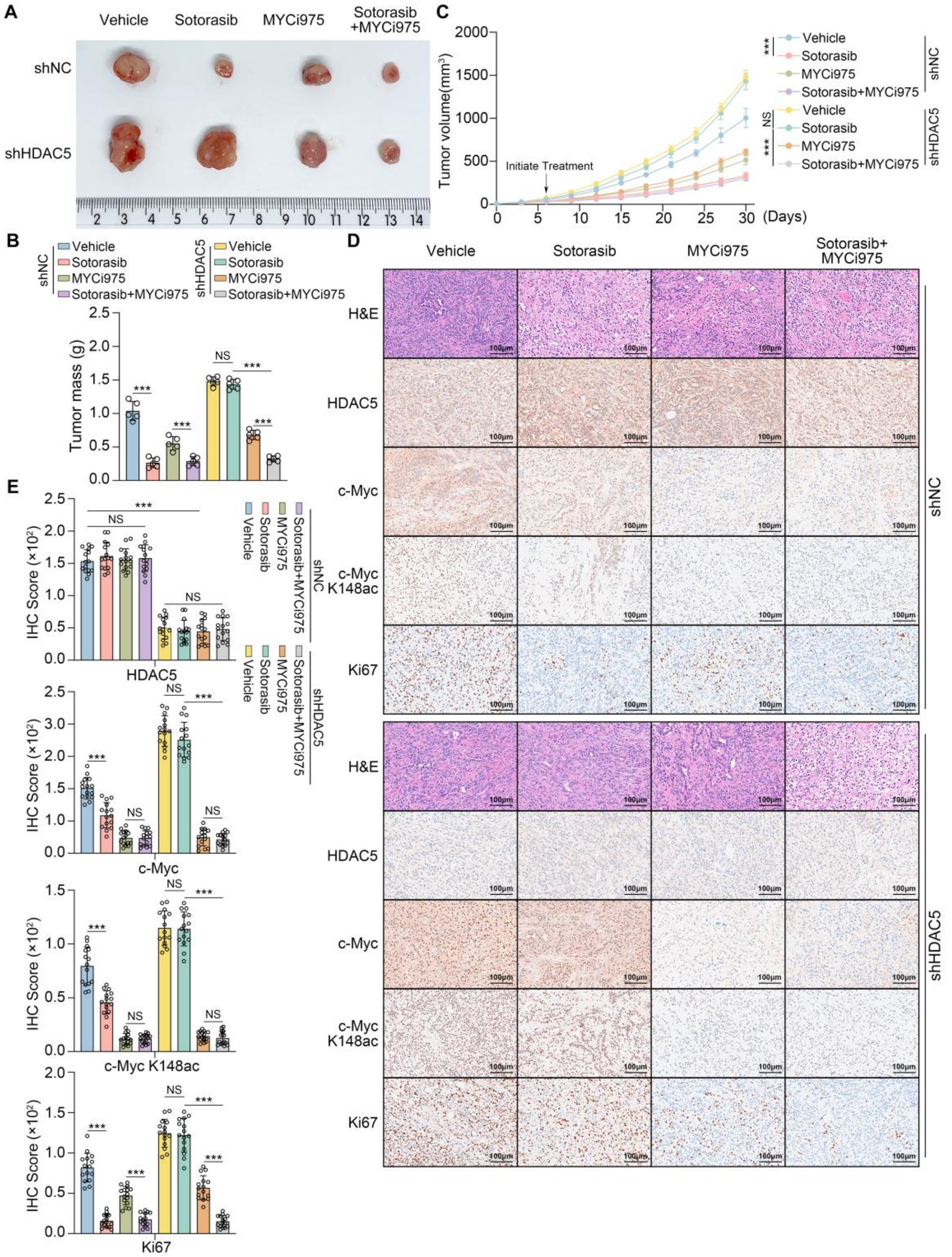

Supplemental Figure 22. Pharmacological inhibition of c-Myc overcomes resistance to KRAS^{G12C} inhibitors induced by HDAC5 loss in vivo. Related to Figure 9. (A) Representative images of subcutaneous tumor models treated with vehicle, Sotorasib (3 mg/kg, p.o., q.d.), MYCi975 (50 mg/kg, i.p., bid), or their combination, collected and photographed at day 30 post-euthanasia. **(B)** Weight of tumors harvested from subcutaneous models (n = 5). **(C)** Tumor volumes over time in

subcutaneous models (n = 5). **(D** and **E)** Representative IHC images of subcutaneous tumor models. IHC scores were quantified in **(E)**. Scale bars = 100 μm. n=5 biologically independent repeats and 3 independent IHC quantifications. All data are presented as the mean ± SD. Statistical significance was determined by two-way ANOVA followed by Tukey multiple comparisons test **(B, C** and **E)**. NS, not significant; **P* < 0.05, ***P* < 0.01, ****P* < 0.001.

Supplemental Table 1.
Sequence of primers and gene specific shRNAs, siRNAs and gRNAs

Gene	Usage	Forward	Reverse
GAPDH	RT-qPCR	ACCCAGAAGACTGTGGATGG	TTCAGCTCAGGGATGACCTT
HDAC5	RT-qPCR	TCTTGTCGAAGTCAAAGGAGC	GAGGGGAACTCTGGTCCAAAG
MYC	RT-qPCR	CTCCACACATCAGCACAACTA	TGTCCAACTTGACCCTCTTG
NEDD4	RT-qPCR	TCAGGACAACCTAACAGATGCT	TTCTGCAAGATGAGTTGGAACAT
MAP4K4	RT-qPCR	GACTCCCCTGCAAAAAGTCTG	GTCCATAGGTGCCATTTCCAA
MAPK7	RT-qPCR	GGTGACTTTGGTATGGCTCGT	CCAGAGGTCAATAGCCTGTGTA
MRAS	RT-qPCR	TTCCTCATCGTCTACTCCGTC	AGGATCATCGGGAATGACTCC
PAK1	RT-qPCR	CAGCCCCTCCGATGAGAAATA	CAAAACCGACATGAATTGTGTGT
MYC	Chip-qPCR	CAGGCAGACACATCTCAGGG	CCCATTGCATTTGTTGGGGG
MAP4K4	Chip-qPCR	GCAGCGCCAGACAATTACAG	CCACTCTCCCACACCCTAGA
MAPK7	Chip-qPCR	TCCCGCCCTTCCAGAATAGA	GATGGTCGGGGCATAGTCAG
MRAS	Chip-qPCR	CCAGGCTAGAGTGCAGTAGC	CAAAAGTCAACCCGGCATGG
PAK1	Chip-qPCR	ACGAGCTCATAGATGCGGTG	GTTGTTTCCGTGCAGCCTTT
**SiRNAs**	**Sequence**		
SiControl	UUCUCCGAACGUGUCACGUTT		
SiHDAC1	CAGCGAUGACUACAUUAAAUU		
SiHDAC2	GGUCAAUAAGACCAGAUAACA		
SiHDAC3	GGUAGUGGACUUCUACCAACC		
SiHDAC4	GCACAGAAGUGAAGAUGAAGU		
SiHDAC5	GGACUGGGACAUUCACCAUTT		
SiHDAC7	GCAAGAUCCUCAUUGUAGACU		
SiSIRT1	GACUCAAGUUCACCAGAAAGA		
SiSIRT2#1	GCGCGUUUCUUCUCCUGUAUG		
SiSIRT2#2	CGGCCUCUAUGACAACCUAGA		
SiNEDD4#1	CCAAGAAGUCACAAAUCAATT		
SiNEDD4#2	GGAUGUUCCAACUCAUCUUTT		
SiSTUB1#1	GGAGAUGGAGAGCUAUGAUTT		

SiSTUB1#2	CCAAGCACGACAAGUACAUTT
SiTRIM33#1	GGUAUGUACUAGUUGUGAATT
SiTRIM33#2	GGAAAGACAGAUGAAGUUATT
**shRNAs**	**Sequence**
sh-HDAC5#1	GCGGAACAAGGAGAAGAGCAA
sh-HDAC5#2	GCAGAAGTTGAACGTGGGCAA
shHdac5	GACGCCTCCCTCCTACAAATT
shMYC	CCTGAGACAGATCAGCAACAA
shMyc	TGGAGATGATGACCGAGTTAC
**gRNAs**	**Sequence**
gMYC#1	GCCGTATTTCTACTGCGACG
gMYC#2	GGCCGACCAGCTGGAGATGG
gMyc	TCGGGCTCATCTCCATCCCGAGG
gMYC (K148R)	GTCTCAGAGAGGCTGGCCTC
gMYC (K148Q)	TCTCAGAGCAGCTGGCCTCC

Supplemental Table 2. Key Resources

Reagent or Resource	Source	Identifier
**Antibodies**		
Rabbit polyclonal anti-GAPDH	Proteintech	Cat# 10494-1-AP<br>RRID: AB_2263076
Rabbit polyclonal anti-HDAC5	Proteintech	Cat# 16166-1-AP<br>RRID: AB_2116779
Rabbit polyclonal anti-Ki67	Proteintech	Cat# 27309-1-AP<br>RRID: AB_2756525
Rabbit polyclonal anti-c-MYC	Proteintech	Cat# 10828-1-AP<br>RRID: AB_2148585
Mouse monoclonal anti-HDAC5	Proteintech	Cat# 68437-1-Ig<br>RRID: AB_3085150
Mouse monoclonal anti-c-MYC	Proteintech	Cat# 67447-1-Ig<br>RRID: AB_2882681
Rabbit polyclonal anti-NEDD4	Proteintech	Cat# 21698-1-AP<br>RRID: AB_10858626
Rabbit polyclonal anti-STUB1	Proteintech	Cat# 55430-1-AP<br>RRID: AB_10949225
Rabbit polyclonal anti-TRIM33	Proteintech	Cat# 55374-1-AP<br>RRID: AB_11183035
Rabbit polyclonal anti-MAP4K4	Proteintech	Cat# 55247-1-AP<br>RRID: AB_10836939
Rabbit polyclonal anti-MRAS	Proteintech	Cat# 14213-1-AP<br>RRID: AB_10950895
Rabbit polyclonal anti-PAK1	Proteintech	Cat# 21401-1-AP<br>RRID: AB_11232232
Rabbit polyclonal anti-ubiquitin	Proteintech	Cat# 10201-2-AP<br>RRID: AB_671515
Rabbit polyclonal anti- HA tag	Proteintech	Cat# 51064-2-AP<br>RRID: AB_11042321
Rabbit polyclonal anti- Flag tag	Proteintech	Cat# 20543-1-AP<br>RRID: AB_11232216
Mouse monoclonal anti- Flag tag	Proteintech	Cat# 66008-4-Ig<br>RRID: AB_2918475
p44/42 MAPK (Erk1/2) (L34F12)	Cell Signaling Technology	Cat# 4696<br>RRID: AB_390780
Phospho-p44/42 MAPK (Erk1/2) (Thr202/Tyr204) (D13.14.4E)	Cell Signaling Technology	Cat# 4370<br>RRID: AB_2315112
Rabbit polyclonal anti-Acetylated Lysine	Cell Signaling Technology	Cat# 9441<br>RRID: AB_331805

Histone H3 Polyclonal antibody	Proteintech	Cat#17168-1-AP<br>RRID: AB_2716755
Rabbit polyclonal anti-HDAC1	Proteintech	Cat#10197-1-AP<br>RRID: AB_2118062
Rabbit polyclonal anti-HDAC2	Proteintech	Cat# 12922-3-AP<br>RRID: AB_2118516
HDAC3 Rabbit mAb	ABclonal	Cat# A19537<br>RRID: AB_2862654
HDAC4 Rabbit mAb	ABclonal	Cat# A13510<br>RRID: AB_2861688
HDAC7 Rabbit pAb	ABclonal	Cat# A7285<br>RRID: AB_2767826
SIRT1 Rabbit pAb	ABclonal	Cat# A11267<br>RRID: AB_2861537
SIRT2 Rabbit pAb	ABclonal	Cat# A0273<br>RRID: AB_2757086
ATF2 Rabbit pAb	ABclonal	Cat# A0757<br>RRID: AB_2757383
Phospho-ATF2-T71 Rabbit mAb	ABclonal	Cat# AP1051<br>RRID: AB_2863924
Acetyl-Histone H3-K9 Rabbit mAb	ABclonal	Cat# A21107<br>RRID: AB_3714809
cetyl-Histone H3-K27 Rabbit mAb	ABclonal	Cat# A22264<br>RRID: AB_3698464
Acetyl-p53- K370 Rabbit mAb	ABclonal	Cat# A19836<br>RRID: AB_2862752
Acetyl-p53-K382 Rabbit pAb	ABclonal	Cat# A18663<br>RRID: AB_2862400
Rabbit polyclonal anti-MAPK7	Cell Signaling Technology	Cat# ab264300<br>RRID: AB_330491
HRP-conjugated Goat Anti-Rabbit IgG(H+L)	Proteintech	Cat# SA00001-2<br>RRID: AB_2722564
HRP-conjugated Goat Anti-Mouse IgG(H+L)	Proteintech	Cat# SA00001-1<br>RRID: AB_2722565
Cy3–conjugated Goat Anti-Rabbit IgG(H+L)	Proteintech	Cat# SA00009-2<br>RRID: AB_2890957
Fluorescein (FITC)–conjugated Goat Anti-Mouse IgG(H+L)	Proteintech	Cat# SA00003-1<br>RRID: AB_2890896

VeriBlot IP Detection Reagent (HRP)	Abcam	Cat# ab131366<br>RRID: AB_2892718
Rabbit IgG	Beyotime	Cat# A7016<br>RRID: AB_2905533
Mouse IgG	Beyotime	Cat# A7028<br>RRID: AB_2909433
**Chemicals**		
MRTX1133	Selleck	E1051
MYCi975	Selleck	S8906
Cycloheximide	Selleck	S7418
MG132	Selleck	S2619
Sotorasib	Selleck	S8830
HRS-4642	Selleck	E4651
RMC-9805	Selleck	E1962
FDA-approved Anticancer Drug Library	Selleck	L8000
TH-Z835	Medchemexpress	HY-146243
GFH-375	GenFleet	N/A
MS-275	Selleck	S1053
EX-527	Selleck	S1541
AGK2	Selleck	S7577
LMK-235	Selleck	S7569
10058-F4	Selleck	S7153
MYCMI-6	Selleck	S0987
A-485	Medchemexpress	HY-107455
L-45	Medchemexpress	HY-101125
Puromycin Dihydrochloride	Beyotime	ST551
Polybrene	Beyotime	C0351
**Bacterial and virus strains**		
DH5a Competent E. coli	Tsingke	Cat#TSC-C14
BL21 (DE3) Competent E. coli	Tsingke	Cat#TSC-E01
**Recombinant DNA**		
Flag-HDAC5	Genechem	N/A
Flag-HDAC5(H833A)	Genechem	N/A
HA-NEDD4	Genechem	N/A
Flag-c-Myc (WT)	Genechem	N/A
Flag-c-Myc(K148R)	Genechem	N/A
Flag-c-Myc(K148Q)	Genechem	N/A
HA-Ub	OBIO	N/A
**Critical commercial assays**		
ChIP Kit Magnetic-One Step	Abcam	ab156907
Dual-Luciferase Reporter Assay System	Promega	E1980

Cell Counting Kit-8	HYCEZMBIO	HYCCK8
**Cell lines**		
HEK293T	Procell	Cat# CL-0005
Mia PaCa-2	Procell	Cat# CL-0627
PANC-1	Procell	Cat#CL-0184
AsPC-1	Procell	Cat#CL-0027
KPC-Luc	Model Organisms Center, Inc..	Cat# NM-YD04-TG01
**Software and algorithms**		
Graphpad Prism 9.5	Graphpad software	https://www.graphpad.com/
ImageJ	ImageJ: Image Processing and	https://imagej.nih.gov/ij/
