# Supplementary material for: HDAC5 deficiency induces intrinsic resistance to KRAS inhibition by disrupting c-Myc acetylation-ubiquitination homeostasis
Source: J Clin Invest. 2025 Dec 11;136(3):e195814. doi: 10.1172/JCI195814 (PMC12867140; doi:10.1172/JCI195814)
Supplement: Unedited blot and gel images [file jci-136-195814-s048.pdf]

Figure 3H

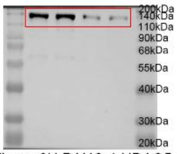

Figure 3H PANC-1 HDAC5

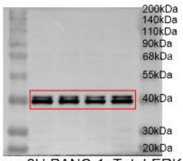

Figure 3H PANC-1 Total-ERK

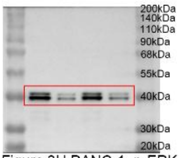

Figure 3H PANC-1 p-ERK

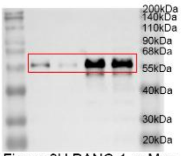

Figure 3H PANC-1 c-Myc

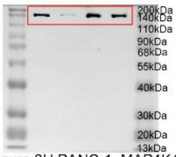

Figure 3H PANC-1 MAP4K4

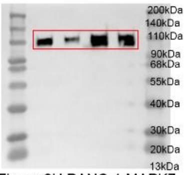

Figure 3H PANC-1 MAPK7

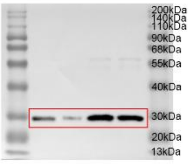

Figure 3H PANC-1 MRAS

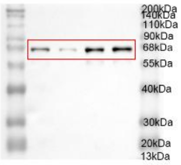

Figure 3H PANC-1 PAK1

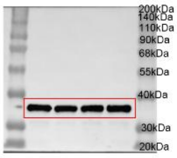

Figure 3H PANC-1 GAPDH

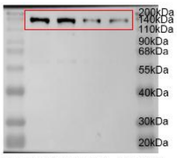

Figure 3H AsPC-1 HDAC5

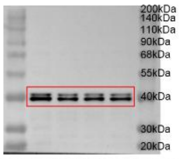

Figure 3H AsPC-1 Total-ERK

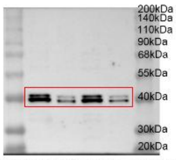

Figure 3H AsPC-1 p-ERK

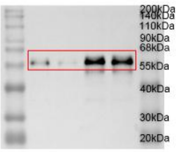

Figure 3H AsPC-1 c-Myc

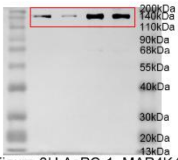

Figure 3H AsPC-1 MAP4K4

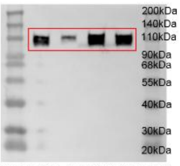

Figure 3H AsPC-1 MAPK7

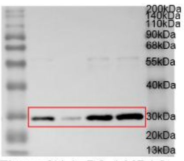

Figure 3H AsPC-1 MRAS

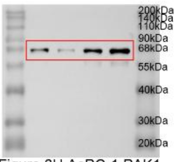

Figure 3H AsPC-1 PAK1

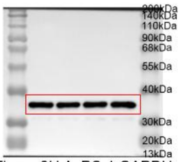

Figure 3H AsPC-1 GAPDH

Figure 3I

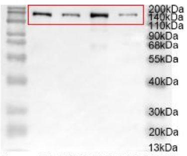

Figure 3I PANC-1 MAP4K4

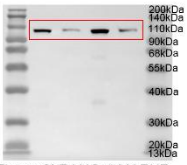

Figure 3I PANC-1 MAPK7

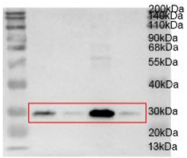

Figure 3I PANC-1 MRAS

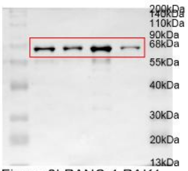

Figure 3I PANC-1 PAK1

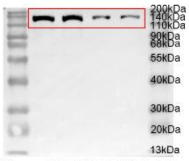

Figure 3I PANC-1 HDAC5

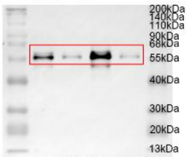

Figure 3I PANC-1 c-Myc

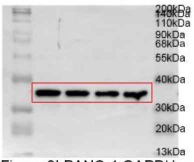

Figure 3I PANC-1 GAPDH

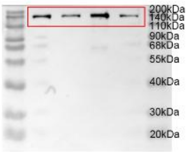

Figure 3I AsPC-1MAP4K4

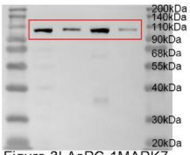

Figure 3I AsPC-1MAPK7

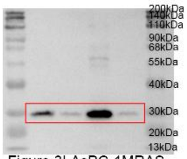

Figure 3I AsPC-1MRAS

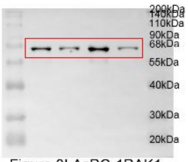

Figure 3I AsPC-1PAK1

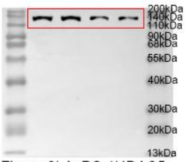

Figure 3I AsPC-1HDAC5

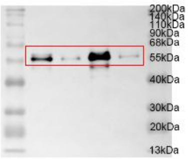

Figure 3I AsPC-1c-Myc

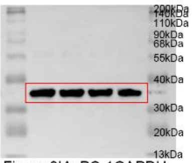

Figure 3IAsPC-1GAPDH

Figure 4A

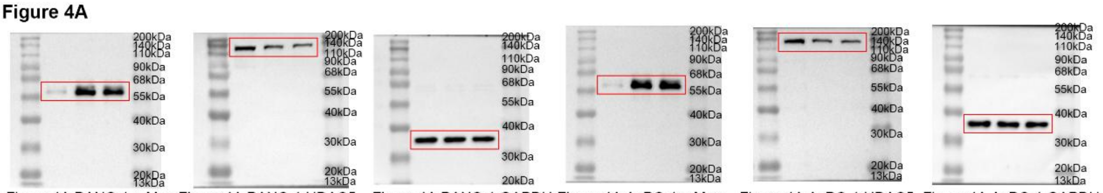

Figure 4A PANC-1 c-Myc Figure 4A PANC-1 HDAC5 Figure 4A PANC-1 GAPDH Figure 4A AsPC-1 c-Myc Figure 4A AsPC-1 HDAC5 Figure 4A AsPC-1 GAPDH

Figure 4C

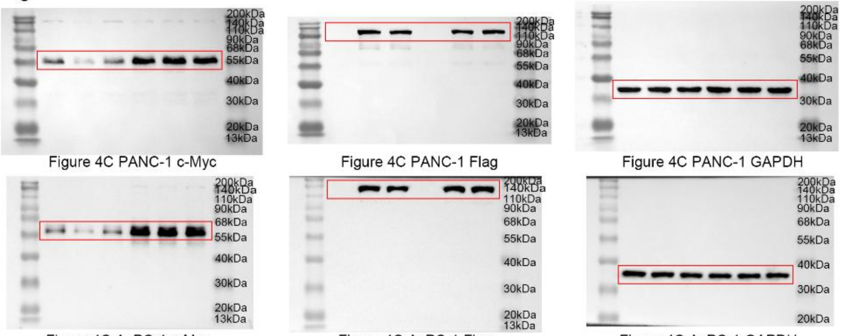

Figure 4C PANC-1 c-Myc Figure 4C PANC-1 Flag Figure 4C PANC-1 GAPDH

Figure 4C AsPC-1 c-Myc Figure 4C AsPC-1 Flag Figure 4C AsPC-1 GAPDH

Figure 4F

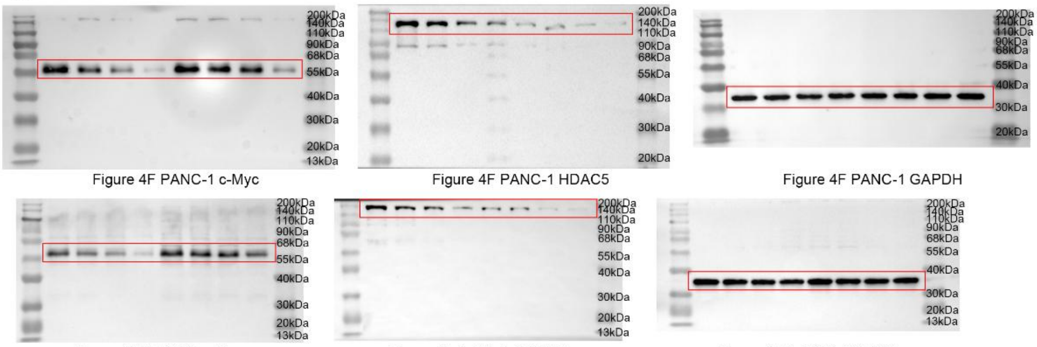

Figure 4F PANC-1 c-Myc Figure 4F PANC-1 HDAC5 Figure 4F PANC-1 GAPDH

Figure 4F AsPC-1 c-Myc Figure 4F AsPC-1 HDAC5 Figure 4F AsPC-1 GAPDH

Figure 5A

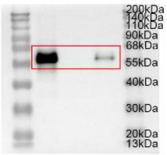

Figure 5A PANC-1 c-Myc

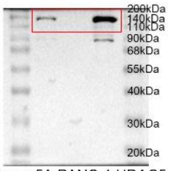

Figure 5A PANC-1 HDAC5

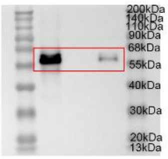

Figure 5A AsPC-1 c-Myc

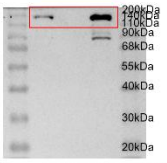

Figure 5A AsPC-1 HDAC5

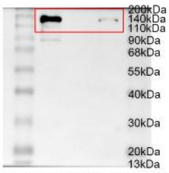

Figure 5A PANC-1 HDAC5

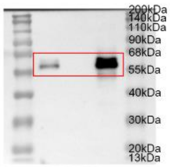

Figure 5A PANC-1 c-Myc

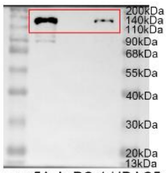

Figure 5A AsPC-1 HDAC5

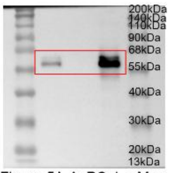

Figure 5A AsPC-1 c-Myc

Figure 5C

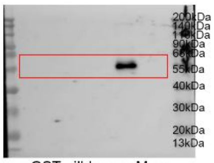

GST pilldown c-Myc

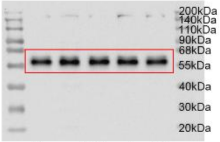

Figure 5C Input-c-Myc

Figure 5D

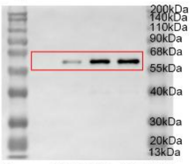

Figure 5D PANC-1 IP Ack

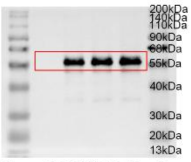

Figure 5D PANC-1 IP c-Myc

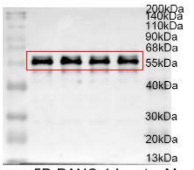

Figure 5D PANC-1 Input c-Myc

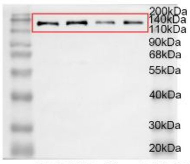

Figure 5D PANC-1 Input HDAC5

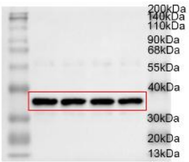

Figure 5D PANC-1 Input GAPDH

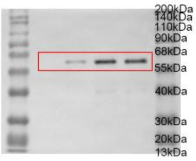

Figure 5D AsPC-1 IP Ack

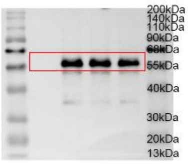

Figure 5D AsPC-1 IP c-Myc

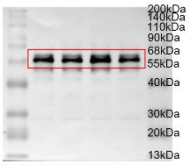

Figure 5D AsPC-1 Input c-Myc

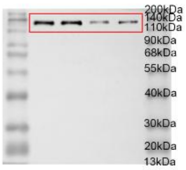

Figure 5D AsPC-1 Input HDAC5

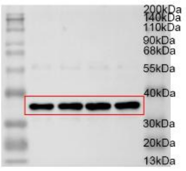

Figure 5D AsPC-1 Input GAPDH

Figure 5E

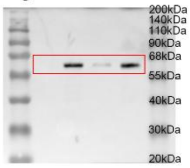

Figure 5E PANC-1 IP Ack

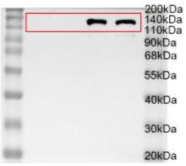

Figure 5E PANC-1 IP Flag

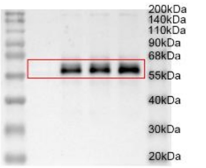

Figure 5E PANC-1 IP c-Myc

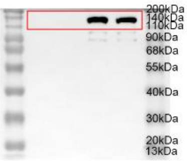

Figure 5E PANC-1 Input Flag

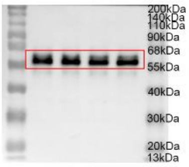

Figure 5E PANC-1 Input c-Myc

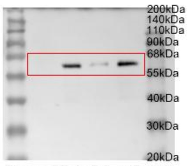

Figure 5E AsPC-1 IP Ack

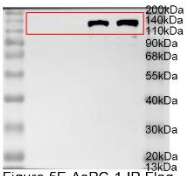

Figure 5E AsPC-1 IP Flag

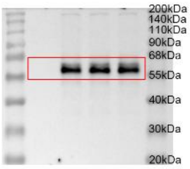

Figure 5E AsPC-1 IP c-Myc

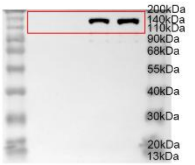

Figure 5E AsPC-1 Input Flag

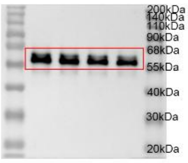

Figure 5E AsPC-1 Input c-Myc

Figure 5F

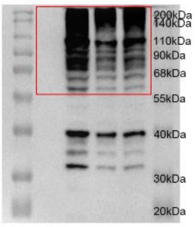

Figure 5F PANC-1IB:HA

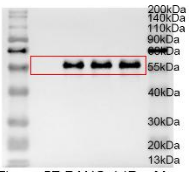

Figure 5F PANC-1 IP c-Myc

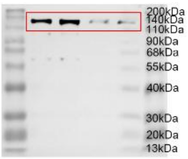

Figure 5F PANC-1 Input HDAC5

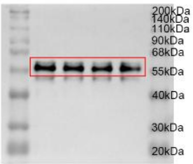

Figure 5F PANC-1 Input c-Myc

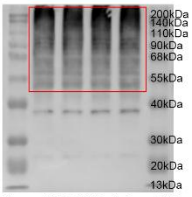

Figure 5F PANC-1 Input HA

Figure 5G

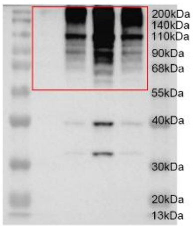

Figure 5G PANC-1IB:HA

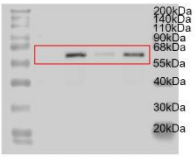

Figure 5G PANC-1IP AcK

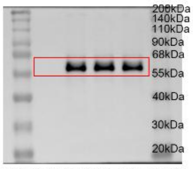

Figure 5G PANC-1IP c-Myc

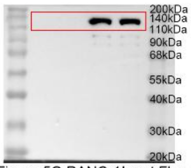

Figure 5G PANC-1Input Flag

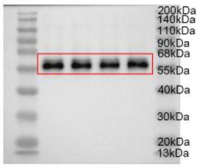

Figure 5G PANC-1Input c-Myc

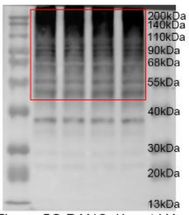

Figure 5G PANC-1Input HA

Figure 6C

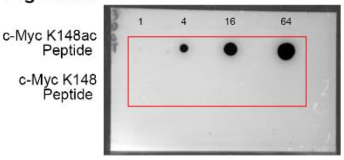

Figure 6D

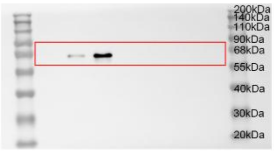

Figure 6D IP c-Myc-K148-ac

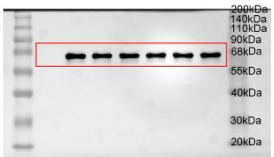

Figure 6D IP Flag

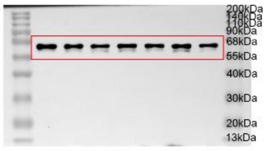

Figure 6D Input Flag

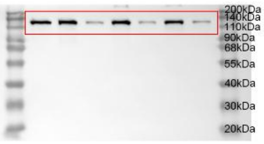

Figure 6D Input HDAC5

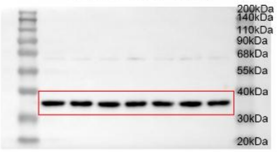

Figure 6D Input GAPDH

Figure 6F

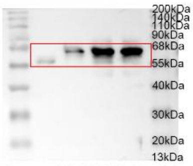

Figure 6F PANC-1 c-Myc

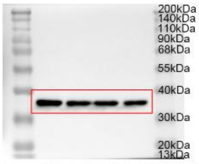

Figure 6F PANC-1 GAPDH

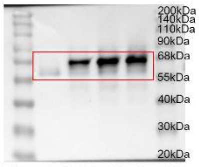

Figure 6F AsPC-1 c-Myc

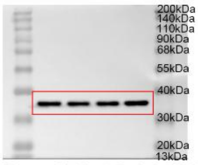

Figure 6F AsPC-1 GAPDH

Figure 6H

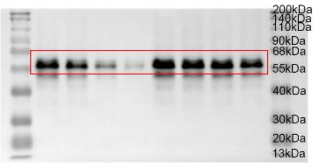

Figure 6H Flag-K148R c-Myc

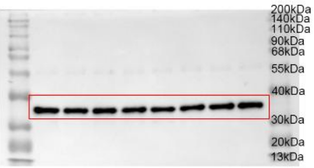

Figure 6H Flag-K148R GAPDH

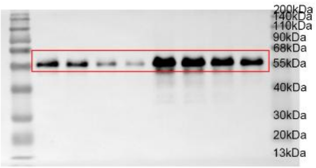

Figure 6H Flag-K148Q c-Myc

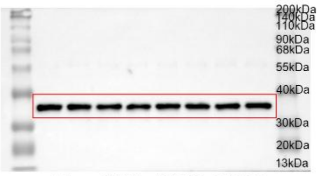

Figure 6H Flag-K148Q GAPDH

Figure 6I

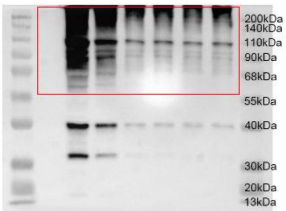

Figure 6I IB:HA

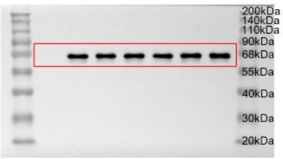

Figure 6I IP Flag

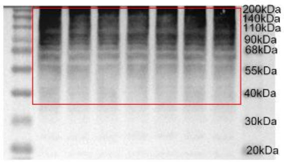

Figure 6I Input HA

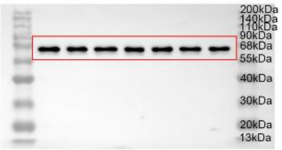

Figure 6I Input Flag

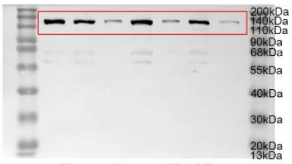

Figure 6I Input HDAC5

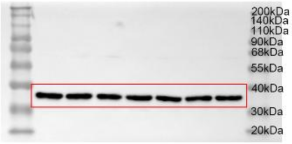

Figure 6I Input GAPDH

Figure 7B

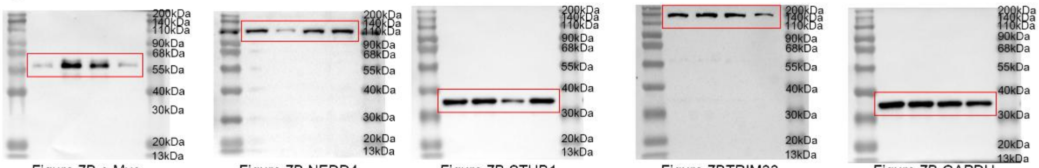

Figure 7B c-Myc Figure 7B NEDD4 Figure 7B STUB1 Figure 7BTRIM33 Figure 7B GAPDH

Figure 7C

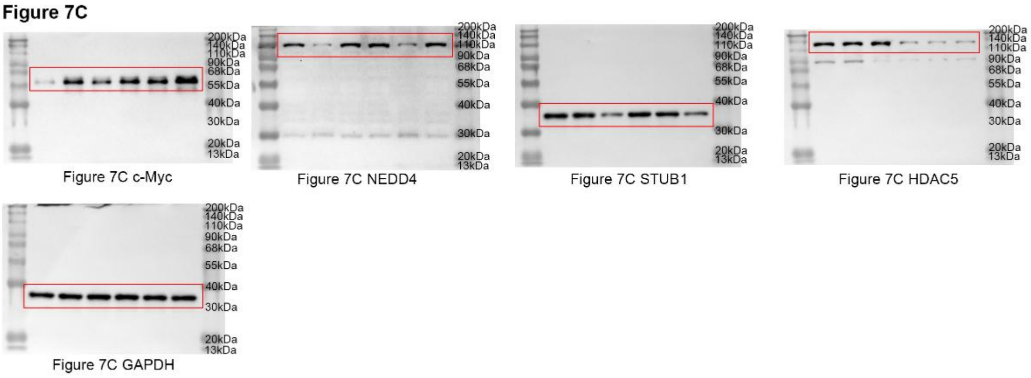

Figure 7C c-Myc Figure 7C NEDD4 Figure 7C STUB1 Figure 7C HDAC5

Figure 7C GAPDH

Figure 7D

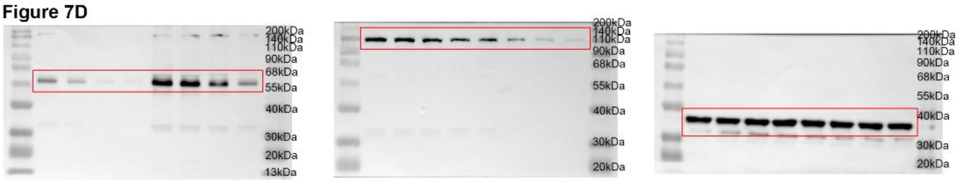

Figure 7D c-Myc Figure 7D NEDD4 Figure 7D GAPDH

Figure 7E

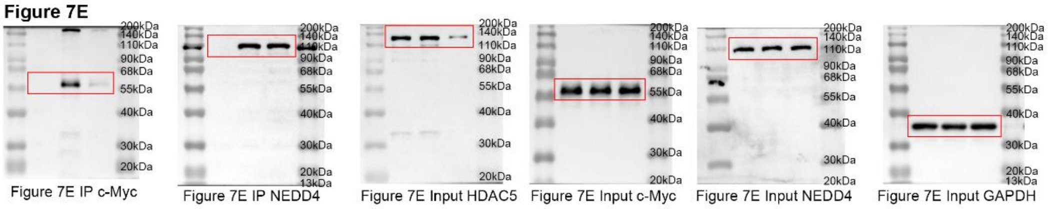

Figure 7E IP c-Myc Figure 7E IP NEDD4 Figure 7E Input HDAC5 Figure 7E Input c-Myc Figure 7E Input NEDD4 Figure 7E Input GAPDH

Figure 7F

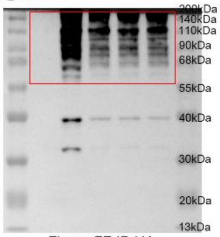

Figure 7F IB:HA

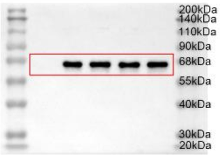

Figure 7F IP Flag

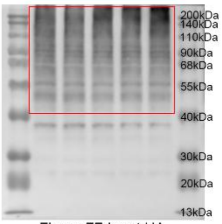

Figure 7F Input HA

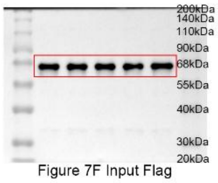

Figure 7F Input Flag

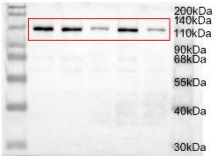

Figure 7F Input NEDD4

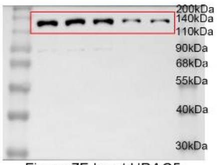

Figure 7F Input HDAC5

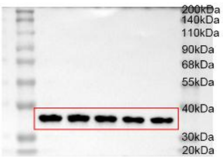

Figure 7F Input GAPDH

Figure 7G

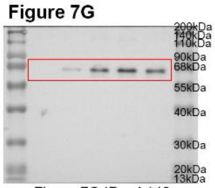

Figure 7G IP ack148

Figure 7G

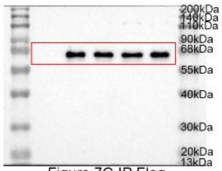

Figure 7G IP Flag

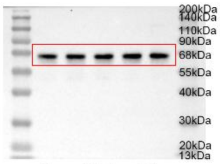

Figure 7G Input Flag

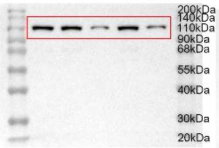

Figure 7G Input NEDD4

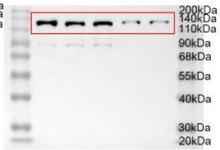

Figure 7G Input HDAC5

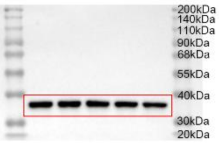

Figure 7G Input GAPDH

Figure 7H

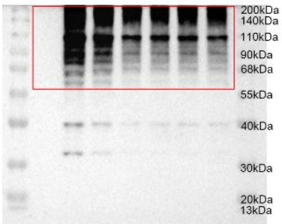

Figure 7H IB:HA

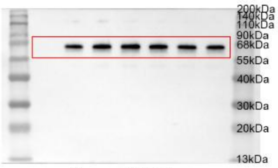

Figure 7H IP Flag

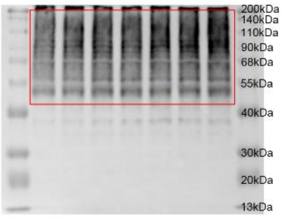

Figure 7H Input HA

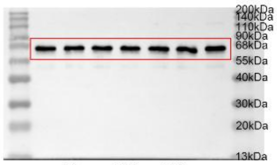

Figure 7H Input Flag

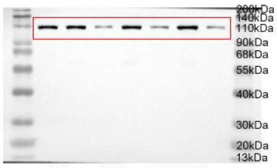

Figure 7H Input NEDD4

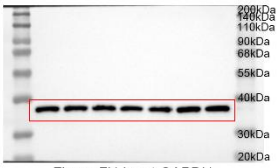

Figure 7H Input GAPDH

Figure 7I

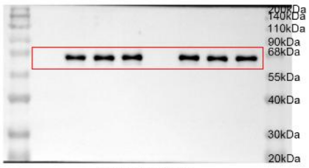

Figure 7I Flag

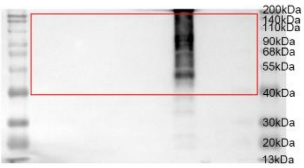

Figure 7I Ub

Figure 8G

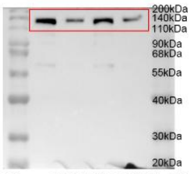

Figure 8G PANC-1 HDAC5

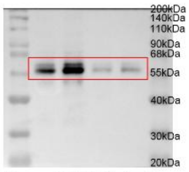

Figure 8G PANC-1 c-Myc

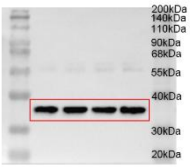

Figure 8G PANC-1 GAPDH

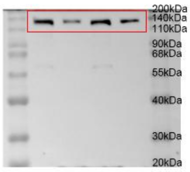

Figure 8G AsPC-1 HDAC5

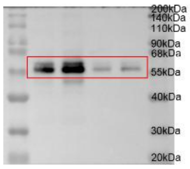

Figure 8G AsPC-1 c-Myc

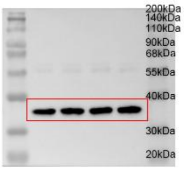

Figure 8G AsPC-1 GAPDH

Supplemental Figure 1A

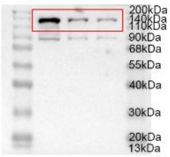

Figure S1A PANC-1 HDAC5

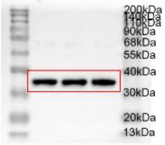

Figure S1A PANC-1 GAPDH

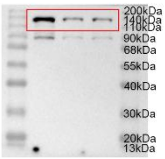

Figure S1A AsPC-1 HDAC5

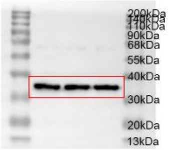

Figure S1A AsPC-1 GAPDH

Supplemental Figure 1D

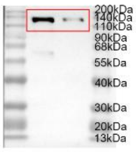

Figure S1D HDAC5

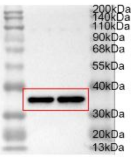

Figure S1D GAPDH

Supplemental Figure 1G

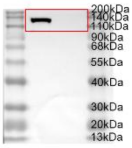

Figure S1G Hdac5

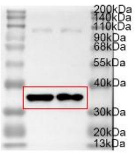

Figure S1G Gapdh

Supplemental Figure 1J

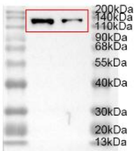

Figure S1J HDAC5

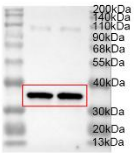

Figure S1J GAPDH

Supplemental Figure 3A

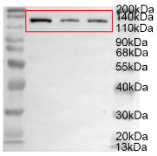

Figure S3A HDAC5

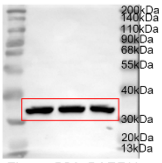

Figure S3A GAPDH

Supplemental Figure 6F

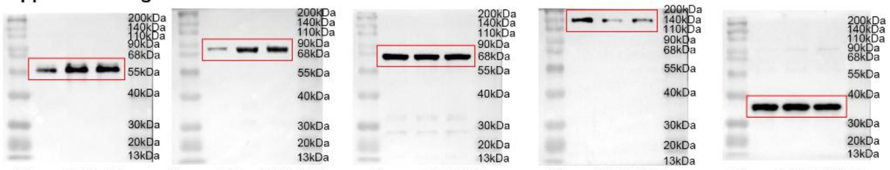

Figure S6F c-Myc   Figure S6F p-ATF2-T71   Figure S6F ATF2   Figure S6F HDAC5   Figure S6F GAPDH

Supplemental Figure 6G

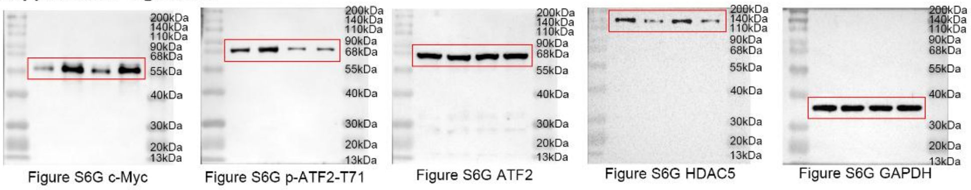

Figure S6G c-Myc   Figure S6G p-ATF2-T71   Figure S6G ATF2   Figure S6G HDAC5   Figure S6G GAPDH

Supplemental Figure 7C

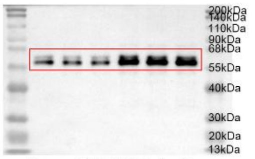

Figure S7C PANC-1 c-Myc

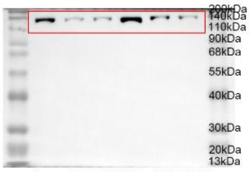

Figure S7C PANC-1 HDAC5

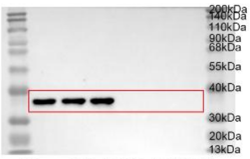

Figure S7C PANC-1 GAPDH

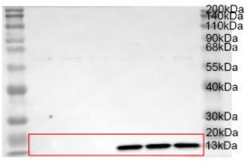

Figure S7C PANC-1 H3

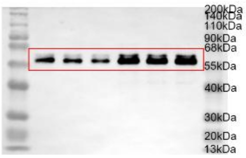

Figure S7C AsPC-1 c-Myc

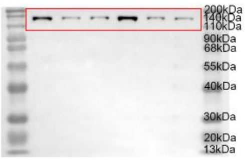

Figure S7C AsPC-1 HDAC5

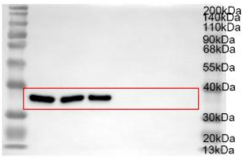

Figure S7C AsPC-1 GAPDH

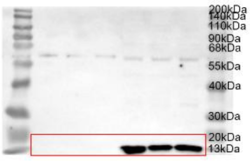

Figure S7C AsPC-1 H3

Supplemental Figure 7D

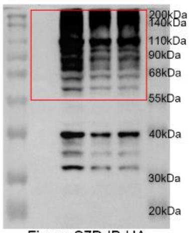

Figure S7D IB HA

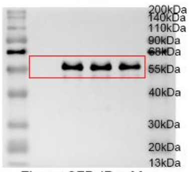

Figure S7D IP c-Myc

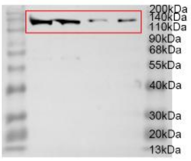

Figure S7D Input HDAC5

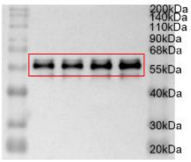

Figure S7D Input c-Myc

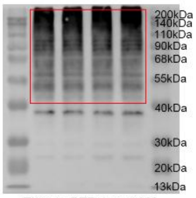

Figure S7D Input HA

Supplemental Figure 7E

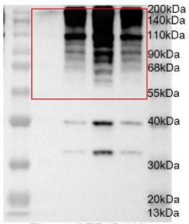

Figure S7E IB HA

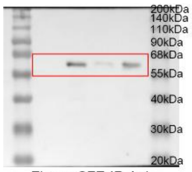

Figure S7E IP Ack

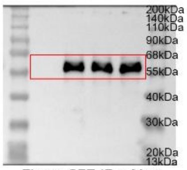

Figure S7E IP c-Myc

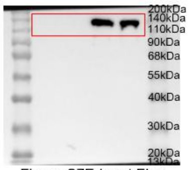

Figure S7E Input Flag

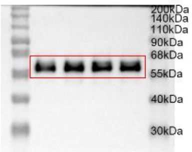

Figure S7E Input c-Myc

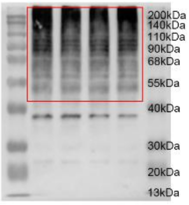

Figure S7E Input HA

Supplemental Figure 8B

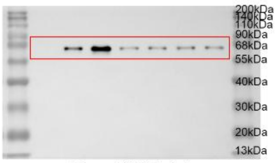

Figure S8B IP Ack

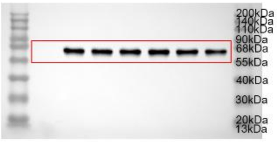

Figure S8B IP Flag

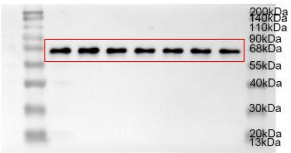

Figure S8B Input Flag

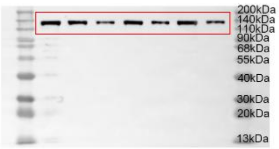

Figure S8B Input HDAC5

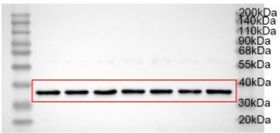

Figure S8B Input GAPDH

Supplemental Figure 8C

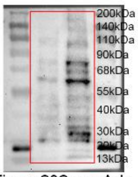

Figure S8C pan-Ack

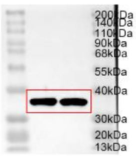

Figure S8C GAPDH

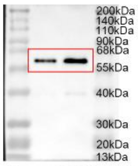

Figure S8C Ack148

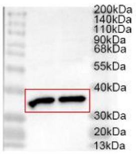

Figure S6C GAPDH

Supplemental Figure 8D

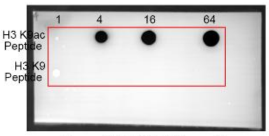

Figure S8D H3-K9ac mAb

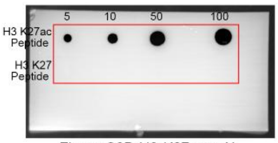

Figure S8D H3-K27ac mAb

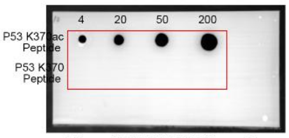

Figure S8D P53-K370ac mAb

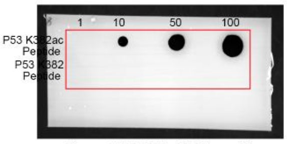

Figure S8D P53-K382ac pAb

Supplemental Figure 8E

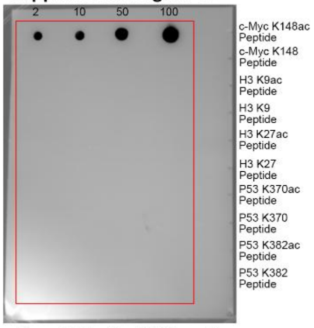

Figure S8E c-Myc-K148ac pAb

Supplemental Figure 9A

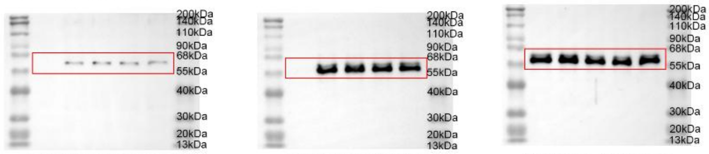

Figure S9A IP K148ac Figure S9A IP c-Myc Figure S9A Input c-Myc

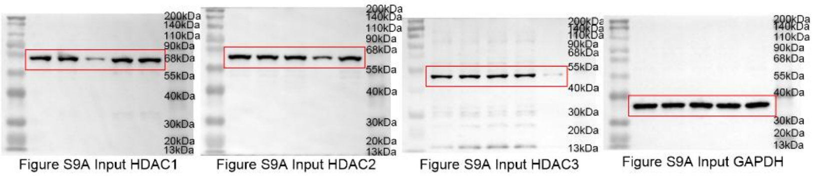

Figure S9A Input HDAC1 Figure S9A Input HDAC2 Figure S9A Input HDAC3 Figure S9A Input GAPDH

Supplemental Figure 9B

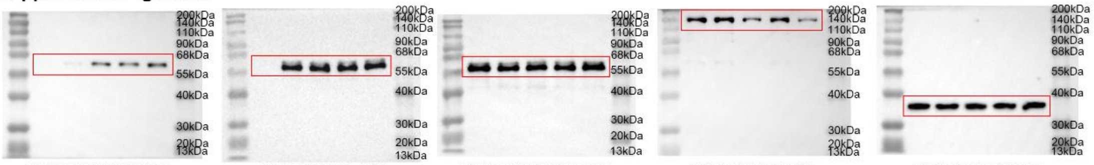

Figure S9B IP K148ac Figure S9B IP c-Myc Figure S9B Input c-Myc Figure S9B HDAC5 Figure S9B GAPDH

Supplemental Figure 9C

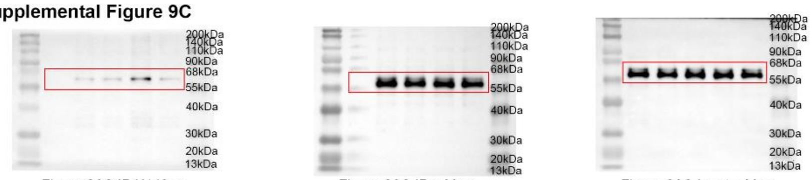

Figure S9C IP K148ac Figure S9C IP c-Myc Figure S9C Input c-Myc

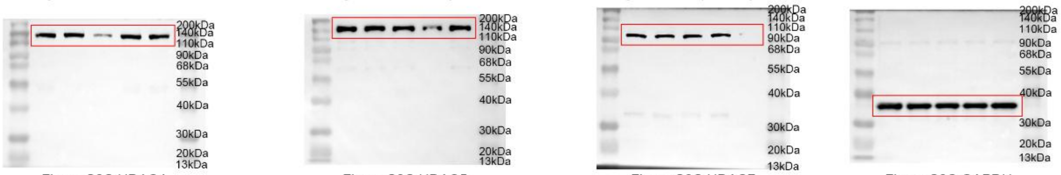

Figure S9C HDAC4 Figure S9C HDAC5 Figure S9C HDAC7 Figure S9C GAPDH

Supplemental Figure 9D

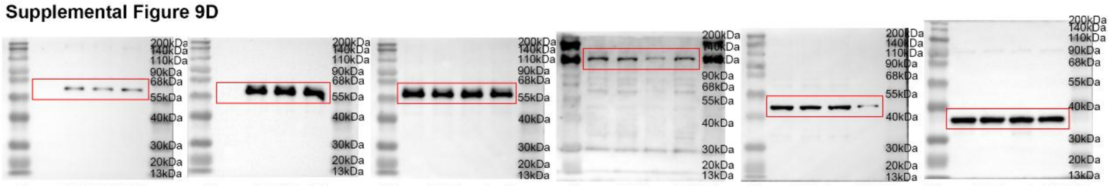

Figure S9D IP K148ac Figure S9D IP c-Myc Figure S9D Input c-Myc Figure S9D Input SIRT1 Figure S9D Input SIRT2 Figure S9D Input GAPDH

Supplemental Figure 9E

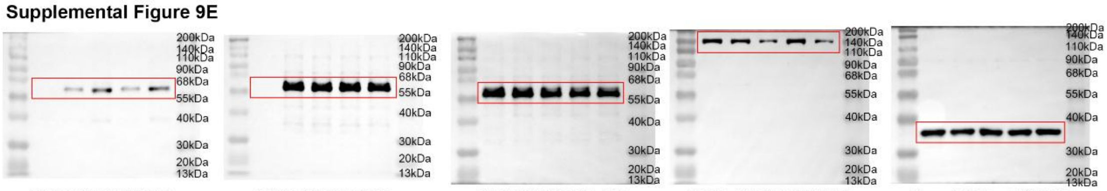

Figure S9E IP K148ac Figure S9E IP c-Myc Figure S9E Input c-Myc Figure S9E Input HDAC5 Figure S9E Input GAPDH

Supplemental Figure 9F

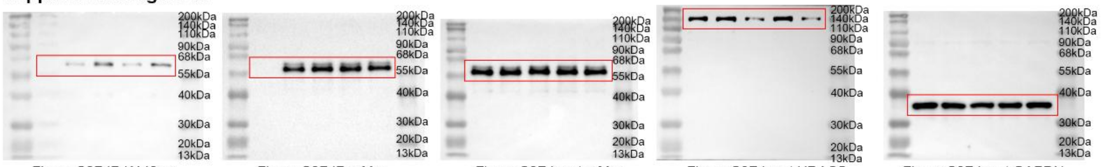

Figure S9F IP K148ac | Figure S9F IP c-Myc | Figure S9F Input c-Myc | Figure S9F Input HDAC5 | Figure S9F Input GAPDH

Supplemental Figure 9G

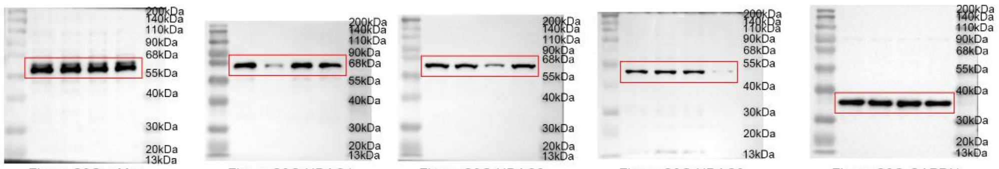

Figure S9G c-Myc | Figure S9G HDAC1 | Figure S9G HDAC2 | Figure S9G HDAC3 | Figure S9G GAPDH

Supplemental Figure 9H

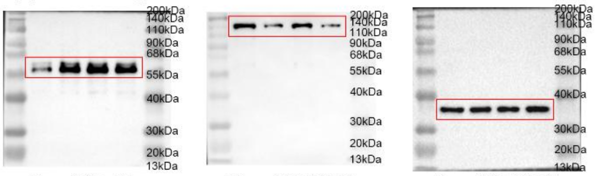

Figure S9H c-Myc | Figure S9H HDAC5 | Figure S9H GAPDH

Supplemental Figure 9I

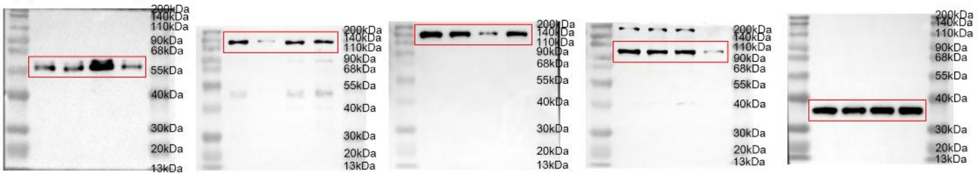

Figure S9I c-Myc | Figure S9I HDAC4 | Figure S9I HDAC5 | Figure S9I HDAC7 | Figure S9I GAPDH

Supplemental Figure 9J

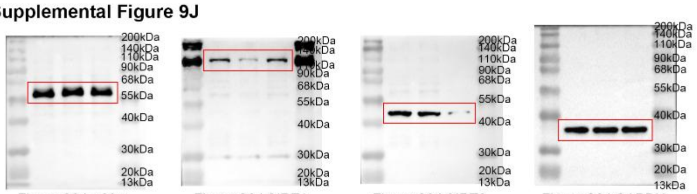

Figure S9J c-Myc | Figure S9J SIRT1 | Figure S9J SIRT2 | Figure S9J GAPDH

Supplemental Figure 9K

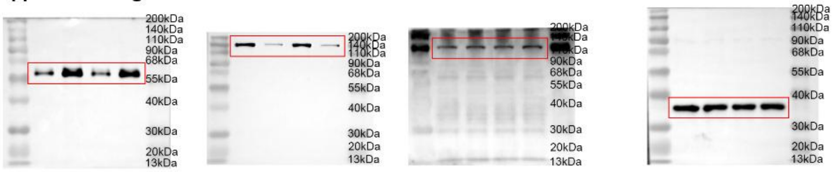

Figure S9K c-Myc | Figure S9K HDAC5 | Figure S9K SIRT1 | Figure S9K GAPDH

Supplemental Figure 9L

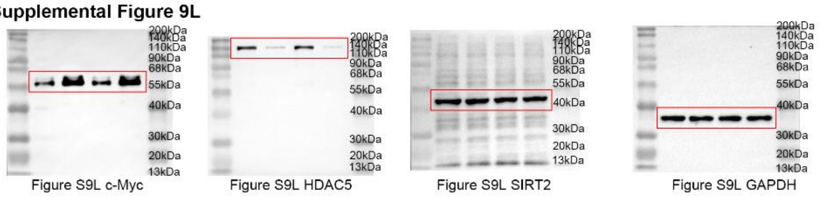

Figure S9L c-Myc | Figure S9L HDAC5 | Figure S9L SIRT2 | Figure S9L GAPDH

Supplemental Figure 9M

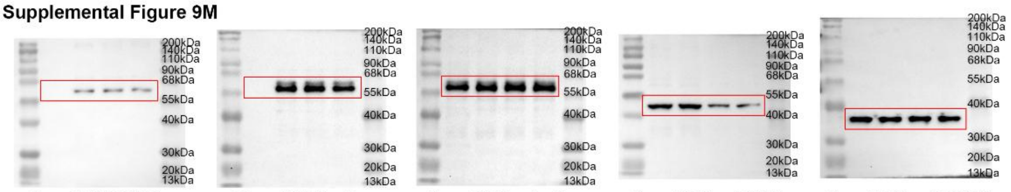

Figure S9M IP K148ac Figure S9M IP c-Myc Figure S9M Input c-Myc Figure S9M Input SIRT2 Figure S9M Input GAPDH

Supplemental Figure 9N

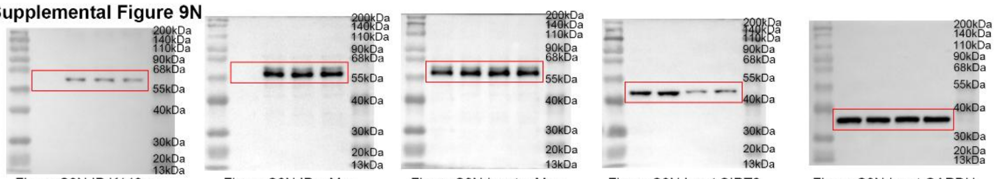

Figure S9N IP K148ac Figure S9N IP c-Myc Figure S9N Input c-Myc Figure S9N Input SIRT2 Figure S9N Input GAPDH

Supplemental Figure 9O

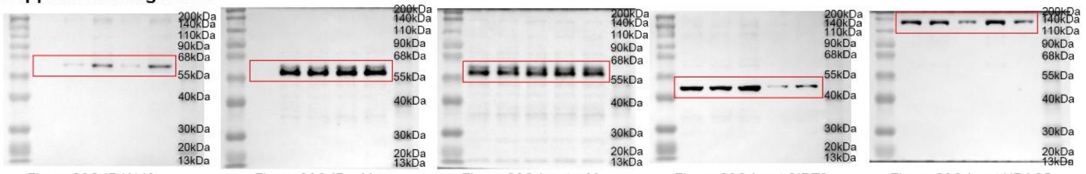

Figure S9O IP K148ac Figure S9O IP c-Myc Figure S9O Input c-Myc Figure S9O Input SIRT2 Figure S9O Input HDAC5

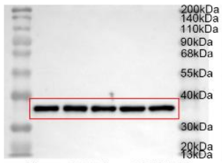

Figure S9O Input GAPDH

Supplemental Figure 9P

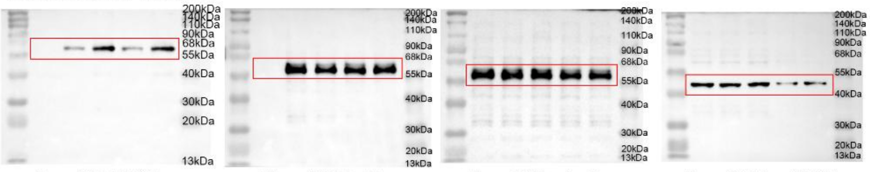

Figure S9P IP K148ac Figure S9P IP c-Myc Figure S9P Input c-Myc Figure S9P Input SIRT2

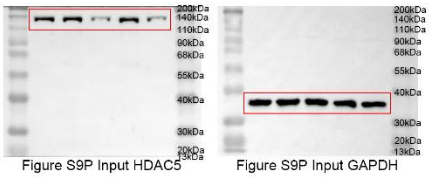

Figure S9P Input HDAC5 Figure S9P Input GAPDH

Supplemental Figure 9Q

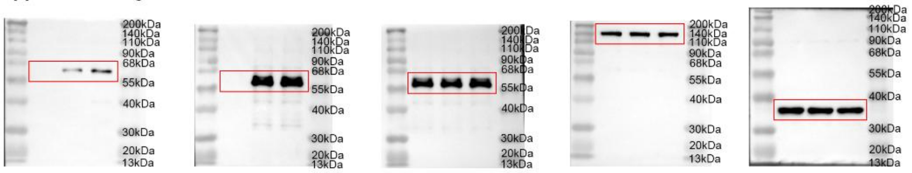

Figure S9Q IP K148ac Figure S9Q IP c-Myc Figure S9Q Input c-Myc Figure S9Q Input HDAC5 Figure S9Q Input GAPDH

Supplemental Figure 9R

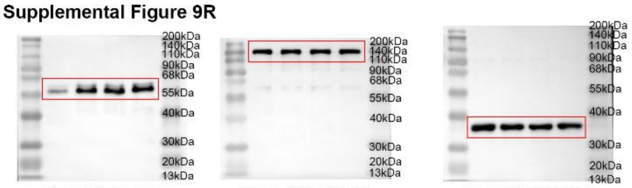

Figure S9R c-Myc Figure S9R HDAC5 Figure S9R GAPDH

Supplemental Figure 10C

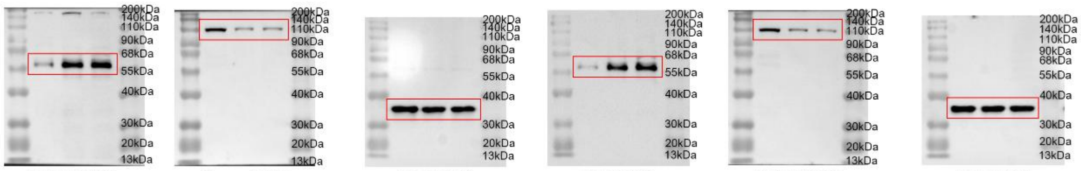

Figure S10C PANC-1 c-Myc | Figure S10C PANC-1 NEDD4 | Figure S10 PANC-1 GAPDH | Figure S10 AsPC-1 c-Myc | Figure S10C AsPC-1 NEDD4 | Figure S10 AsPC-1 GAPDH

Supplemental Figure 10D

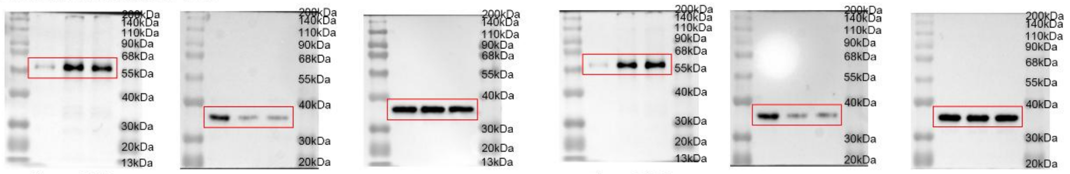

Figure S10D PANC-1 c-Myc | Figure S10 PANC-1 STUB1 | Figure S10D PANC-1 GAPDH | Figure S10D AsPC-1 c-Myc | Figure S10D AsPC-1 STUB1 | Figure S10D AsPC-1 GAPDH

Supplemental Figure 10E

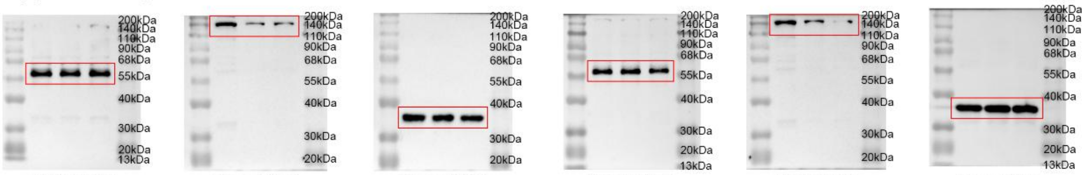

Figure S10E PANC-1 c-Myc | Figure S10E PANC-1 TRIM33 | Figure S10E PANC-1 GAPDH | Figure S10E AsPC-1 c-Myc | Figure S10E AsPC-1 TRIM33 | Figure S10E AsPC-1 GAPDH

Supplemental Figure 10F

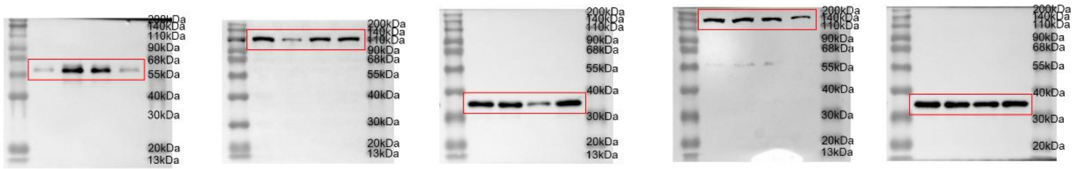

Figure S10F c-Myc | Figure S10F NEDD4 | Figure S10F STUB1 | Figure S10F TRIM33 | Figure S10F GAPDH

Supplemental Figure 10G

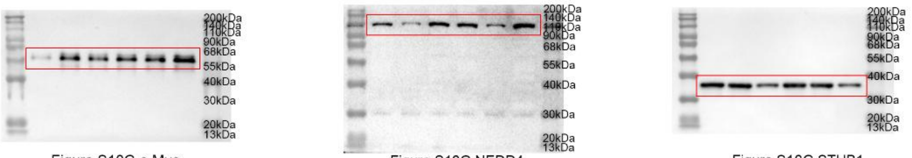

Figure S10G c-Myc | Figure S10G NEDD4 | Figure S10G STUB1

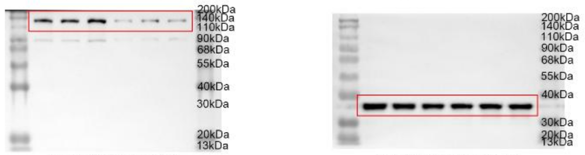

Figure S10G HDAC5 | Figure S10G GAPDH

Supplemental Figure 11A

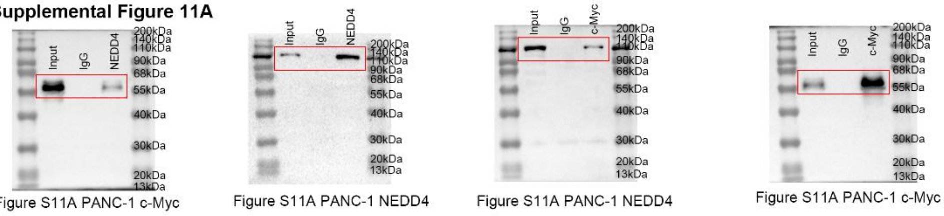

Figure S11A PANC-1 c-Myc
Figure S11A PANC-1 NEDD4
Figure S11A PANC-1 NEDD4
Figure S11A PANC-1 c-Myc

Supplemental Figure 11B

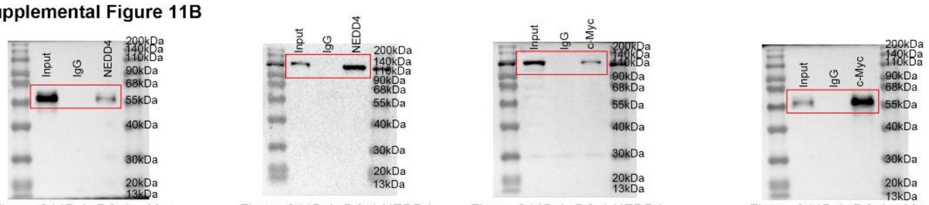

Figure S11B AsPC-1 c-Myc
Figure S11B AsPC-1 NEDD4
Figure S11B AsPC-1 NEDD4
Figure S11B AsPC-1 c-Myc

Supplemental Figure 11C

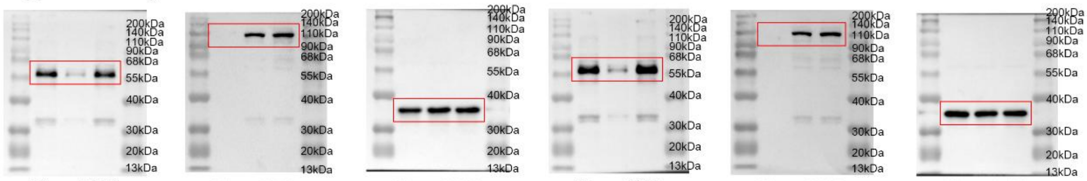

Figure S11C PANC-1 c-Myc
Figure S11C PANC-1 HA
Figure S11C PANC-1 GAPDH
Figure S11C AsPC-1 c-Myc
Figure S11C AsPC-1 HA
Figure S11C AsPC-1 GAPDH

Supplemental Figure 11E

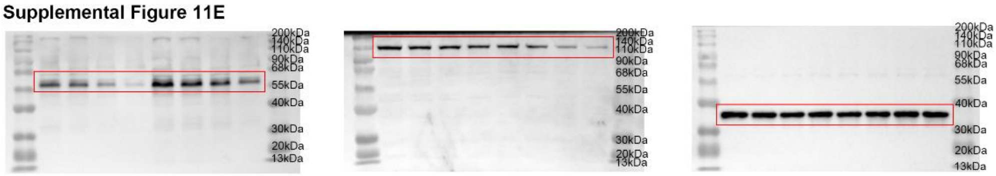

Figure S11E c-Myc
Figure S11E NEDD4
Figure S11E GAPDH

Supplemental Figure 11G

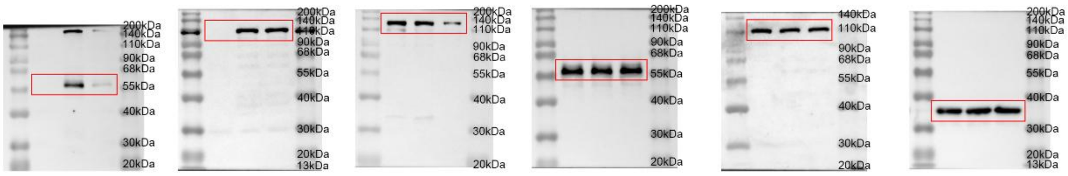

Figure S11G IP c-Myc
Figure S11G IP NEDD4
Figure S11G Input HDAC5
Figure S11G Input c-Myc
Figure S11G Input NEDD4
Figure S11G Input GAPDH

Supplemental Figure 11H

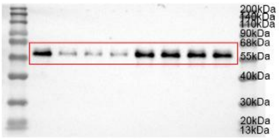

Figure S11H PANC-1 c-Myc

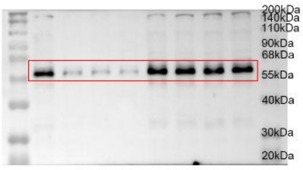

Figure S11H AsPC-1 c-Myc

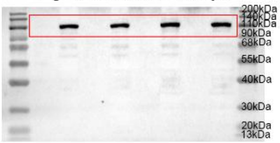

Figure S11H PANC-1 HA

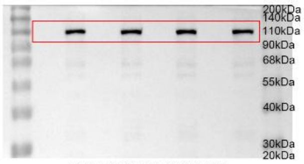

Figure S11H AsPC-1 HA

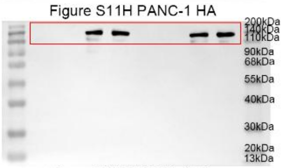

Figure S11H PANC-1 Flag

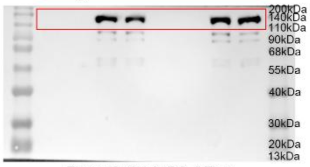

Figure S11H AsPC-1 Flag

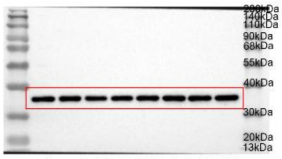

Figure S11H PANC-1 GAPDH

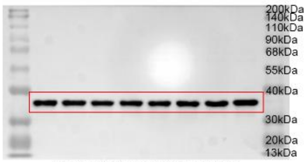

Figure S11H AsPC-1 GAPDH

Supplemental Figure 11I

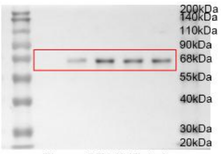

Figure S711I IP Ack

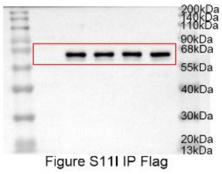

Figure S11I IP Flag

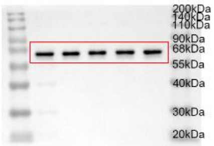

Figure S11I Input Flag

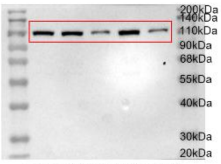

Figure S11I Input NEDD4

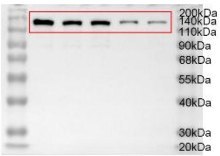

Figure S11I Input HDAC5

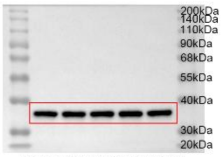

Figure S11I Input GAPDH

Supplemental Figure 12A

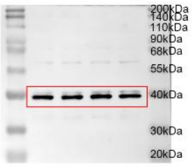

Figure S12A PANC-1 total-ERK

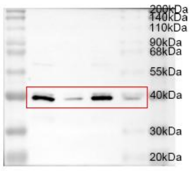

Figure S12A PANC-1 p-ERK

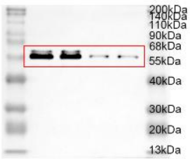

Figure S12A PANC-1 c-Myc

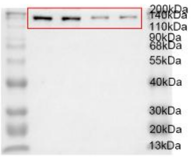

Figure S12A PANC-1 MAP4K4

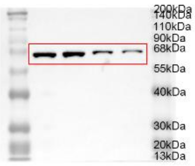

Figure S12A PANC-1 PAK1

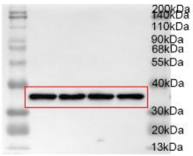

Figure S12A PANC-1 GAPDH

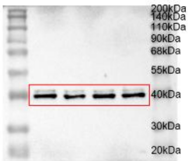

Figure S12A AsPC-1 total-ERK

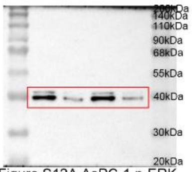

Figure S12A AsPC-1 p-ERK

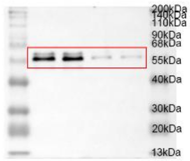

Figure S12A AsPC-1 c-Myc

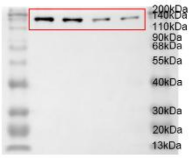

Figure S12A AsPC-1 MAP4K4

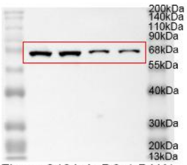

Figure S12A AsPC-1 PAK1

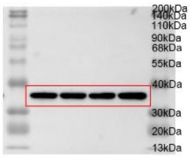

Figure S12A AsPC-1 GAPDH

Supplemental Figure 14A

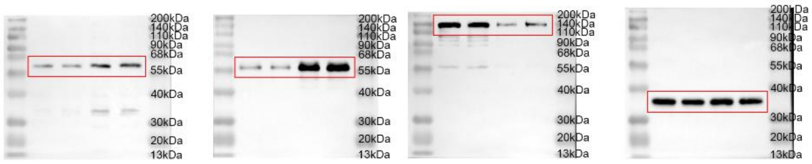

Figure S14A K148ac | Figure S14A c-Myc | Figure S14A HDAC5 | Figure S14A GAPDH

Supplemental Figure 14E

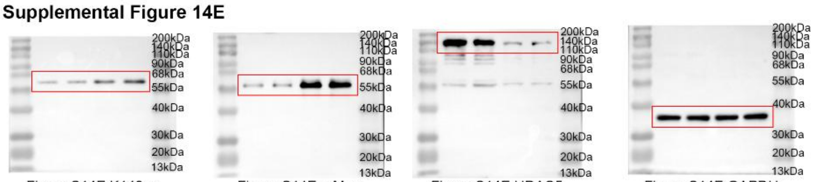

Figure S14E K148ac | Figure S14E c-Myc | Figure S14E HDAC5 | Figure S14E GAPDH

Supplemental Figure 18A

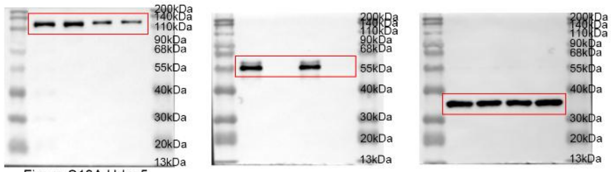

Figure S18A Hdac5 | Figure S18A c-Myc | Figure S18A Gapdh

Figure 18D

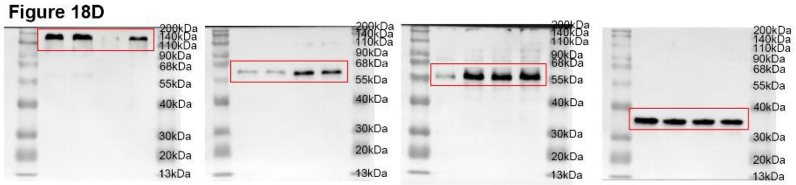

Figure 18D HDAC5 | Figure 18D K148ac | Figure 18D c-Myc | Figure 18D GAPDH
